# The lytic transglycosylase MltB connects membrane homeostasis and *in vivo* fitness of *Acinetobacter baumannii*

**DOI:** 10.1111/mmi.14000

**Published:** 2018-09-28

**Authors:** Sébastien Crépin, Elizabeth N. Ottosen, Katharina Peters, Sara N. Smith, Stephanie D. Himpsl, Waldemar Vollmer, Harry L. T. Mobley

**Affiliations:** ^1^ Department of Microbiology and Immunology University of Michigan Medical School Ann Arbor MI USA; ^2^ Centre for Bacterial Cell Biology, Institute for Cell and Molecular Biosciences Newcastle University Newcastle upon Tyne UK

## Abstract

*Acinetobacter baumannii* has emerged as a leading nosocomial pathogen, infecting a wide range of anatomic sites including the respiratory tract and the bloodstream. In addition to being multi‐drug resistant, little is known about the molecular basis of *A. baumannii* pathogenesis. To better understand *A*. *baumannii* virulence, a combination of a transposon‐sequencing (TraDIS) screen and the neutropenic mouse model of bacteremia was used to identify the full set of fitness genes required during bloodstream infection. The lytic transglycosylase MltB was identified as a critical fitness factor. MltB cleaves the Mur*N*Ac‐Glc*N*Ac bond of peptidoglycan, which leads to cell wall remodeling. Here we show that MltB is part of a complex network connecting resistance to stresses, membrane homeostasis, biogenesis of pili and *in vivo *fitness. Indeed, inactivation of *mltB *not only impaired resistance to serum complement, cationic antimicrobial peptides and oxygen species, but also altered the cell envelope integrity, activated the envelope stress response, drastically reduced the number of pili at the cell surface and finally, significantly decreased colonization of both the bloodstream and the respiratory tract.

## 1 INTRODUCTION

Worldwide, 700,000 deaths are associated with multi‐drug resistant infections per year. If no new antimicrobials are developed, it is estimated that by 2050, the number of deaths associated with these infections will reach 10 million per year, which would exceed those due to cancer and diabetes combined (Shallcross et al., 2015; Willyard, 2017). Recently, the World Health Organization (WHO) reported a list of drug‐resistant bacteria that pose a great threat to human health and for which new antimicrobials are needed (Lawe‐Davies and Bennett, 2017). Accordingly, *Acinetobacter baumannii* is considered as the number one priority among these bacterial pathogens.

*A. baumannii*, a gram‐negative, encapsulated bacterium, has emerged as a leading nosocomial pathogen, particularly in intensive care units specializing in respiratory care, trauma and burns (Wong et al., 2017). This bacterium infects a wide range of anatomic sites including the respiratory tract, bloodstream, wounds, urinary tract and meninges (Wong et al., 2017). The high prevalence of infection in immunocompromised, catheterized patients or those suffering from chronic lung diseases is concerning as multi‐drug resistance leaves few, or in some cases, no antimicrobial treatment options (Geisinger and Isberg, 2017; Wong et al., 2017). On average, 62,200 and 1,000,000 bacterial infections per year are caused by *A*. *baumannii *in the United States and worldwide respectively (Spellberg and Rex, 2013). Also alarming is its mortality rate, which is about 50% and 36% for ventilator‐associated pneumonia and bloodstream infections respectively (Fagon et al., 1996; Garnacho et al., 2003; Seifert et al., 1995; Wisplinghoff et al., 2004).

Extensive work has been performed to understand the mechanisms mediating drug resistance in *A*. *baumannii*. However, its pathobiology is not well understood. Indeed, just a subset of virulence factors has been identified thus far. Capsule, lipooligosaccharide, metal acquisition systems (iron and zinc), secretion systems (Type I, II and VI) and outer membrane proteins (OmpA, Omp33‐66) are among the virulence factors that have been identified (Harding et al., 2017; Wong et al., 2017). To better understand the virulence of *A*. *baumannii*, we and other groups have performed transposon‐based screening *in vivo *(Gebhardt et al., 2015; Subashchandrabose et al., 2016; Wang et al., 2014). While shedding some light on the fitness factors required during infections, these studies have used either a hypovirulent strain or an invertebrate model of infection.

To identify the full set of fitness genes required during bloodstream infection, we performed Transposon‐Directed Insertion site Sequencing (TraDIS) using the virulent, multi‐drug resistant bloodstream isolate AB0057 (Hujer et al., 2006) and a murine model of bacteremia (Smith et al., 2010). The lytic transglycosylase MltB was among the top fitness factors identified in the screen. MltB, a member of the lytic transglycosylase (LT) family, cleaves the glycosidic bond between *N*‐acetylmuramic acid (Mur*N*Ac) and *N*‐acetylglucosamine (Glc*N*Ac) residues of peptidoglycan (PG), concommittantly forming a 1,6‐anhydro bond in the Mur*N*Ac residue (Dik et al., 2017; Höltje et al., 1975; Scheurwater et al., 2008). These enzymes are involved in remodeling of the PG layer and releasing PG fragments (1,6‐anhydro‐muropeptides), and consequently, important for cell wall integrity (Dik et al., 2017). Recently, it was shown that LTs are important for pathogenesis in *Neisseria gonorrhoeae*, *Brucella abortus and Edwardsiella tarda *(Bao et al., 2017; Knilans et al., 2017; Liu et al., 2012; Ragland et al., 2017).

In the current study, we demonstrate that the lytic transglycosylase MltB is a critical fitness factor during bacteremia and pneumonia as it connects resistance to stresses, membrane homeostasis, biogenesis of pili and, ultimately, *in vivo *fitness.

## 2 RESULTS

### 2.1 *Transposon insertion sequencing screen for *in vivo *fitness genes*

To better understand the pathobiology of multi‐drug resistant *A*. *baumannii *(MDRAB) in a vertebrate model, TraDIS experiments were performed using the neutropenic murine model of bacteremia. Due to the limited genetic tools and markers available for use in the MDRAB AB0057 strain, we first engineered a kanamycin‐susceptible strain by creating an in‐frame, markerless, deletion‐mutant of the kanamycin resistance gene *AB57*_*0288*. When tested in the neutropenic murine model of bacteremia, this strain colonized the spleen and the liver as well as the parental strain AB0057 (Supporting Information Fig. S1). Since the *AB57*_*0288* mutant strain is as virulent as the parental strain, and is susceptible to kanamycin, in this study, we considered the *AB57*_*0288* mutant as the WT strain. Then, we used the EZ‐Tn5 transposome complex, along with the TypeOne™ Restriction Inhibitor (Epicentre), to generate a random transposon library of 49,628 mutants. The random distribution of the transposon across the chromosome was confirmed by sequencing 20 mutants (Supporting Information Table S1). In total, the library consists of 25,821 unique insertions and according to Goodman *et al.* (2011), an open reading frame was considered inactivated when at least three insertions were mapped into it.

To determine the full set of genes required during bloodstream infection, the transposon library was divided into five pools of 10,000 mutants and each pool was used to infect four mice each (20 mice in total). Twenty‐four hours post‐inoculation (hpi), the spleens and livers were collected, homogenized and samples from both organs were either plated for CFU enumeration (Supporting Information Fig. S2) or for genomic DNA isolation. Transposon‐gDNA junctions were amplified by PCR from the input and output pools and analyzed by Illumina sequencing to determine the relative abundance of each transposon mutant. Reads were mapped to the chromosome of strain AB0057 and a fitness index was calculated for each transposon mutant after passage into the bloodstream, as previously described by our group (Subashchandrabose et al., 2013; Subashchandrabose et al., 2016). By including the annotated pseudogenes, as well as the transposon insertions within 200 bp from the start codon of the gene, a total of 1,826 genes showed a fitness defect of at least two‐fold and a *p *value < 0.01 in the spleen (Supporting Information Table S2; Top 25 shown in Table 1). These broad criteria were chosen to identify any potential fitness factors, including the ones in which the transposon is inserted into the respective regulatory region. Although the number of candidate fitness factors seems high, Gebhardt *et al.* (2015), using the MDRAB AB5075 strain and the *Galleria mellonella *model of infection, found a comparable number of fitness factors having a fitness defect of at least 2.0 in their Data Set S1.

**Table 1:** Top 25 candidate fitness factors.

Locus tag	Function	Log_2_ Fitness defect^a^	*P* value
*AB57_3427*	sulfurtransferase	–10.82	0.0096
*AB57_0688*	2‐amino‐4‐hydroxy‐6‐hydroxymethyldihydropteridine pyrophosphokinase	–10.31	0.0078
*AB57_2595*	LuxR family transcriptional regulator	–10.18	0.0003
*AB57_3365*	hypothetical protein	–10.11	0.0034
*AB57_3288*	hypothetical protein	–9.66	0.0091
*AB57_0173*	hypothetical protein	–9.63	0.0052
*AB57_3551*	toluene tolerance protein	–9.60	0.0062
*AB57_3300*	hypothetical protein	–9.57	0.0001
*AB57_1860*	DNA‐binding protein HU‐beta	–9.52	0.0044
*AB57_1154*	hypothetical protein	–9.48	0.0075
*AB57_2881*	diadenosine tetraphosphatase	–9.47	0.0031
*AB57_3680*	acetyltransferase	–9.37	0.0062
*AB57_2749*	lytic transglycosylase	–9.36	0.0000
*AB57_2865*	NUDIX hydrolase	–9.24	0.0022
*AB57_0530*	50S ribosomal protein L33	–9.24	0.0001
*AB57_0531*	50S ribosomal protein L28	–9.24	0.0001
*AB57_0459*	hypothetical protein	–9.21	0.0052
*AB57_0336*	glutamyl‐Q tRNA(Asp) ligase	–9.17	0.0000
*AB57_3836*	phosphoserine phosphatase	–9.16	0.0035
*AB57_2526*	phosphoribosylamine–glycine ligase	–9.15	0.0001
*AB57_3700*	endonuclease	–9.15	0.0050
*AB57_RS18195*	hypothetical protein	–9.09	0.0094
*AB57_2745*	membrane protein	–9.08	0.0000
*AB57_0172*	PadR family transcriptional regulator	–9.08	0.0094
*AB57_0693*	hypothetical protein	–9.07	0.0038

Calculated fitness defect in the spleen at 24 hpi.

**Figure 1:**
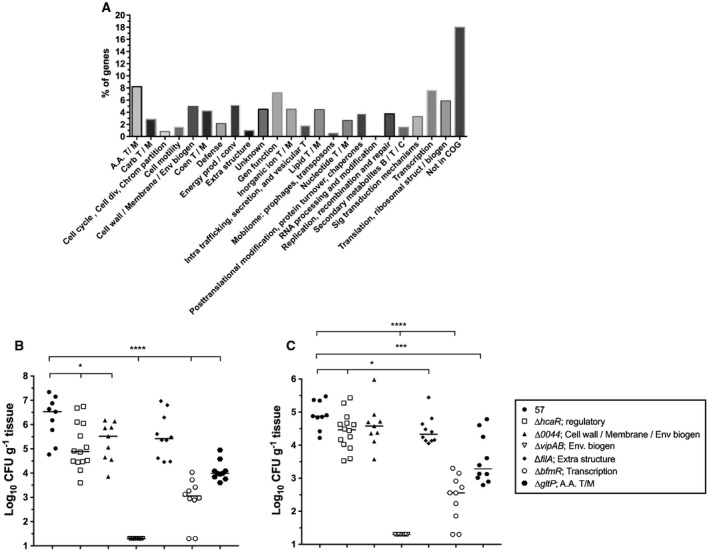
Validation of candidate AB0057 fitness factors during bloodstream infection. A. Classification of the candidate fitness factors required during bloodstream infection according to their Cluster of Orthologous Group (COG). B–C. Candidate fitness factors were confirmed using the neutropenic murine model of bloodstream infected. (B) Colonization of the spleen. (C) Colonization of the liver. CBA/J mice were infected with 10^7 ^CFU of either the WT (57) or the mutant strains by tail vein injection. At 24 hpi, mice were sacrificed, spleen and liver were harvested, and the bacterial burden was determined by CFU enumeration on LB agar. Bacterial numbers are presented as the log_10_ CFU g^–1^ of tissue. Each data point represents a sample from an individual mouse, and horizontal bars indicate the median values. Statistical significance was calculated by the Mann‐Whitney test (*p < 0.05; ***p < 0.0005; ****p < 0.0001). Abbreviation: A.A. T/M: Amino acids transport/metabolism, Carb T/M: Carbohydrates transport/metabolism, Cell div: Cell division, Chrom: Chromosome, Env biogen: Envelope biogenesis, Coen T/M: Coenzymes transport/metabolism, Prod/conv: production/conversion, Extra: extracellular, Gen: General, T/M: Transport/metabolism, Intra: Intracellular, T: Transport, B/T/C: Biosynthesis/transport/catabolism, Sig: Signal, Struct/biogen: Structure/biogenesis, 57: WT (AB0057^Km^).

The most representative functional categories of the putative fitness factors identified in the spleen included amino acid transport and metabolism, transcription, general function, translation, and cell wall, membrane and envelope biogenesis (Fig. 1A). To confirm the TraDIS analysis, deletion mutants of six candidate fitness factors among these categories, and having a broad range of fitness defects (Table 2), were constructed and their *in vivo *fitness was determined using the neutropenic murine model of bloodstream infection. Mono‐infections were performed by injecting 10^7^ CFU of either the WT or each mutant construct into the bloodstream of the neutropenic mouse via the tail vein. At 24 hpi, bacterial burden in the spleen and the liver was determined by CFU enumeration. By comparing the bacterial load between the WT strain and the mutant strains in these organs, five of six mutants showed a fitness defect, corresponding to a validation rate of ≥83% (Fig. 1B and C), which is typical of what we have previously observed in different Tn‐seq experiments in other species (Subashchandrabose et al., 2013; Bachman et al., 2015; Subashchandrabose et al., 2016; Anderson et al., 2017; Armbruster et al., 2017).

**Table 2:** Fitness factors validated in the neutropenic murine model of bacteremia.

Locus tag	Genes	Function/COG category	TraDIS FD^a^ in spleen	^b^FD in spleen	^b^FD in liver
*AB57_0486*	*hcaR*	Transcriptional regulator/Transcription	23	4 × 10^1^	2.5 × 10^0^
*AB57*_*0044*	*AB57*_*0044*	Lytic transglycosylase/Cell Wall, membrane and env. biogen.	352	1 × 10^1^	NS
*AB57*_*0094‐95*	*vipAB*	Vi polysacharide biosynthesis proteins/Cell wall, membrane and env. biogen.	436	10^5^	3.6 × 10^3^
*AB57_0739*	*filA*	Type III pili subunit/Extracellular structure	177	NS^c^	3.3x10^0^
*AB57_0796*	*bfmR*	Transcriptional regulator/Transcription	221	3 × 10^3^	2 × 10^2^
*AB57_1698*	*gltP*	Proton/sodium‐glutamate symport protein/A.A. T/M	50	3.5 × 10^2^	3.7 × 10^1^

Abbreviation: Env. biogen.: Envelope biogenesis, A.A. T/M: Amino acid transport and metabolism

FD; Fitness defect

Fitness defect was calculated by comparing the colonization burden between the WT and the mutant strain.

NS: not significant

### 2.2 *The lytic transglycosylase gene mltB* *encodes a fitness factor*

In addition to protecting cells from the environment, the cell envelope provides structural integrity to the cell and is associated with fitness in both *in vitro* and *in vivo *systems. To ensure its function, homeostasis of the cell envelope is tightly regulated. Due to its crucial importance, we were not surprised to observe 5% of the candidate fitness factors are involved in cell wall, membrane and envelope biogenesis. From this functional category, four genes belonging to the lytic transglycosylase family were identified as candidate fitness factors (Supporting Information Table S3), where the lytic transglycosylase *mltB* (*AB57_2749*) showed the greatest fitness defect in the spleen, 655‐fold, and overall, has the 13^th^ greatest fitness defect among all candidate fitness factors (Table 1). An *in silico* analysis showed that MltB of *A*. *baumannii *strain AB0057 has 41% amino acid sequence identity and 56% amino acid similarity with the *E*. *coli *homolog MltB. Furthermore, it is also predicted to possess the characteristic SLT_2 and MltB superfamily domains (Supporting Information Table S3).

**Figure 2:**
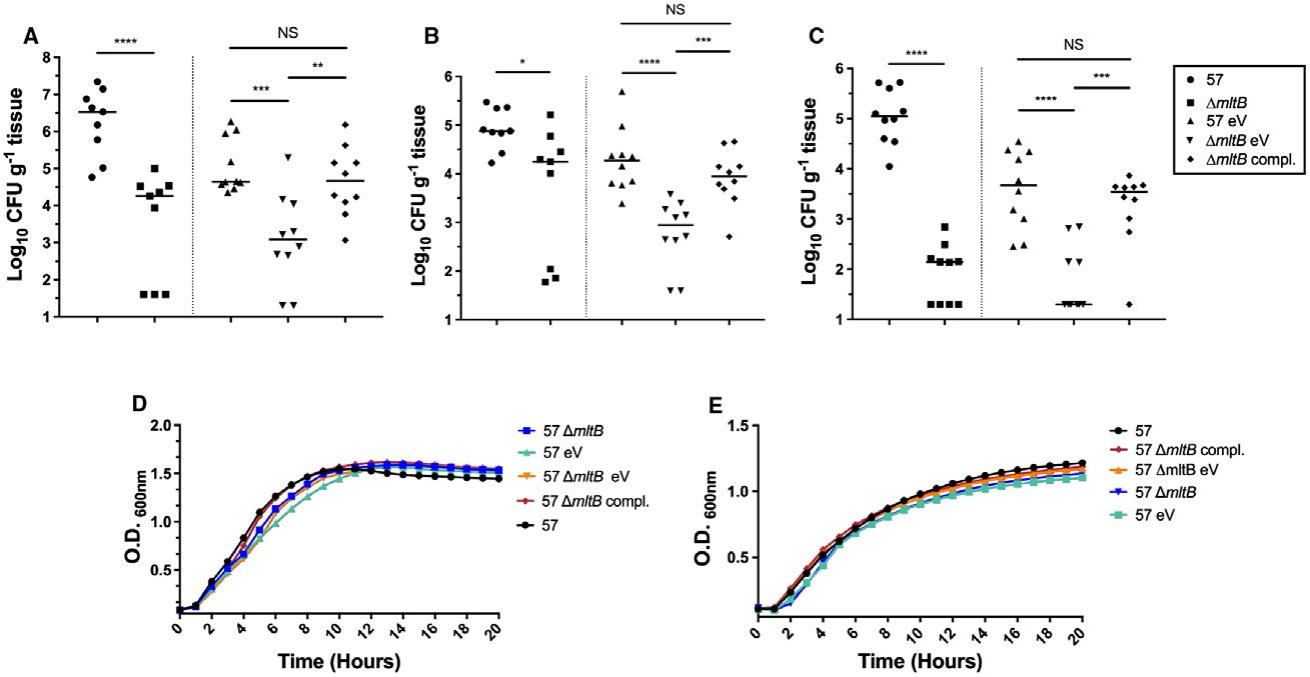
Colonization of the bloodstream by the *mltB* mutant. Colonization of the spleen (A), liver (B) and kidneys (C) was determined by infecting CBA/J mice with 10^7 ^CFU of either the WT strain (57) or its derivative strains. At 24 hpi, mice were sacrificed, organs were harvested, and the bacterial burden was determined by CFU enumeration on LB agar (57 and ∆*mltB*) and LB‐Km agar (57 eV, 57 ∆*mltB *eV and 57 ∆*mltB* compl.). Bacterial numbers are presented as the log_10_ CFU g^–1^ of tissue. Each data point represents a sample from an individual mouse, and horizontal bars indicate the median values. Statistical significance was calculated by the Mann‐Whitney test (*p < 0.05; **p < 0.01 ***p < 0.0005; ****p < 0.0001; NS, not significant). D and E. Growth of the WT (57) and its derivative strains. (D) LB. (E) M9 minimal medium supplemented with 0.4% Glucose and 0.2% casamino acids. Results from *in vitro *experiments are the mean values and standard deviations of three biological experiments. For ease of reading, standard deviations were removed from graphs D and E. Abbreviation: 57: WT (AB0057^Km^); eV: empty vector (pABBR_Km); compl.: complemented (pABBR_Km‐*mrdB*‐*mltB*).

By cleaving the β‐1,4 glycosidic bond between the Mur*N*Ac and the Glc*N*Ac residues of the PG, MltB is involved in the remodeling of PG and releasing of soluble fragments (Dik et al., 2017). Due to its role in maintaining the cell wall integrity, we sought to characterize the contribution of *mltB *in pathogenesis of *A*. *baumannii*. To confirm that *mltB *encodes a fitness factor *in vivo*, we constructed an in‐frame, markerless deletion mutant of *mltB *and tested whether its inactivation affects fitness in the neutropenic murine model of bloodstream infection. Mice were inoculated with 10^7 ^CFU of either the WT or the *mltB *mutant, and at 24 hpi, colonization of the spleen, liver and kidneys was determined by CFU enumeration. Compared to the WT strain, colonization of the spleen, liver and kidneys by the *mltB* mutant was decreased 185‐, 4‐ and 800‐fold respectively (Fig. 2A–C). Since *mltB* is the second gene of a two gene‐operon, downstream of *mrdB*, we complemented the mutation by cloning the entire operon with its native promoter into pABBR_Km and transformed the plasmid into the ∆*mltB *mutant. Complemention of the mutation *in trans* restored the WT fitness level in all organs (Fig. 2A–C) and confirmed that *mltB* encodes a crucial fitness factor in strain AB0057. Importantly, inactivation of *mltB *does not affect growth rate when cultured *in vitro* (LB and M9 supplemented with glucose and casamino acids) (Fig. 2D, E), demonstrating that attenuation of the *mltB* mutant *in vivo *was not simply due to a growth defect.

### 2.3 *MltB* *contributes to stress resistance*

**Figure 3:**
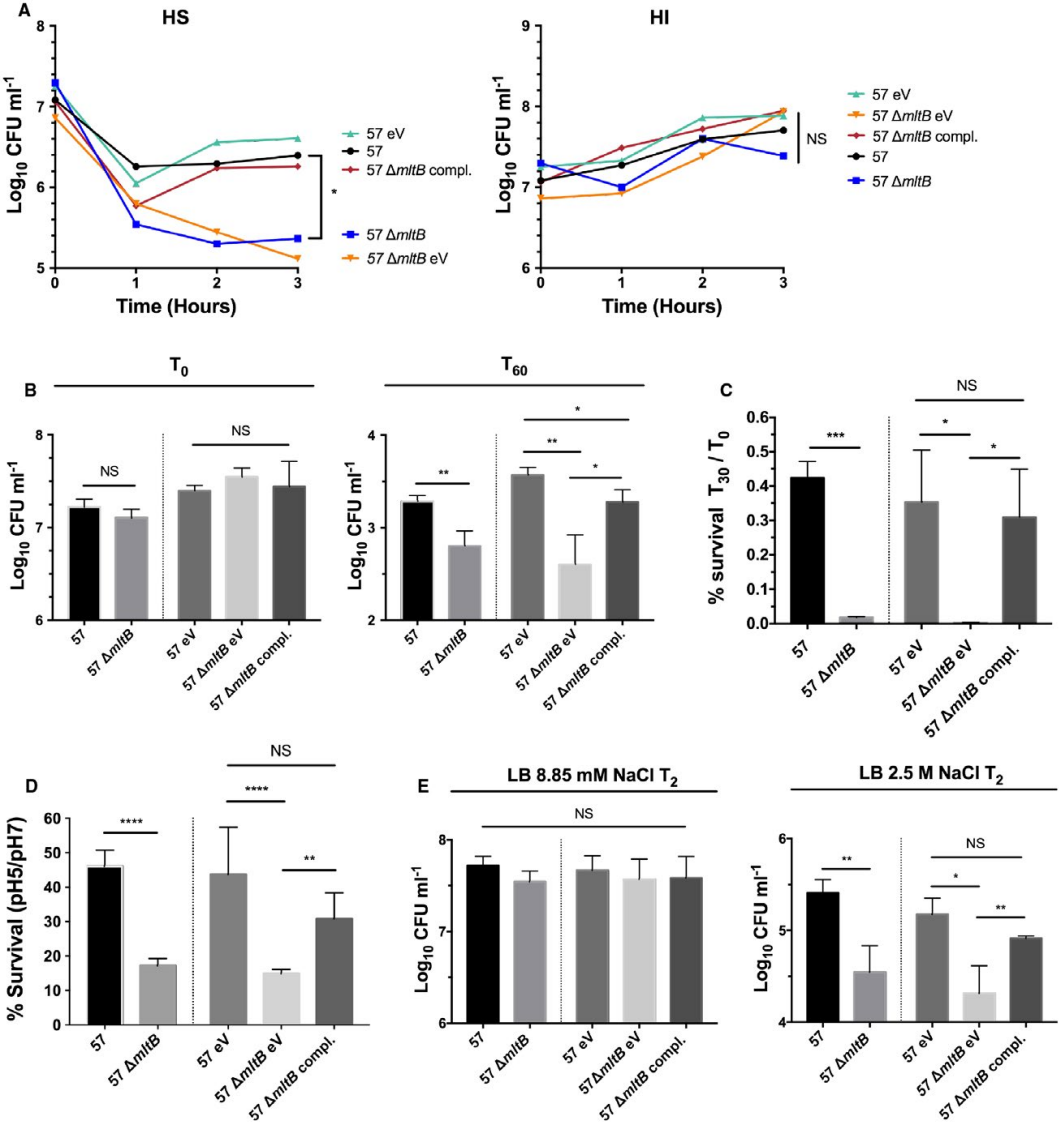
Resistance to stress by the *mltB *mutant. Resistance to stresses was determined by incubating 10^7^ CFU ml^–1^ of the WT (57) and its derivative strains to different stressors. A. Survival in 90% human active serum (HS) and growth in 90% heat‐inactivated human serum (HI). The number of surviving CFUs was quantified by CFU enumeration on LB agar every hr. For ease of reading, standard deviations were removed. B. Survival in the presence of 1 µg ml^‐1^ of polymyxin B. The number of CFUs recovered at 60 min (T_60_) was determined by CFU enumeration on LB agar and compared to time 0 (T_0_). C. Survival to oxidative stress (2.5 mM H_2_O_2_). Percent survival in 2.5 mM H_2_O_2 _was determined by dividing the number of CFU recovered at 30 min (T_30_) post‐inoculation by the number of CFUs at time 0 (T_0_). D. Survival in an acidic environment. The percent survival in an acidic environment was determined by dividing the number of CFU recovered at 60 min (T_60_) post‐inoculation in LB‐pH5 by the number of CFUs recovered in LB‐pH7. E. Survival to osmotic shock. The number of CFUs recovered in LB containing 8.85 mM or 2.5 M NaCl at 2 hr (T_2_) post‐inoculation was determined by CFU enumeration on LB agar. Results are the mean values and standard deviations of three independent experiments. Statistical significance was calculated by the Student’s *t*‐test (*p < 0.05; **p < 0.01; ***p < 0.005; NS: Not significant). Abbreviation: 57: WT (AB0057^Km^), eV: empty vector (pABBR_Km), compl.: complemented (pABBR_Km‐*mrdB*‐*mltB*).

As MltB is involved in peptidoglycan turnover and integrity, we hypothesized that its fitness defect in the bloodstream is associated with increased susceptibility to stresses including bactericidal activity of serum, cationic antimicrobial peptides, oxidative and acid stresses, and osmotic shock. To test this hypothesis, we first sought to determine whether the ∆*mltB *mutant was more susceptible to the bactericidal activity of serum by incubating 10^7^ CFU ml^–1 ^of the WT, ∆*mltB *and complemented strains with 90% active human serum. The number of CFU was monitored every 60 min for a period of 3 hr. At 3 hpi, the number of CFU recovered from the *mltB* mutant was 10‐fold lower than the WT and the complemented strain (Fig. 3A; HS). To validate whether the increase in susceptibility of the ∆*mltB* mutant to human serum was mediated by its complement‐mediated bactericidal activity and not to a decreased fitness in serum itself, the strains were incubated in 90% heat‐inactivated human serum for 3 hr. At 3 hpi, no difference in viability was observed between the WT, the *mltB* mutant and the complemented strain (Fig. 3A; HI), confirming that the increased susceptibility of the mutant construct was due to the bactericidal activity of complement.

Capsule is among the factors contributing to pathogenesis and resistance to serum, notably by interfering with opsonophagocytosis and complement‐mediated killing (Merino et al., 1992; Whitfield, 2006; Bachman et al., 2015; Diao et al., 2017; Anderson et al., 2017). To test whether the increased human serum susceptibility of the ∆*mltB* mutant was due to a defect in capsule production, we used the Maneval’s stain coupled with light microscopy (Maneval, 1941) and the mucoviscosity assay (Bachman et al., 2015) to assess its production among the WT, ∆*mltB* and the complemented strains. Although slight differences were observed by microscopy between the strains, the ∆*mltB* mutant is 2.25‐times less mucoviscous than the WT strain and complementation of the mutation restored the mucoviscosity to the WT level (Supporting Information Fig. S3). The contribution of *mltB *in resistance to stresses was also validated in the MDRAB strain AB5075 and its isogenic *mltB* mutant. First, to confirm the role of *mltB* to the bactericidal activity of the human serum, we tested the survival rate to 90% active human serum as well as their growth in heat‐inactivated serum. At 3 hpi, the number of CFU recovered from the *mltB* mutant was 327‐times fewer than the AB5075 strain (Supporting Information Fig. S4A; HS). Susceptibility of the AB5075 ∆*mltB *strain to human serum was also due to the complement‐mediated bactericidal activity, as no difference in growth was observed between the mutant strain and AB5075 in heat‐inactivated human serum (Supporting Information Fig. S4A; HI). As for the AB0057 strain, the *mltB* mutant of strain AB5075 was 2.43‐less mucoviscous than the WT strain (Supporting Information Fig. S5).

To defend itself against infection, the host secretes antimicrobial peptides as part of its innate defense system. We tested whether the ∆*mltB *mutant was more susceptible to polymyxin B, a cationic antimicrobial peptide that disrupts membrane integrity. Therefore, 10^7^ CFU ml^–1^ of the WT, ∆*mltB *and complemented strains were incubated with 1 µg ml^‐1^ of polymyxin B for 60 min. As expected, the *mltB *mutant was more susceptible than the WT strain as the number of CFU recovered from the *mltB *mutant was 3.1‐fold lower while complementation of the mutation restored the number of CFU to the WT level (Fig. 3B). Similarly, inactivation of *mltB* in strain AB5075 increased susceptibility to polymyxin B 3.2‐fold compared to the WT strain (Supporting Information Fig. S4B).

The host also protects itself against infection by the production of oxidative, acid and osmotic stresses. To address the contribution of *mltB *in response to these stresses, we challenged the WT, ∆*mltB* and complemented mutant with H_2_O_2_, HCl and high concentrations of NaCl. First, the AB0057 and the *mltB *mutant were incubated in the presence of 2.5 mM H_2_O_2_ for 30 min. Under this condition, the *mltB *mutant was 24.7‐times more sensitive to H_2_O_2_ compared to the WT strain, and complementation of *mltB* *in trans *restored the number of CFU of the mutant strain to the WT level (Fig. 3C). In agreement, the ∆*mltB* mutant of AB5075 was 17‐times more susceptible than the WT strain to H_2_O_2_ (Supporting Information Fig. S4C).

To test whether the *mltB *mutant is more susceptible to acid, the WT, ∆*mltB* and the complemented mutant were cultured in LB‐pH5 and survival in acidic environment was evaluated by comparing to their growth in LB‐pH7. At 1 hpi, the *mltB* mutant showed a 2.7‐fold lower survival rate at low pH than the WT strain. Indeed, the percent survival of the *mltB *mutant in LB‐pH5 was 17.2%, while the WT strain was 46.1%. In addition, survival of the complemented mutant at low pH was 30.8%, which partially restored survival of the *mltB* mutant to the WT level (Fig. 3D). In strain AB5075, the same trend was observed as the *mltB *mutant was two‐times more susceptible to low pH than the WT strain (Supporting Information Fig. S4D).

Finally, we tested whether the mutant strain was more susceptible to osmotic shock. To do so, the WT and the ∆*mltB *mutant were incubated for 2 hr in the presence of 2.5 M NaCl and the number of surviving cells was enumerated on LB agar. Under this condition, the *mltB *mutant was 7.3‐times more susceptible to high osmolarity than the WT strain cells (Fig. 3E). In addition to calculating the survival rate in high osmolarity, we wanted to determine the growth rate of the ∆*mltB* mutant in the presence of different concentration of NaCl. Strains were cultured in LB containing 100, 250, 500 and 750 mM NaCl and the growth rate was monitored by measurement of OD_600_ every 30 min. Although no growth defect was observed at 100 and 250 mM NaCl, the ∆*mltB* mutant was unable to grow at 500 and 750 mM NaCl (Supporting Information Fig. S6); complementation of the mutation restored the growth rate of the *mltB *mutant to the WT level (Fig. 3E and Supporting Information Fig. S6). Surprisingly, *mltB* did not seem to contribute to osmotic shock in the AB5075 strain as the *mltB* mutant was as resistant to high osmolarity as the WT strain (Supporting Information Fig. S4E and S7).

Taken together, these results demonstrated that the sensitivity of the *mltB* mutant to stresses may explain, at least in part, its fitness defect observed during bloodstream infection.

### 2.4 *MltB* *contributes to the cell envelope homeostasis*

**Figure 4:**
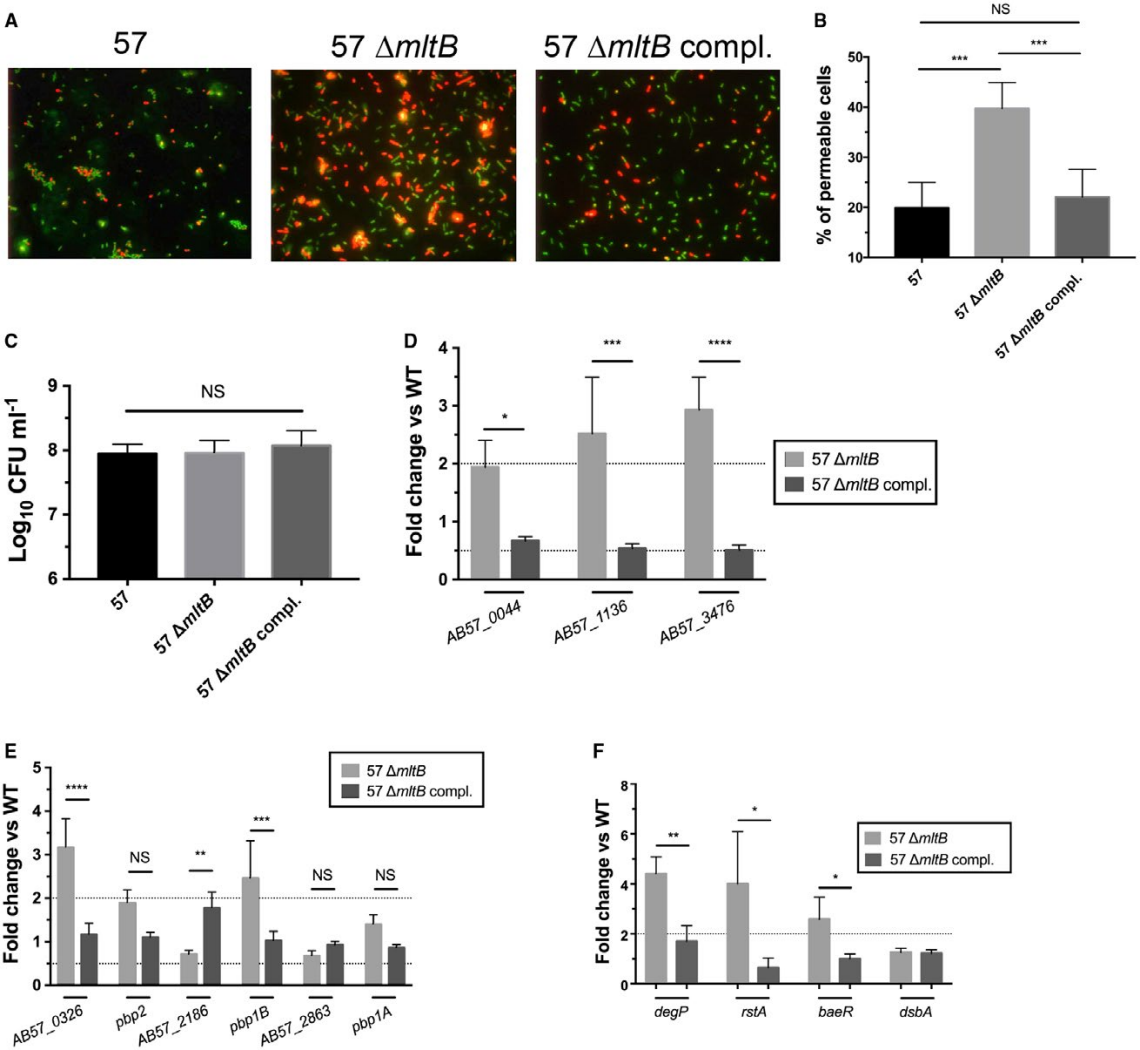
Membrane homeostasis is altered in the *mltB *mutant. A. Visualization of membrane permeability using fluorescent microscopy. The WT (57) and its derivative strains were stained with a mix of Syto 9 and propidium iodide dyes. Syto 9 dye (green) stains the nucleic acid of all bacteria while the propidium iodide (red) stains the nucleic acid of permeable cells. Images are representative of three independent experiments. B. Percentage of permeable cells was calculated by dividing the number of permeable cells (red) by the total number of bacteria. C. Cell viability from panel (A) and (B) was determined by CFU enumeration after the Syto 9 and propidium iodide staining. D–E–F. Gene expression between the WT, ∆*mltB* and the complemented strain. (D) Lytic transglycosylases. (E) Penicillin‐binding protein. (F) The envelope stress response (ESR). Gene expression was evaluated by qRT‐PCR and compared between the WT, ∆*mltB* and the complemented strain. The dashed line corresponds to the cutoff for a significant difference in expression. All results are the mean values and standard deviations of three independent experiments. Statistical significance was calculated by the Student’s *t*‐test (B and C) and by two‐way ANOVA with Sidak’s multiple comparisons test (D, E and F) (*p < 0.05; **p < 0.01; ***p < 0.0005; ****p < 0.0001; NS, not significant). Abbreviation: 57, WT (AB0057^Km^); eV: empty vector (pABBR_Km); compl., complemented (pABBR_Km‐*mrdB*‐*mltB*).

Since the *mltB* mutant has a fitness defect *in vivo* and is more susceptible to stresses targeting the cell envelope, we hypothesized that the *mltB *mutant would have altered membrane integrity and consequently, be subject to an envelope stress response (ESR). To test this hypothesis, we first determined whether the membrane of the *mltB* mutant was more permeable than the WT strain by performing a propidium iodide assay. A mix of Syto 9 and propidium iodide dyes, where Syto 9 (green) stains the nucleic acid all cells and propidium iodide (red) only stains the nucleic acid of damaged (permeable) membrane cells, was used to score the % of permeable cells. By calculating the number of propidium iodide positive cells (red) over the total number of cells, we observed that the membrane of the *mltB* mutant is two‐times more permeable than the WT strain (Fig. 4A and B), and complementation restored the membrane permeability of the mutant strain to the WT level. To confirm that the increase in the number of permeable cells in the *mltB *mutant was due to an increased permeability of the membrane, and not to an increased cell death, the number of CFU following the staining was enumerated on LB agar plates. As shown in Fig. 4C, no difference was observed between strains, confirming the increased membrane permeability in the *mltB *mutant.

In *E*. *coli*, it has been observed that LTs possess extensive functional redundancy (Heidrich et al., 2002; Lee et al., 2013). Since, the genome of AB0057 encodes four predicted lytic transglycosylases (LTs) (Supporting Information Table S3) (Hamidian et al., 2017), we sought to determine whether the other LTs were induced or repressed in the *mltB *mutant as a compensatory mechanism that might explain the increase membrane permeability of the *mltB* mutant. Expression of *AB57_0044*, *AB57_1136* and *AB57_3476* was quantified by qRT‐PCR and was compared between the WT, the ∆*mltB* mutant and the complemented strain. Genes *AB57_1136* and *AB57_3476* were induced 2.52‐ and 2.93‐fold respectively, in the ∆*mltB *mutant, and complementation of the mutation restored the WT expression level (Fig. 4D).

Penicillin‐binding proteins (PBP) are a major factor in cell wall biosynthesis. Indeed, the glycosyltransferase and transpeptidase domains of PBP catalyze the final steps of the growth of the PG layer thus conferring its 3D structure (Sung et al., 2009; Typas et al., 2011). Since PBPs are important for cell envelope integrity, and this integrity is altered in the ∆*mltB *mutant, we hypothesized that expression of PBP genes was affected in the *mltB *mutant. To test this hypothesis, expression of *AB57_0326*, *pbp2*, *AB57_2186*, *pbp1B*, *AB57_2861* and *pbp1A* was determined by qRT‐PCR and was compared between the WT, the *mltB* mutant and the complemented strain. As expected, in the *mltB *mutant, genes *AB57_0326* and *pbp1B *were induced 3.17‐ and 2.46‐fold respectively, and complementation of the mutation restored the WT expression level (Fig. 4E).

To determine whether inactivation of *mltB* affected the peptidoglycan structure, the muropeptides from the WT, ∆*mltB* and the complemented strains were prepared following growth in LB. To mimic growth in the bloodstream, strains were also grown in 50% heat‐inactivated human serum. The extracted muropeptides were then separated by high‐performance liquid chromatography to quantify any difference between strains (Glauner, 1988). As shown in Supporting Information Table S4, no major differences in the muropeptides composition were observed between the WT and the ∆*mltB* mutant in both conditions, suggesting that inactivation of *mltB* induces subtle changes in PG composition.

The ESR is a system that senses environmental changes and stresses; and prompts the cell to respond appropriately (Leblanc et al., 2011). Since, the ∆*mltB* mutant is more susceptible to stresses and shows altered membrane integrity, along with the induction of LTs and PBPs, we tested whether the ESR was induced in the *mltB* mutant. The ESR has been extensively studied in *Enterobacteriaceae *(Macritchie and Raivio, 2009; Cabeza et al., 2007; Guest and Raivio, 2016). However, this stress response is not well understood in *A. baumannii*. By screening homologues of the ESR members in *A. baumannii*, we quantified, by qRT‐PCR, expression of *degP*, *rstA*, *baeR* and *dsbA* in the *mltB *mutant. When compared to the WT strain, expression of *degP*, *rstA* and *baeR* was induced 4.4‐, 4.0‐ and 2.6‐fold in the *mltB *mutant, while expression of *dsbA* was not different from the WT strain (Fig. 4F). Complementation of the *mltB* deletion restored expression of *degP*, *rstA* and *baeR* to the WT level, which demonstrates that inactivation of *mltB* induced the ESR.

Taken together, these results demonstrate the contribution of *mltB *in maintenance of the cell envelope homeostasis in *A*. *baumannii*. In addition, these results may explain, at least in part, the increased sensitivity to stresses as well as the fitness defect observed in the neutropenic murine model of bacteremia.

### 2.5 *MltB* *influences binding to abiotic surfaces and epithelial cells*

**Figure 5:**
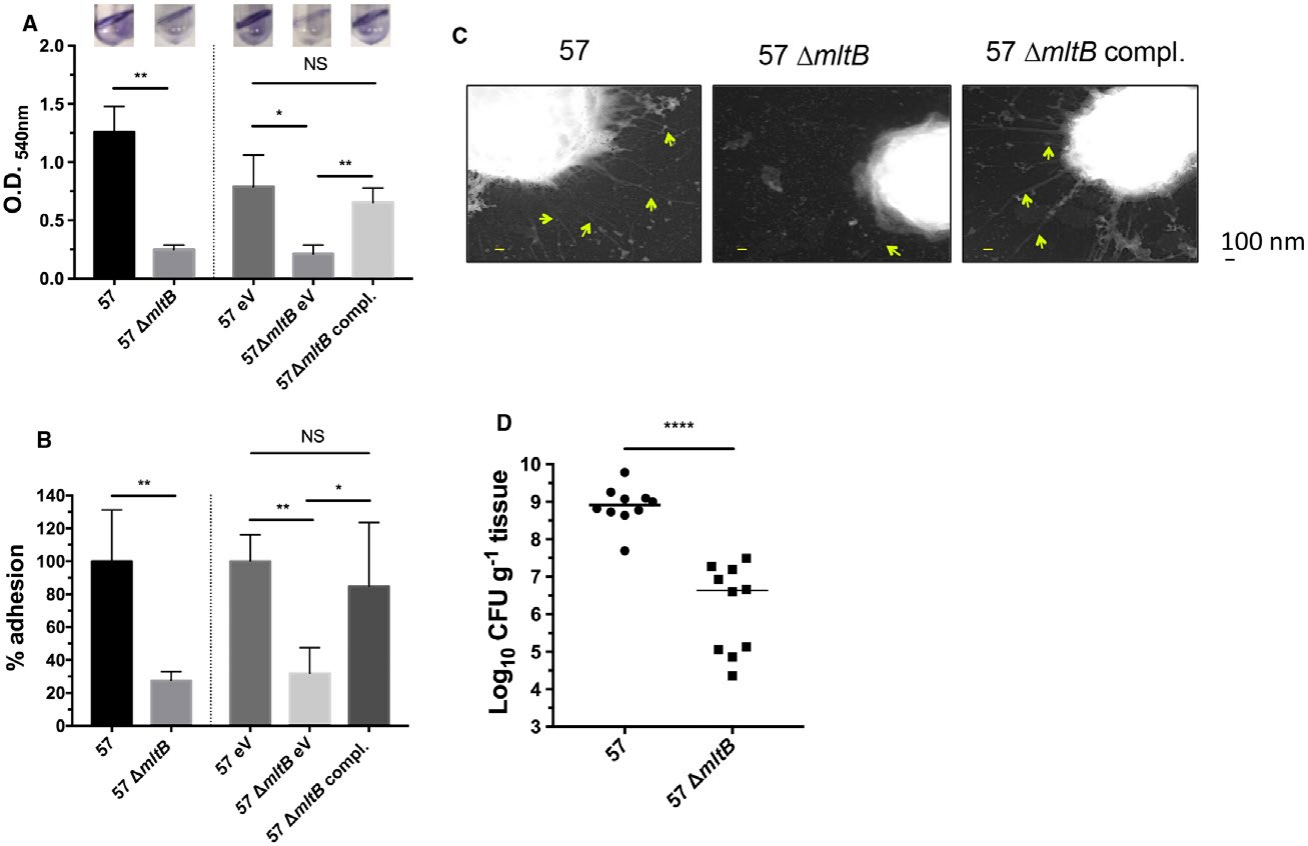
Pili assembly in the *mltB* mutant of strain AB0057. A. Biofilm formation in LB at 30°C for 24 hr under static conditions. Crystal violet binding assay was used to monitor biofilm formation. B. Adherence to A549 lung epithelial cells. C. Transmission electron microscopy of the WT (57) and its derivative strains at magnification 20,000×. Images show a typical field of view. Arrows show location of the pili on cell surfaces. D. Colonization of the lungs by the WT (57) and its isogenic *mltB *mutant. CBA/J mice were infected by intranasal aspiration with 10^7 ^CFU of either the WT (57) or the *mltB *mutant. At 24 hpi, mice were sacrificed, lungs were harvested, and the bacterial burden was determined by CFU enumeration on LB agar. Bacterial numbers are presented as the log_10_ CFU g^–1^ of tissue. Each data point represents a sample from an individual mouse, and horizontal bars indicate the median values. Results from *in vitro *experiments are the mean values and standard deviations of three biological experiments. Statistical significance was calculated by the Student’s *t*‐test (A and C), and by the Mann–Whitney test (E) (*p < 0.05; **p < 0.01; ***p < 0.0001; NS, not significant). Abbreviations: 57, WT (AB0057^Km^); eV, empty vector (pABBR_Km); compl., complemented (pABBR_Km‐*mrdB*‐*mltB*).

Membrane homeostasis is important for proper assembly and function of membrane‐bound structures. Since this homeostasis is altered in the *mltB* mutant, we hypothesized that assembly of pili at the cell surface, for example, would be perturbed in the *mltB* mutant. As pili and adhesins are important factors contributing to binding to both abiotic and biotic surfaces, we first sought to determine, using the crystal violet binding assay, whether the *mltB* mutant was less able to bind to an abiotic surface and form a biofilm on polystyrene surface. When cultured at 30°C in LB for 24 hr under static conditions, the *mltB* mutant was 5.0‐times less able to form a biofilm than the WT and complemented strains (Fig. 5A). Similarly, inactivation of *mltB* in strain AB5075 reduced biofilm formation 2.7‐fold compared to the WT strain (Supporting Information Fig. S8A).

We then wanted to determine whether inactivation of *mltB* influenced adhesion to host epithelial cells. To address this question, we performed adhesion assay on the human alveolar basal epithelial cells A549. Two hours after addition of bacteria to the tissue culture cells, the ∆*mltB* mutant adhered to epithelial cells in 3.7‐times fewer numbers than the WT strain, and complementation restored adhesion of the mutant strain to the WT level (Fig. 5B). This was also true for strain AB5075, as inactivation of *mltB* reduced adhesion to epithelial cells 3.5‐fold compared to the WT strain (Supporting Information Fig. S8B).

It was recently shown that, instead of adhering to the tissue culture cells, some *A*. *baumannii* strains preferentially bind to inert surfaces (Lazaro‐Diez et al., 2016). To confirm that the decreased adherence of the *mltB* mutant to A549 cells was actually associated to a defect in binding to the cell line, instead of to the polystyrene surface of the wells, the adherence assay was performed as described above with the exception that no cells were present in the wells. Two hours after addition of the bacteria to the wells, adhesion to the polystyrene surface was quantified, as above, and no significant difference was observed between the WT, *mltB* mutant and the complemented strain (Supporting Information Fig. S9A). Accordingly, no significant difference was observed between the WT strain of AB5075 and its *mltB *isogenic mutant (Supporting Information Fig. S9B). These results confirm that the adhesion defect of the *mltB* mutant is associated to the decrease ability to bind to A549 cells, and not to the wells of the microtiter plates used.

Since biofilm formation and adhesion to epithelial cells were both decreased in the *mltB* mutant, we hypothesized that *mltB *is important for assembly of pili at the cell surface. Accordingly, we hypothesized that inactivation of *mltB *reduces the presence of these structures at the cell surface. To test this hypothesis, transmission electron microscopy was performed on the WT, ∆*mltB* mutant, and complemented strains. The WT and the complemented strains presented long appendages, consistent with pili (Alvarez‐Fraga et al., 2016; Moon et al., 2017) at their cell surface. The numbers of pili‐like structures were drastically reduced in the ∆*mltB* mutant (Fig. 5C), confirming the role of *mltB* in assembly of pili at the cell surface.

### 2.6 *MltB* *also significantly contributes to colonization of the respiratory tract*

As the number of pili at the cell surface was drastically reduced in ∆*mltB*, which affected its biofilm formation and adhesion to alveolar epithelial cells, we wondered whether the *mltB *mutant had a fitness defect during pneumonia. Mouse pulmonary infection was induced by nasal aspiration with 10^7 ^CFU per mouse and at 24 hpi, the lungs were harvested, and the colonization burden was evaluated by CFU enumeration. As expected, the ∆*mltB* strain colonized the lungs with 192‐times fewer CFU than the WT strain (Fig. 5D). As for during bloodstream infection, these results confirm the role of *mltB* in colonization of the host.

Taken together, these results show that in addition to decrease resistance to stresses and to increase membrane permeability, inactivation of *mltB* also affects assembly of pili at the cell surfaces, which could be connected to inhibition of biofilm formation, adhesion to host epithelial cells and ultimately, may explain the fitness defects in the murine model of bloodstream and pneumonia infections.

## 3 DISCUSSION

With its high infection prevalence, mortality rate and resistance to multiple antibiotics, *A. baumannii* has emerged as a pathogen of concern that poses a serious threat to human health. Indeed, among the bacteriological pathogens for which new antimicrobials are urgently needed, *A*. *baumannii* is the number one priority according to the WHO (Lawe‐Davies and Bennett, 2017; Willyard, 2017). Despite having 79 complete genomes and 1795 contig sequences of multiple strains of *A. baumannii,* the identity of the genes essential for pathogenesis in mammalian hosts is not well known. Indeed, the mechanisms by which *A*. *baumannii* colonizes the host, avoids the immune system, and incites tissue damage are not yet well defined. By identifying and characterizing the fitness factors required for survival *in vivo*, we will be able to design strategies to combat its infections.

To unveil the fitness factors important for colonization of the host, we used a combination of transposon‐based screening (TraDIS) (Langridge et al., 2009) and the neutropenic murine model of bacteremia (Smith et al., 2010). Herein, we identified a total of 1,826 putative fitness factors (Supporting Information Table S2). Although this number seems high, Gebhardt *et al*. (2015), in their Data Set S1, found a similar number of fitness factors having a fitness defect of at least 2.0 in the MDRAB AB5075 strain using the *G*.* mellonella *model of infection. In addition to identifying known fitness factors, such as the Type I and II Secretion Systems (Johnson et al., 2015; Harding et al., 2016; Harding et al., 2017), iron and zinc acquisition systems (Gaddy et al., 2012; Hood et al., 2012; Mortensen et al., 2014; Subashchandrabose et al., 2016) and capsule synthesis (Russo et al., 2010; Gebhardt et al., 2015; Geisinger and Isberg, 2015), which we confirmed in our screen, we also identified novel fitness factors (Supporting Information Table S2), such as the lytic transglycosylase MltB.

By comparing the candidate fitness factors identified in our study with other transposon‐based screening, 109‐ and 46‐ genes were common to the ATCC17978 strain identified in the pneumonia (Wang et al., 2014) and bacteremia (Subashchandrabose et al., 2016) models of infection respectively (Supporting Information Table S5). The use of different strains (hypovirulent vs. virulent), model of infection (cyclophosphamide vs. RB6‐5C6 treated mice) and preparation of the inoculum prior to infection may be among the factors explaining the discrepancies between our current study and the one we previously published (Subashchandrabose et al., 2016). Interestingly, by comparing the putative fitness factors identified in MDRAB AB5075 strain from the *G*.* mellonella* larvae model of infection (Gebhardt et al., 2015), 628 genes were in common with ours (Supporting Information Table S5). The *mltB *gene is among them.

Herein, we determined the contribution of the lytic transglycosylase *mltB* to *in vivo* fitness of *A*. *baumannii* AB0057. First, we confirmed that *mltB* encodes an important fitness factor since its inactivation highly compromises colonization of the bloodstream (Fig. 2A–C) and the respiratory tract (Fig. 5D), but does not affect *in vitro* growth rate (Fig. 2D, E). Second, we showed that *mltB* is important for resistance to stresses associated to bloodstream infection (Fig. 3 and Supporting Information Fig. S6). Third, we demonstrated that *mltB* contributes to cell envelope integrity and to a lesser extent, capsule production (Fig. 4 and Supporting Information Fig. S3). Finally, we presented evidences that* mltB* influences assembly of pili at the cell surface, affects biofilm formation, as well as adherence to human alveolar basal epithelial cells (Fig. 5). The phenotypes described above were also validated in the MDRAB strain AB5075 (Supporting Information Fig. S4, S5 and S8).

The contribution of LTs to pathogenesis was recently observed in *N. gonorrhoeae*, *B. abortus and E. tarda *(Liu et al., 2012; Bao et al., 2017; Knilans et al., 2017; Ragland et al., 2017). However, the molecular mechanisms connecting LTs to pathogenesis are not well defined, especially in *A*. *baumannii*. Here, we showed that inactivation of *mltB* impaired resistance to stresses associated with bloodstream infection, such as the bactericidal activity of serum, cationic antimicrobial peptide, oxidative and acid stresses as well as to osmotic shock (Fig. 3 and Supporting Information Fig. S4 and S6). Since the host elicits similar stresses to combat infection, the increased susceptibility of the *mltB *mutant to these stresses may help explain, at least in part, its decreased virulence in the murine model of bloodstream and pulmonary infections. Our results are also in agreement with what was observed in *N. gonorrhoeae* and *E. tarda*. Indeed, in *N. gonorrhoeae*, a *lgtA*‐*lgtD* double mutant is more susceptible to lysozyme and neutrophil elastase (Ragland et al., 2017), while in *E*. *tarda*, inactivation of *mltA* reduces survival in minimal medium, as well as increasing susceptibility to high osmolarity (Liu et al., 2012).

The cell envelope protects the cells against environmental insults, such as the immune system, temperature, pH, osmolarity, toxic compounds and antibiotics (Guest and Raivio, 2016). To adapt to these assaults, bacteria have evolved several ESRs to sense these attacks, monitor defects or damages and to restore the cell envelope homeostasis (Grabowicz and Silhavy, 2017). Accordingly, along with the increased susceptibility to stresses, the *mltB* mutant presents signs of alteration of its cell envelope integrity. Indeed, inactivation of *mltB *increases membrane permeability and induces the ESR (Fig. 4A, B and F). Our data are also in agreement with what was observed in *N*. *gonorrhoeae *and *Pseudomonas aeruginosa* in regard to membrane permeability (Lamers et al., 2015; Ragland et al., 2017). However, the mechanisms by which inactivation of LTs leads to increased membrane permeability are unclear.

To determine whether inactivation of *mltB* affects the composition of the PG, and explains the alteration of the cell envelope integrity of the mutant strain, a complete analysis of the PG composition between the WT, ∆*mltB* and the complemented strains was performed (Supporting Information Table S4). Not surprisingly, no obvious difference between the WT and the *mltB* mutant were observed. Accordingly, other groups observed that inactivation of LTs or PBPs may not dramatically affect the composition of the PG (Jorgenson et al., 2014; Boll et al., 2016; Kohler et al., 2007). It is possible that inactivation of *mltB* induces subtle changes in the peptidoglycan structure that we could not detect. Since it is proposed that LTs are functionally equivalent (Koraimann, 2003, Lee et al., 2013; Scheurwater et al., 2008; Wu et al., 2016; Dik et al., 2017), it is possible that a compensatory mechanism is activated in the *mltB *mutant to overcome the loss of *mltB* and may explain the absence of difference in the PG composition between the WT and the *mltB *mutant. Indeed, expression of two LTs (*AB57_1136 *and *AB57_3476*) were induced in the *mltB* mutant (Fig. 4D).

In addition, we also observed that two PBP (*AB57_0326* and *pbp1B*) and the ESR were induced in the *mltB* mutant as well (Fig. 4E, F). In *E. coli*, it was observed that LTs, PBPs and the ESR are interconnected. Indeed, activation of the ESR induces expression of the LT gene *slt *(Bernal‐Cabas et al., 2015), while inactivation of PBPs activates the ESR (Bernal‐Cabas et al., 2015). It is proposed these three systems are part of a complex regulatory network involved in maintaining the cell envelope integrity. Our results strongly support this hypothesis since inactivation of *mltB* induces expression of two LTs, two PBPs and activates the ESR. Thus, the molecular mechanisms connecting *mltB*, LTs, PBPs and the ESR, as well as their contribution to the phenotypes observed in the ∆*mltB *mutant remain to be determined.

In addition to being involved in membrane homeostasis, it is postulated that peptidoglycan degrading enzymes, such as LTs and endopeptidases, act as bacterial ‘space‐making’ autolysins (Scheurwater et al., 2008; Stohl et al., 2013). Accordingly, it was observed they are required for assembly of flagella and/or pili and in *Caulobacter crescentus*, *Neisseria gonorrhoeae*, and *Rhodobacter sphaeroides* (Viollier and Shapiro, 2003; Stohl et al., 2013; Herlihey et al., 2016), the type III secretion system in *Xanthomonas* *campestris* pv. Vesicatoria (Hausner et al., 2017) and the type VI secretion system in *E*. *coli* and *A*. *baumannii *(Weber et al., 2016; Santin and Cascales, 2017). Given these observations, it was not surprising to note that the ∆*mltB *mutant was devoid of pili at its cell surface (Fig. 5C). Lack of pili at the cell surface of the *mltB* mutant appears to be associated to its reduction in biofilm formation as well as adherence to human alveolar basal epithelial cells A549 (Fig. 5A, B), and may explain, at least in part, its decreased *in vivo *fitness. In addition, in *N*. *gonorrhoeae*, it was observed that inactivation of the dd‐carboxypeptidase and endopeptidase *NGO1686* gene, encoding a peptidoglycan degrading enzyme, increased susceptibility to H_2_O_2_, which is associated with the lack of piliation of the mutant strain (Stohl et al., 2012; Stohl et al., 2013). In our study, it may be possible that in the ∆*mltB *mutant, the increased sensitivity of the *mltB *mutant to H_2_O_2_ is due to the dramatic decrease of pili at its cell surface. The contribution of these pili to adherence, resistance to H_2_O_2_ as well as in pathogenesis remains to be determined.

In summary, in this study we have demonstrated that *mltB* encodes an important fitness factor during bloodstream and pulmonary infections. In addition, we have shown that *mltB* is part of a complex network connecting membrane homeostasis, resistance to stresses, assembly of pili and consequently, pathogenesis. Furthermore, since its crucial importance in the physiology of not only *A*. *baumannii*, but also other pathogens, MltB or other LTs, could be considered a prime target for the development of therapeutics agents to manage or prevent infections.

## 4 EXPERIMENTAL PROCEDURES

### 4.1 Bacterial strains, plasmids and growth media

Strains and plasmids used in this study are listed in Supporting Information Table S6. Bacteria were cultured in Lysogeny Broth (LB) at 37°C. Bacteria were also cultured in M9 minimal medium supplemented with 0.4% glucose and 0.2% casamino acids. Antibiotics and reagents were added as required at the following concentrations: kanamycin, 50 µg ml^–1^; ampicillin, 100 µg ml^–1^; zeocin, 10 µg ml^–1^ (*E*. *coli*) and 200 µg ml^–1^ (*A*. *baumannii*), amikacin, 10 µg ml^–1^; diaminopimelic acid (DAP), 50 µg ml^–1 ^and sucrose, 10% wt/vol.

### 4.2 *Construction of non‐polar mutants,* *transposon library and complemented strain*

Primers used in this study are listed in Supporting Information Table S7. Non‐polar mutants were generated using homologous recombination (Aranda et al., 2010) and allelic exchange (Donnenberg and Kaper, 1991). Homologous recombination was used to delete the *AB57*_*0288* gene (conferring kanamycin resistance). Briefly, the *AB57*_*0288* gene, flanked by ~1 Kb, was PCR‐amplified and cloned into the plasmid pSU2719. Then, a recombineering approach (Yu et al., 2000) was used to replace the *AB57*_*0288* gene with the *sh_ble *cassette flanked with the FRT sites from plasmid pKD_zeo. Then, the mutated fragment was PCR‐amplified, with the 1 Kb flanking region, and electroporated into AB0057 as described by Aranda *et al*. (2010). Following confirmation of the homologous recombination, the *sh*_*ble *cassette was excised using a *Km*‐modified version of pAT03 (Tucker et al., 2014), pAT03_Km. The AB0057 ∆*AB57_0288 *strain was considered as the WT strain in this study and named AB0057^Km ^(WT).

Random Tn*5* transposon insertion mutants were generated in *A. baumannii* strain AB0057^Km^ (WT). Briefly, EZ‐Tn5 transposome (Epicentre) complexes were electroporated into AB0057^Km^ (WT) according to Jacobs *et al*. (2014). To increase transformation efficiency, the TypeOne™ Restriction Inhibitor (Epicentre) was added to the electroporation mixture. Based on the size of the genome (4.05 Mb), 34,000 transposon insertion mutants were required to achieve 99.99 % genome saturation coverage confidence (Zilsel et al., 1992). In total, 49,628 transposon mutants were generated and archived in pools of 5,000 mutants.

Generation of in‐frame markerless mutants was achieved by allelic exchange using a modified version of pCVD442 plasmid (Donnenberg and Kaper, 1991). Briefly, the 5ʹ end of the gene to be deleted possessed at least 1 Kb including the initiation codon and a 6‐nt restriction site, while the 3ʹ region consisted of at least 1 Kb including the last 7 codons and a 6‐nt restriction site. The 5ʹ and 3ʹ regions were cloned into pCVD442_MCS_Amk, which results in the in‐frame deletion of the internal region of the gene of interest. The construct was transformed into the donor strain MGN617, and was transferred to the AB0057^Km^ (WT) strain by conjugation. Transconjugants were selected on LB agar containing amikacin. Individual colonies were cultured 2 hr in LB broth, diluted and spread on LB agar plates containing 10% (wt/vol) sucrose to select the second recombination event. Sucrose‐resistant and amikacin‐sensitive isolates were screened by PCR to confirm deletion of the gene of interest.

Complementation of the *mltB* deletion was achieved by cloning the *mrdB*‐*mltB* operon, including 183 nt upstream of *mrdB*, in the pABBR_Km plasmid.

### 4.3 Mouse infection experiments

All procedures involving the use of mice were performed in strict accordance with the recommendations in the Guide for the Care and Use of Laboratory Animals (8^th^ edition) and were approved by the University Committee on Use and Care of Animals at the University of Michigan (PRO00007111).

Mice were anesthetized with a weight‐appropriate dose (0.1 ml for a mouse weighing 20 gm) of ketamine/xylazine (80–120 mg kg^–1^ ketamine and 5–10 mg kg^–1^ xylazine) by IP injection (model of pneumonia). Mice were euthanized by inhalant anesthetic overdose followed by vital organ removal. All infections performed in this study were mono‐infection. Neutrophils were depleted by intraperitoneal injection of 500 µg of rat anti‐mouse monoclonal antibody (MAb) RB6‐8C5 (RB6) (BioXCell) 24 hpi (Conlan and North, 1994; van Faassen et al., 2007).

For the murine model of bacteremia, infections were performed as described previously (Smith et al., 2010), in which female CBA/J mice aged from 6‐ to 8‐week‐old were inoculated via tail vein injection with 10^7^ CFU. At 24 hpi, mice were euthanized and the spleen, liver and kidneys were aseptically removed, homogenized, diluted and plated on LB‐agar plates to determine the colonization level in these organs.

For the murine model of pneumonia, infections were performed as described elsewhere (Jacobs et al., 2010) with slight modifications. Briefly, female CBA/J mice aged from 6‐ to 8‐week‐old were anesthetized with ketamine/xylazine and pneumonia was induced by intranasal inoculation of 10^7^ CFU suspended in a volume of 20 µl of PBS (10 µl per nostril). At 24 hpi, mice were euthanized and lungs were aseptically removed, homogenized, diluted and plated onto LB agar plates to determine the bacterial burden.

### 4.4 *In vivo screen for *A. baumannii *fitness factors*

Mice were inoculated with transposon library pools as described above. Preparation of the input (inoculum) and output (24 hpi) pools were prepared as described by Anderson *et al*. (2017). Pools of 10,000 mutants (5 pools total; 50,000 mutants) were used to infect four mice each (20 mice total). Two aliquots of 1 ml of each inoculum suspension (input) were collected by centrifugation and stored at −80°C for subsequent isolation of genomic DNA.

### 4.5 *Illumina* *sequencing*

Illumina sequencing was performed as described by Subaschandrabose *et al*. (Subashchandrabose et al., 2013; Subashchandrabose et al., 2016). Briefly, genomic DNA from the input (5 pools of 2 inocula each) and the output (infected spleens, 5 pools of 4 mice each) was isolated by phenol/chloroform/isoamyl alcohol extraction. Genomic DNA (5 µg) was sheared to yield fragments of ≈300 bp (Covaris). Illumina TruSeq adapters were ligated to DNA fragments. Transposon‐gDNA junctions were enriched by PCR using the Tn‐specific primer and the TruSeq Indexed adapter_barcode primers (Supporting Information Table S7). Twenty‐five ng of the TruSeq libraries were used as template for 28 cycles of amplification. Amplicons were further processed for Illumina sequencing according to manufacturer’s recommendations and sequenced, using the Tn‐specific primer, on an Illumina HiSeq 2000 sequencer using the 50‐nucleotide single‐end read cycle. Libraries from input and output samples were sequenced on the same lane, in triplicate. Libraries preparation and sequencing were performed at the University of Michigan DNA core facility.

### 4.6 Mapping of transposon insertion sites

Reads from the input and output libraries starting with AGACAG, corresponding to the end of the transposon, and having more than 15 bp, were aligned to the genome of *A. baumannii* AB0057 (NCBI accession no. NC_011586.1) using the short‐read aligner BOWTIE (Langmead et al., 2009). One nucleotide mismatch was allowed during mapping to the chromosome. Fitness factors were identified by comparing the number of reads that map to a given chromosomal location in the input and output libraries based on the statistical cutoff of fold‐change >2.0 and adjusted *p* < 0.01.

### 4.7 *Resistance of *A. baumannii* to human serum, polymyxin B, acid, oxidative stress and osmotic shock*

Growth of *A. baumannii* in human serum was performed as previously described (Lamarche et al., 2005; Crepin et al., 2012). Briefly, bacteria were cultured overnight in LB broth at 37°C. Bacterial cultures were resuspended 1:100 in fresh medium and grown to mid‐log growth phase (OD_600 _= 0.6). Bacteria were washed with PBS and 10^7^ CFU ml^–1^ were incubated either with 90% heat‐inactivated or 90% normal human serum (Innovative Research). Suspensions were incubated at 37°C and viable cell counts were determined at 0, 1, 2 and 3 hr post‐incubation on LB agar plates.

Resistance to polymyxin B was assessed as described by Crepin *et al*. (2012) with slight modifications. Briefly, bacteria were cultured as described above and 10^7^ CFU ml^‐1^ were incubated with 1 µg ml^‐1 ^of polymixin B for 60 min. The number of bacteria that survived the treatment was determined by CFU enumeration on LB agar.

Resistance to acid was performed as described by Lamarche *et al*. (2005). Bacteria were cultured to mid‐log phase of growth as described above and 10^7^ CFU ml^‐1^ were resuspended in either LB (LB‐pH7) or 100 mM MES‐buffered LB (LB‐pH5). Percent survival at 1 hpi was calculated by dividing the number of CFU recovered from LB‐pH5 by the number of CFU recovered from LB‐pH7.

Resistance to oxidative stress was assessed by culturing bacteria as described above and by mixing 10^7^ CFU ml^–1^ to LB supplemented with 2.5 mM H_2_O_2_. At 30 min post‐inoculation, viable cell counts were determined and percent survival was calculated by dividing the number of CFU recovered by the number of CFU at time 0.

Resistance to osmotic shock was measured as described previously (Lamers et al., 2015). Bacteria were cultured as described above and 10^7^ CFU ml^‐1^l were mixed with either LB containing either 8.55 mM (low salt) or 2.5 mM (high salt) NaCl. The number of cells that survive the treatment was determined by CFU enumeration onto LB agar plate.

The growth of A. *baumannii *in the presence of defined concentrations of NaCl (0, 100, 250, 500 and 750 mM) was measured in LB medium. Strains were cultured overnight in LB without NaCl, washed once in PBS and the OD_600 _was adjusted to 0.1 in medium with the corresponding NaCl concentration. Growth was measured by OD_600_ determination every 30 min with a BioScreen C Analyzer at 37°C with continuous shaking.

### 4.8 Quantitative RT‐PCR

Strains were cultured as described above and RNA was extracted using TRIzol reagent (Thermo Fisher Scientific) according to the manufacturer’s recommendations. RNA samples were submitted to a rigorous DNase treatment using Turbo DNA‐free (Ambion) to remove any DNA contamination. The iScript cDNA synthesis kit and the SsoFast Evagreen Supermix kit (Bio‐Rad) were used for qRT‐PCR analysis according to the manufacturer’s instructions. The *gyrB* gene was used as a housekeeping control (Anderson et al., 2017). Gene expression was calculated using the 2^−ΔΔ^ *^CT^* method (Livak and Schmittgen, 2001). Genes with a fold‐change above or below the defined threshold of two were considered as differentially expressed. Primers used for qRT‐PCR analysis are listed in Supporting Information Table S7.

### 4.9 *Measurement of membrane permeability using Syto* *9 and propidium* *iodide dyes*

Bacteria cultured to mid‐log phase of growth were exposed to BacLight viability dyes propidium iodide and Syto 9 (Thermo Fisher Scientific). Three fields per slide were captured per biological replicate and ~300 cells per biological replicate were counted. Percent of *A*. *baumannii* bacteria positive for propidium iodide staining, an indication of a permeable membrane, was calculated by dividing the propidium iodide‐positive bacteria by the total number of bacteria. Images were captured with a Zeiss Axioplan 2 epifluorescence microscope equipped with a 100× Plan‐Neofluor objective with a numerical aperture of 1.3. Images were analyzed and processed with FIJI (Schindelin et al., 2012).

### 4.10 Biofilm formation

Biofilm formation was measured as previously described (Subashchandrabose et al., 2013) with slight modifications. Briefly, bacteria were cultured in LB overnight, washed twice in PBS and normalized to an OD_600_ of 0.05 in 1 ml of fresh LB. Cultures were incubated in polystyrene culture tubes at 30°C for 24 hr under static conditions. Supernatants were aspirated and tubes were washed three times with water and stained with 1.5 ml of 1.0% crystal violet solution for 10 min. Biofilm‐bound crystal violet was dissolved in 2 ml of 33% acetic acid and absorbance was measured at 540 nm.

### 4.11 Adhesion assay

The adenocarcinomic human alveolar basal epithelial cells A549 (American Type Culture Collection ATCC^®^ CCL‐185™) were cultured to confluence in 24‐well plates in Kaighn’s Modification of Ham’s F‐12 Medium (ATCC^®^ 30‐2004™) supplemented with 10% heat‐inactivated fetal bovine serum (FBS) at 37°C and 5% CO_2_. Bacterial strains were cultured overnight in LB, washed twice with PBS and adjusted to 10^7^ CFU ml^–1^ in Kaighn’s Modification of Ham’s F‐12 Medium supplemented with 10% heat‐inactivated FBS. The mixture was added to each well containing 10^6 ^A549 cells (MOI of 10). Bacterium‐host cell contact was enhanced by a 5‐min centrifugation at 600 × *g*. At 2 hr post‐incubation, cells were washed 3 times with DPBS (removing the non‐adherent bacteria), lysed with 0.25 % Triton X‐100 for 5 min and then, serially diluted for CFU enumeration. No difference in survival rate between strains were observed at 5 min post‐incubation with Triton X‐100 (data not shown). Quantification of cell‐associated bacteria was performed as previously described (Crepin et al., 2012).

### 4.12 Transmission electron microscopy

Transmission electron microscopy was performed as described previously (Subashchandrabose et al., 2013) with slight modifications. Briefly, bacterial strains were cultured overnight in LB, washed twice in PBS and adjusted in PBS to an OD_600_ of 1.0. Ten µl were pipetted onto Formvar/Carbon 300 Mesh Copper Grids (Ted Pella). Bacteria were allowed to adhere to the grids for 5 min, then excess liquid medium was wicked off with filter paper. Grids were washed once with 10 µl of deionized water, then stained for 5 min with 10 µl of 1% phosphotungstic acid (pH 6.8). Excess stain was removed; grids were washed with 10 µl of deionized water and dried. Grids were visualized using a JEOL JSM 1400 plus transmission electron microscope at Microscopy & Image Analysis Laboratory of the University of Michigan.

### 4.13 Peptidoglycan analysis

The PG was extracted and analyzed according to B. Glauner (1988). Briefly, *A*. *baumannii* strains were grown in LB or 50% heat‐inactivated human serum (Innovative Research) to ~10^8^ CFU ml^–1^. Cells were then collected by centrifugation, resuspended in 6 ml ice‐cold water and lysed by drop wise addition to 6 ml boiling 8% SDS. The PG was purified and digested with the muramidase cellosyl (Hoechst, Frankfurtam Main, Germany) to release the muropeptides, which were reduced by sodium borohydride, and separated on a Prontosil 120‐3‐6C18 AQ reversed phase column (Bischoff, Leonberg, Germany). The eluted muropeptides were detected by their absorbance at 205 nm.

### 4.14 Statistical analyses

All data were analyzed using the GraphPad Prism 7 software program. A Mann–Whitney test was used to determine statistical significance for mono‐infection experiments. All other statistical analyses were determined by the Student’s *t*‐test and either one‐ or two‐way analysis of variance (ANOVA) with Tukey’s or Sidak’s multiple comparison test.

## Author contributions

S.C. and H.L.T.M. designed the experiments. S.C., E.N.O., K.P., S.N.S and S.D.H. performed the experiments. S.C., E.N.O. and H.L.T.M. analyzed the data. H.L.T.M. and W.V. contributed funding and resources. S.C. and H.L.T.M. wrote the manuscript. All authors reviewed, edited and approved the manuscript.
