# Supplementary material for: The lytic transglycosylase MltB connects membrane homeostasis and in vivo fitness of Acinetobacter baumannii
Source: Mol Microbiol. 2018 Sep 28;109(6):745–62. doi: 10.1111/mmi.14000 (PMC6185781; doi:10.1111/mmi.14000)
Supplement: Supplementary file 1 [file MMI-109-745-s001.pdf]

SUPPLEMENTARY INFORMATION

The lytic transglycosylase MltB connects membrane homeostasis and *in vivo* fitness of *Acinetobacter baumannii*

Sébastien Crépin, Elizabeth N. Ottosen, Katharina Peters, Sara N. Smith, Stephanie D. Himpsl, Waldemar Vollmer, and Harry L.T. Mobley

CONTENTS

Fig. S1. Validation that Δ*km* mutant colonizes the bloodstream as well as AB0057.

Fig. S2. Colonization of the neutropenic mouse model of bloodstream infection by the transposon pools.

Fig. S3. Capsule production in strain AB0057.

Fig. S4. Stress resistance of the *mltB* mutant in strain AB5075.

Fig. S5. Capsule production in strain AB5075.

Fig. S6. Growth of strain AB0057 in presence of NaCl.

Fig. S7. Growth of strain AB5075 in presence of NaCl.

Fig. S8. Adhesion of the *mltB* mutant of strain AB5075.

Fig. S9. Adhesion to polystyrene surface.

Table S1. Random distribution of the transposon across the chromosome.

Table S2. Candidate fitness factors.

Table S3. *A. baumannii* lytic transglycosylases and their closest homolog.

Table S4. *A. baumannii* peptidoglycan composition.

Table S5. Comparative analysis of candidate fitness factors with other Tn-seq analyses.

Table S6. Strains and plasmids used in the study.

Table S7. Primers and oligonucleotides.

SUPPORTING INFORMATION FIGURES S1-9.

Fig. S1. Validation that ∆*km* mutant colonizes the bloodstream as well as AB0057.

CBA/J mice were infected with 10^7 CFU of either strain AB0057 or the *km* mutant ($AB0057^{Km}$) by tail vein injection. At 24 hpi, mice were sacrificed, spleen and liver were harvested, and the bacterial burden was determined by CFU enumeration on LB agar. Bacterial numbers are presented as the log_{10} CFU g^{-1} of tissue. Each data point represents a sample from an individual mouse, and horizontal bars indicate the median values. Statistical significance was calculated by the Mann-Whitney test. NS, not significant.

Fig. S2. Colonization of the neutropenic mouse model of bloodstream infection by the transposon pools.

Recovery of the transposon mutants (pools #1 to 5) during bloodstream infection. CBA/J mice were infected with 10^7 CFU of either wild type strain $AB0057^{Km}$ (57) or the transposon pools (Tn pool #1 to #5) by tail vein injection. At 24 hpi, mice were sacrificed, spleen and liver were harvested, and the bacterial burden was determined by CFU enumeration on LB agar. Bacterial numbers are presented as the log_{10} CFU g^{-1} of tissue. Each data point represents a sample from an individual mouse, and horizontal bars indicate the median values.

Abbreviation: 57, WT ($AB0057^{Km}$); Tn, transposon.

Fig. S3. Capsule production in strain AB0057.

A. Maneval's staining of bacteria grown 24 h on LB-agar plate. Images are representative of three independent experiments.

B. Mucoviscosity measurement. Mucoviscosity was used as an indicator of capsule production by measuring optical density (OD_{600}) of the supernatant after centrifugation for 5 min at 1,000 × *g* from a starting turbidity of 1.0.

All results are the mean values and standard deviations of three independent

experiments. Statistical significance was calculated by One-way Anova and the Tukey's multiple comparisons test *, *P*<0.05; ***, *P*<0.0005; ****, *P*<0.0001.

Abbreviation: 57, WT (AB0057^{Km}); compl.: complemented (pABBR_Km-*mrdB*-*mltB*).

Fig. S4. Stress resistance of the *mltB* mutant in strain AB5075.

Resistance to stresses was determined by incubating 10^7 CFU ml^{-1} of the AB5075 (75) and its isogenic *mltB* mutant to different stressors.

A. Survival in 90% human active serum (HS) and growth in 90% heat-inactivated human serum (HI). The number of surviving CFUs was quantified by CFU enumeration on LB agar every hour. For ease of reading, standard deviations were removed.

B. Survival in the presence of 1 µg ml^{-1} of polymyxin B. The number of CFUs recovered at 60 min (T_{60}) was determined by CFU enumeration on LB agar and compared to time 0 (T_0).

C. Survival to oxidative stress (2.5 mM H_2O_2). The percent survival to 2.5 mM H_2O_2 was determined by dividing the number of CFU recovered at 30 min (T_{30}) post-inoculation by the number of CFUs at time 0 (T_0).

D. Survival in an acidic environment. The percent survival to acidic environment was determined by dividing the number of CFU recovered at 60 min ($T_{\mathbf{60}}$) post-inoculation in LB-pH5 by the number of CFUs recovered in LB-pH7.

E. Survival of osmotic shock. The number of CFUs recovered in LB containing 8.85 mM or 2.5 M NaCl at 2 h (T_2) post-inoculation was determined by CFU enumeration on LB agar.

Results are the mean values and standard deviations of three biological experiments. Statistical significance was calculated by the Student's *t*-test (*, *P*<0.05; NS: not significant).

Fig. S5. Capsule production in strain AB5075.

A. Maneval's staining of bacteria grown 24 h on LB-agar plate. Images are representative of three independent experiments.

B. Mucoviscosity measurment. Mucoviscosity was used as an indicator of capsule production by measuring optical density (OD_{600}) of the supernatant after centrifugation for 5 min at 1,000 × *g* from a starting turbidity of 1.0.

All results are the mean values and standard deviations of three independent experiments. Statistical significance was calculated by the Student's *t*-test (***, *P*<0.001). Abbreviation: 75, WT (AB5075).

Fig. S6. Growth of strain AB0057 in presence of NaCl.

A-D. The WT strain (57) and its derivative strains were grown in presence of different concentration of NaCl. (A) 100 mM. (B) 250 mM. (C) 500 mM. (D)D 750 mM. Results are the mean values and standard deviations of three biological experiments. For ease of reading, standard deviations were removed from graphs. Statistical significance was calculated by the two-way ANOVA and Tukey's multiple comparisons test (****, *P*<0.0001).

Abbreviations: 57, WT ($AB0057^{Km}$); eV, empty vector (pABBR_Km); compl.: complemented (pABBR_Km-*mrdB*-*mltB*).

Fig. S7. Growth of strain AB5075 in presence of NaCl.

The WT strain AB5075 (75) and its isogenic *mltB* mutant were grown in presence of different concentration of NaCl. (A) 500 mM. (B) 750 mM. (**B**) Results are the mean values and standard deviations of three biological experiments. For ease of reading, standard deviations were removed from graphs. Statistical significance was calculated by the two-way ANOVA. No differences were observed between the AB5075 and its isogenic *mltB* mutant.

Fig. S8. Adhesion of the *mltB* mutant of strain AB5075.

A. Biofilm formation in LB at 30°C for 24 h under static conditions. Crystal violet binding assay was used to monitor biofilm formation.

B. Adherence onto A549 lung epithelial cells.

Results are the mean values and standard deviations of three biological experiments. Statistical significance was calculated by the Student's *t*-test (*, $P<0.05$).

Abbreviation: 75: AB5075.

Fig. S9. Adhesion to polystyrene surface.

A. Adhesion of AB0057 to and its derivative strains to polystyrene.

B. Adhesion of AB5075 and its isogenic *mltB* mutant to polystyrene.

Results are the mean values and standard deviations of three biological experiments. Statistical significance was calculated by the Student's *t*-test (NS: Not significant).

Abbreviation: 57: WT ($AB0057^{Km}$); eV: empty vector (pABBR_Km); compl.: complemented (pABBR_Km-*mrdB*-*mltB*); 75: AB5075.

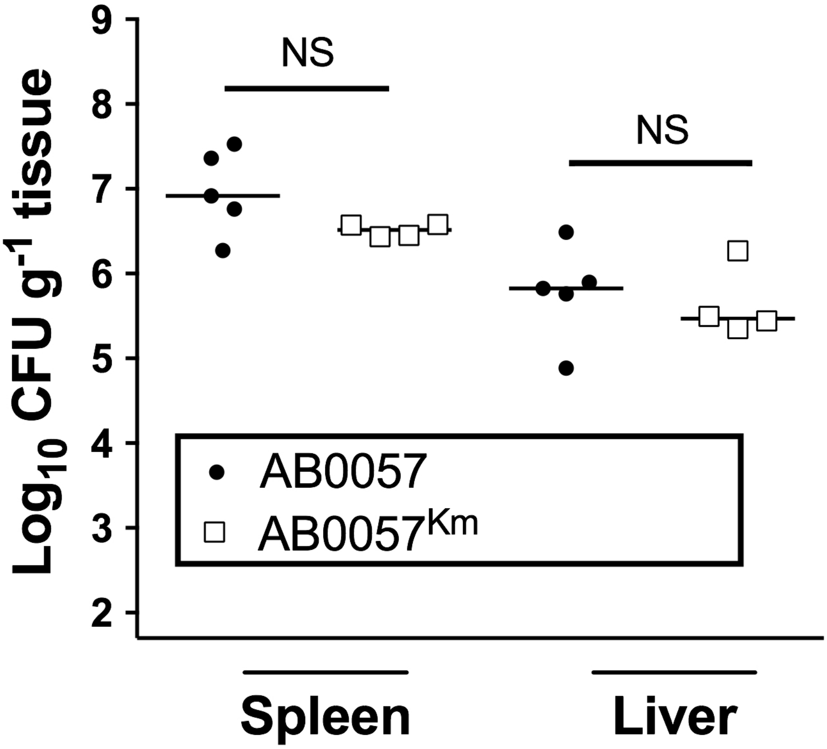

Log10 CFU g-1 tissue
9
8
7
6
5
4
3
2
NS
NS
AB0057
AB0057Km
Spleen
Liver

Fig. S1.

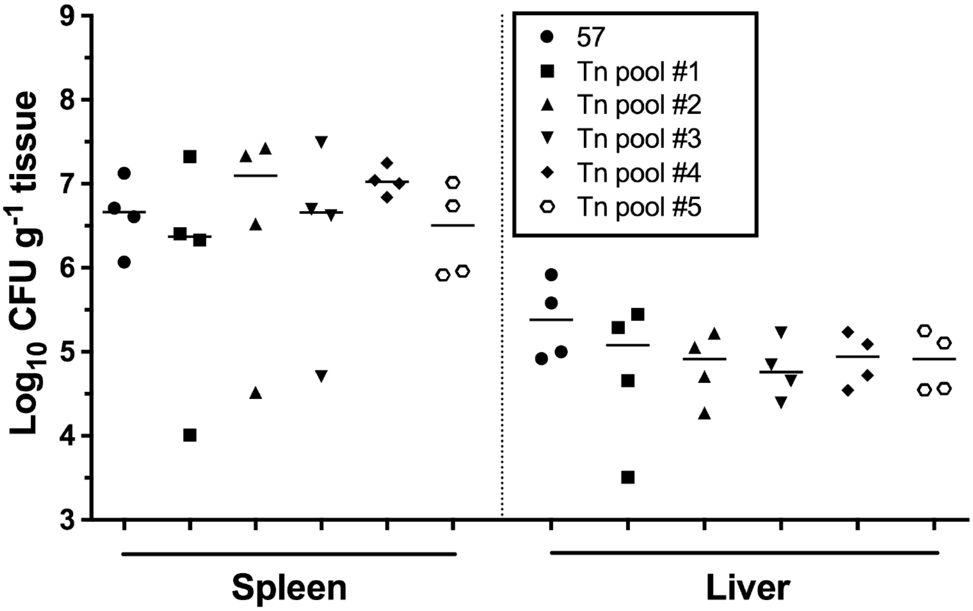

9
8
7
6
5
4
3
Log10 CFU g-1 tissue
57
Tn pool #1
Tn pool #2
Tn pool #3
Tn pool #4
Tn pool #5
Spleen
Liver

Fig. S2.

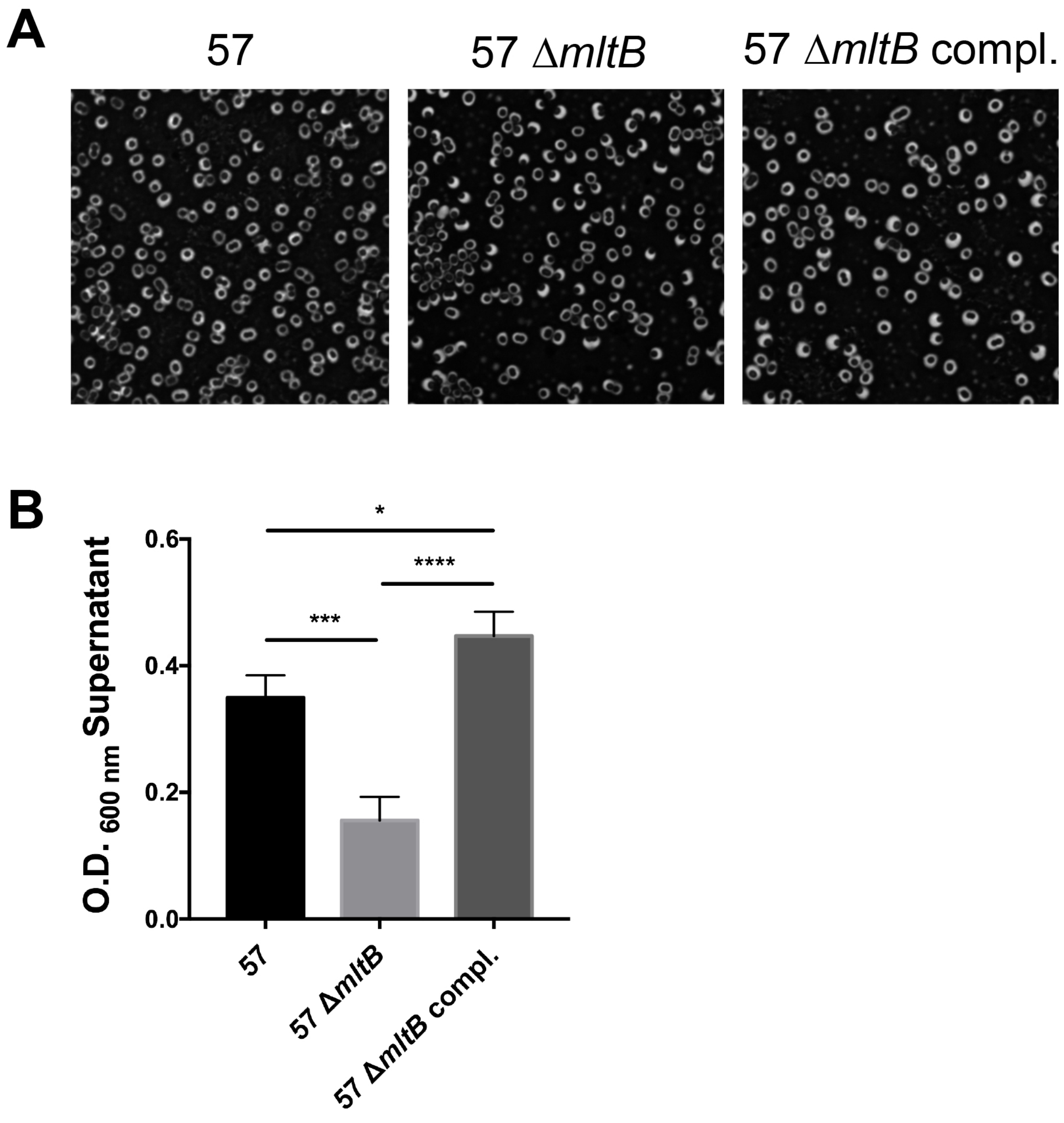
A
57
57 ΔmltB
57 ΔmltB compl.
B
O.D. 600 nm Supernatant
0.6
0.4
0.2
0.0
*
****
***
57
57 ΔmltB
57 ΔmltB compl.

Fig. S3.

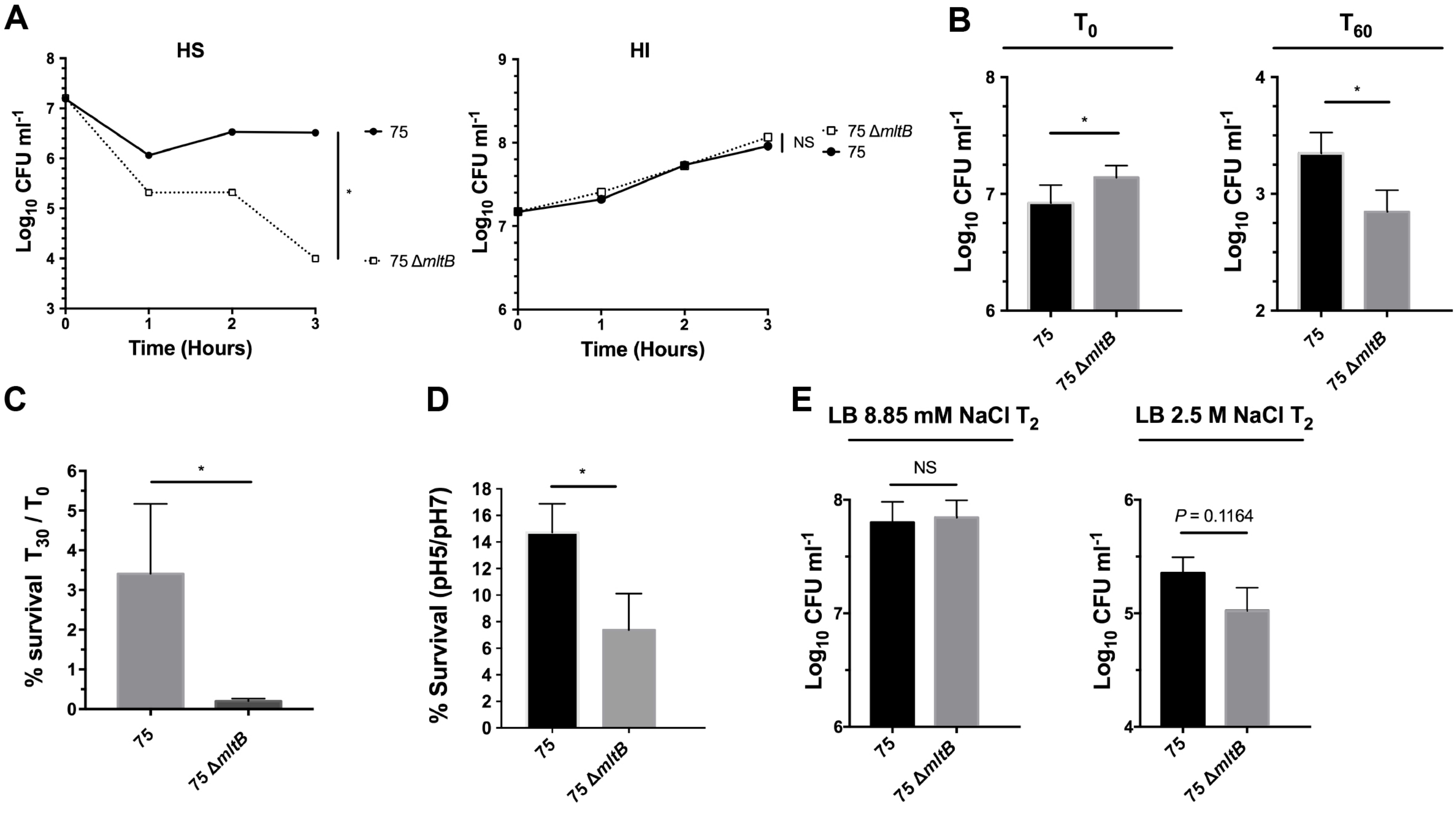
A
HS
Log10 CFU ml-1
Time (Hours)
75
75 ΔmltB
*
HI
NS
B
T0
T60
C
% survival T30 / T0
D
% Survival (pH5/pH7)
E
LB 8.85 mM NaCl T2
LB 2.5 M NaCl T2
P = 0.1164

Fig. S4.

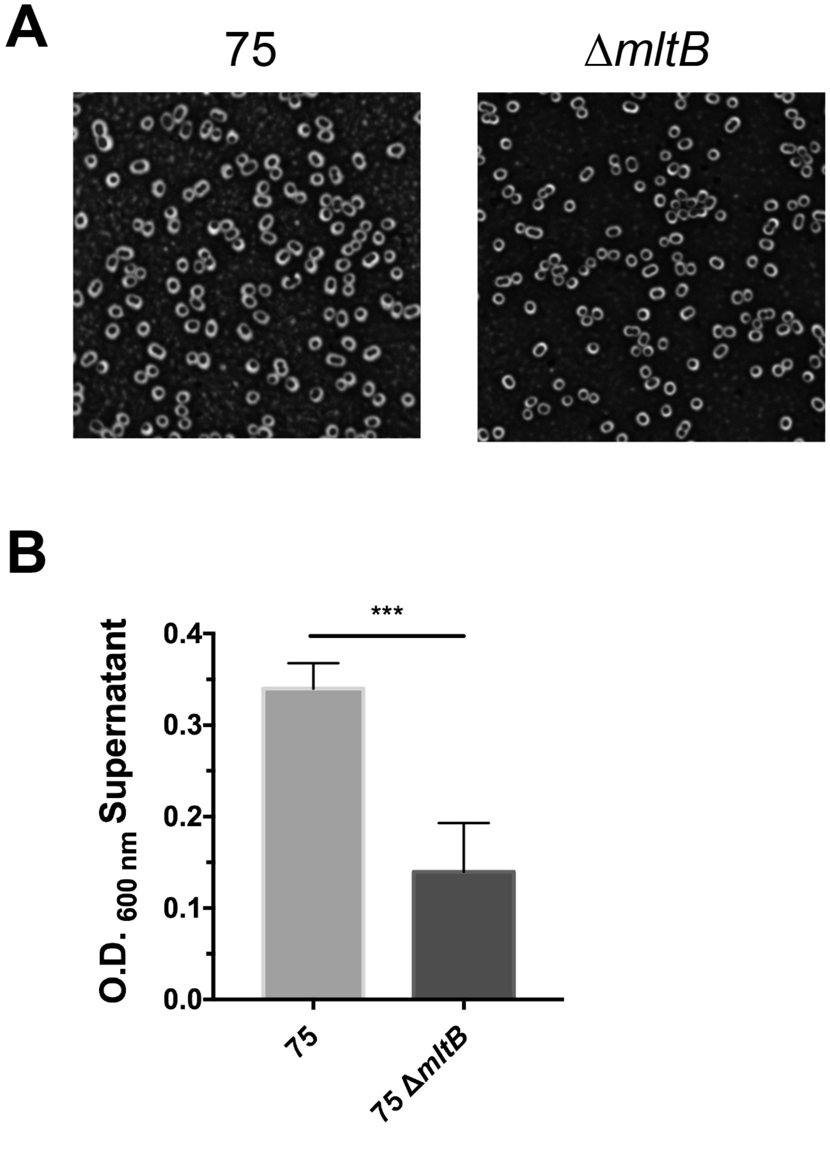

Fig. S5.

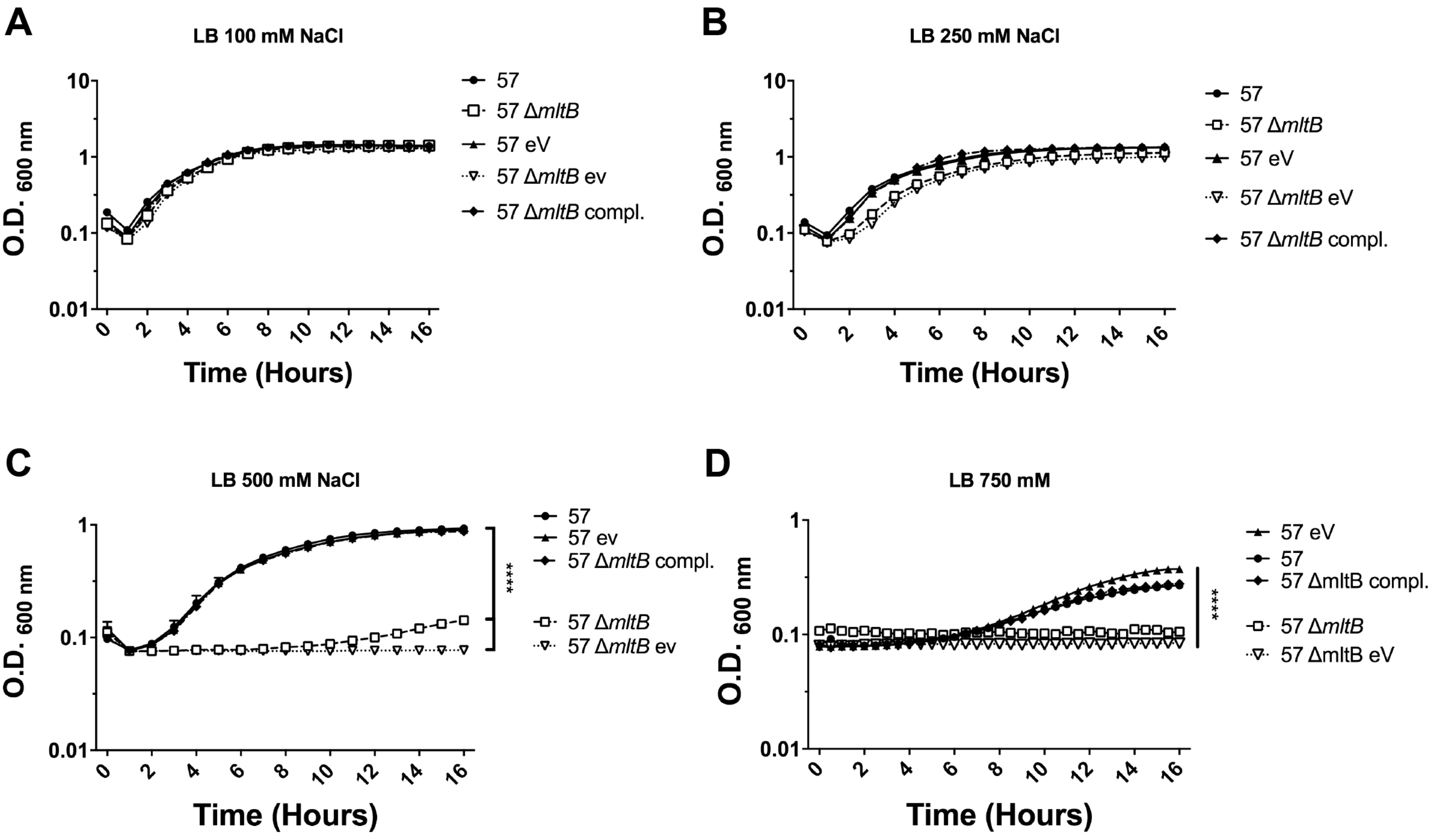
A
LB 100 mM NaCl
O.D. 600 nm
10
1
0.1
0.01
0
2
4
6
8
10
12
14
16
Time (Hours)
57
57 ΔmltB
57 eV
57 ΔmltB ev
57 ΔmltB compl.
B
LB 250 mM NaCl
O.D. 600 nm
10
1
0.1
0.01
0
2
4
6
8
10
12
14
16
Time (Hours)
57
57 ΔmltB
57 eV
57 ΔmltB eV
57 ΔmltB compl.
C
LB 500 mM NaCl
O.D. 600 nm
1
0.1
0.01
0
2
4
6
8
10
12
14
16
Time (Hours)
****
57
57 ev
57 ΔmltB compl.
57 ΔmltB
57 ΔmltB ev
D
LB 750 mM
O.D. 600 nm
1
0.1
0.01
0
2
4
6
8
10
12
14
16
Time (Hours)
****
57 eV
57
57 ΔmltB compl.
57 ΔmltB
57 ΔmltB eV

Fig. S6.

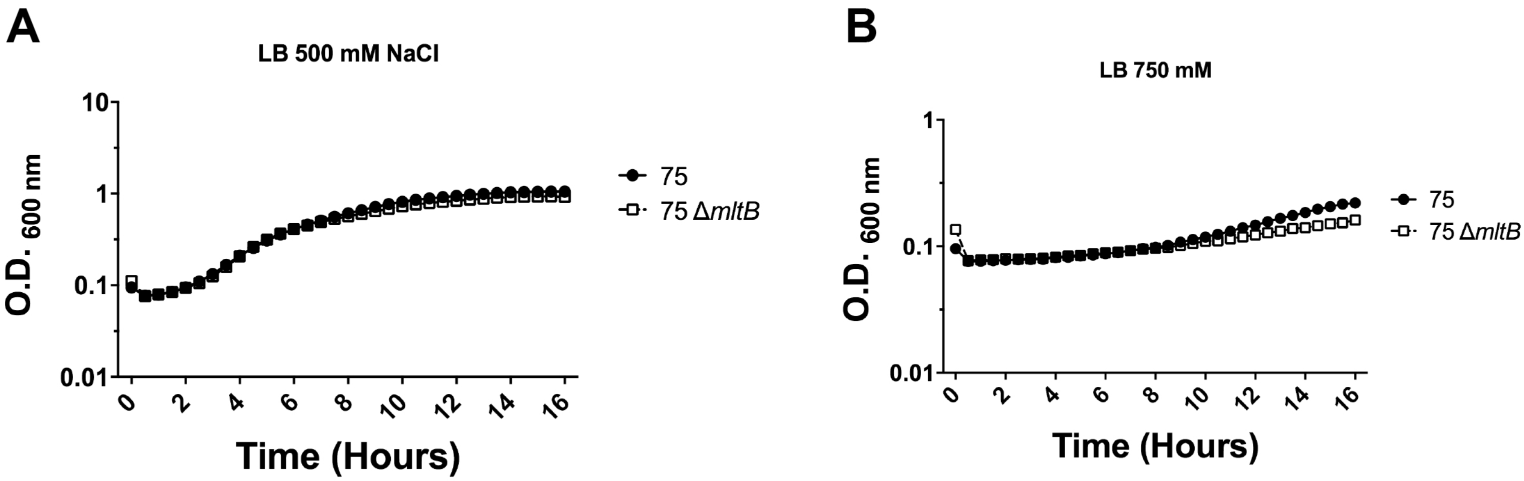
A
LB 500 mM NaCl
10
1
0.1
0.01
O.D. 600 nm
0
2
4
6
8
10
12
14
16
Time (Hours)
75
75 ΔmltB
B
LB 750 mM
1
0.1
0.01
O.D. 600 nm
0
2
4
6
8
10
12
14
16
Time (Hours)
75
75 ΔmltB

Fig. S7.

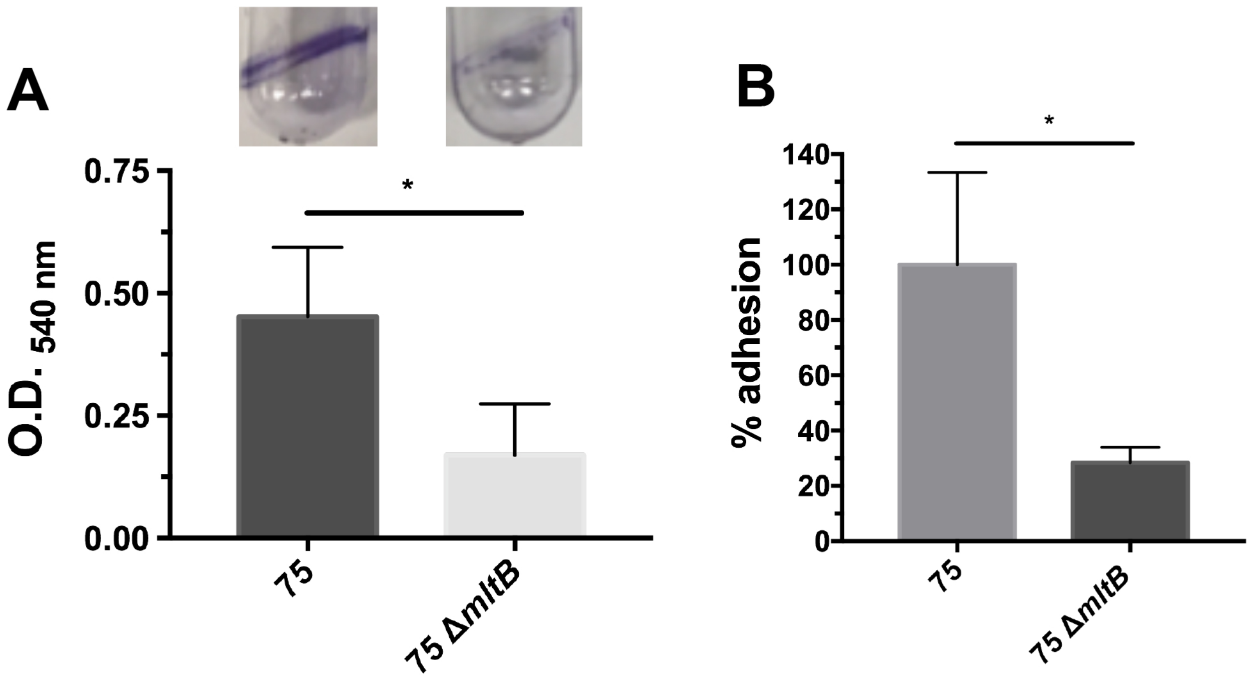
A
B
0.75
0.50
0.25
0.00
O.D. 540 nm
*
75
75 ΔmltB
140
120
100
80
60
40
20
0
% adhesion

Fig. S8.

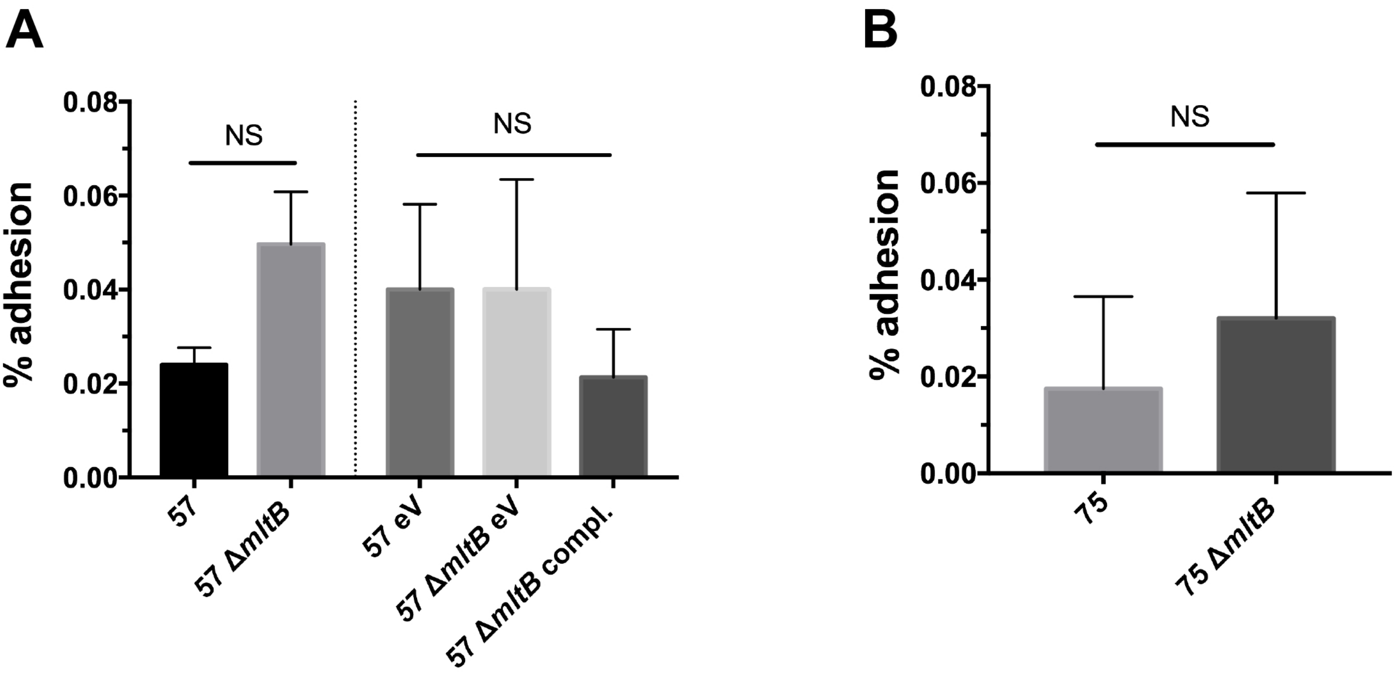
A
B
% adhesion
0.08
0.06
0.04
0.02
0.00
NS
57
57 ΔmltB
57 eV
57 ΔmltB eV
57 ΔmltB compl.
75
75 ΔmltB

Fig. S9.

Table S1. **Random distribution of the *Tn5* across the chromosome.**

Mutant #	Integration site	Mutant #	Integration site
1	*insB3*	11	*AB57_0054*
2	*macB*	12	*AB57_0519*
3	*tonB*	13	*AB57_3074*
4	*AB57_1775*	14	Intergenic region between *pilG* and *AB57_3316*
5	*esvE1*	15	*AB57_0369*
6	*AB57_0857*	16	*AB57_2259*
7	*aph1*	17	*AB57_3071*
8	*AB57_3085*	18	*AB57_3092*
9	*AB57_3329*	19	*AB57_0291*
10	*AB57_1502*	20	*AB57_0091*

Sequencing approach was used to identify the integration site of selected *Tn5* mutants.

Table S2. Candidate fitness factors during bloodstream infection.

Locus tag	Locus tag	Function	Log_2 Fitness defect in spleen	*P* value
AB57_RS16925	AB57_3427	sulfurtransferase	-10.82	0.0096
AB57_RS03515	AB57_0688	2-amino-4-hydroxy-6-hydroxymethyldihydropteridine pyrophosphokinase	-10.31	0.0078
AB57_RS12865	AB57_2595	LuxR family transcriptional regulator	-10.18	0.0003
AB57_RS16615	AB57_3365	hypothetical protein	-10.11	0.0034
AB57_RS16225	AB57_3288	hypothetical protein	-9.66	0.0091
AB57_RS00885	AB57_0173	hypothetical protein	-9.63	0.0052
AB57_RS17590	AB57_3551	toluene tolerance protein	-9.60	0.0062
AB57_RS16280	AB57_3300	hypothetical protein	-9.57	0.0001
AB57_RS09270	AB57_1860	MULTISPECIES: DNA-binding protein HU-beta	-9.52	0.0044
AB57_RS05825	AB57_1154	hypothetical protein	-9.48	0.0075
AB57_RS14300	AB57_2881	diadenosine tetraphosphatase	-9.47	0.0031
AB57_RS18240	AB57_3680	acetyltransferase	-9.37	0.0062
AB57_RS13640	AB57_2749	lytic transglycosylase	-9.36	0.0000
AB57_RS14220	AB57_2865	NUDIX hydrolase	-9.24	0.0022
AB57_RS02755	AB57_0530	MULTISPECIES: 50S ribosomal protein L33	-9.24	0.0001
AB57_RS02760	AB57_0531	MULTISPECIES: 50S ribosomal protein L28	-9.24	0.0001
AB57_RS02405	AB57_0459	hypothetical protein	-9.21	0.0052
AB57_RS01760	AB57_0336	glutamyl-Q tRNA(Asp) ligase	-9.17	0.0000
AB57_RS19005	AB57_3836	phosphoserine phosphatase	-9.16	0.0035
AB57_RS12520	AB57_2526	phosphoribosylamine--glycine ligase	-9.15	0.0001
AB57_RS18340	AB57_3700	endonuclease	-9.15	0.0050
AB57_RS18195		hypothetical protein	-9.09	0.0094
AB57_RS13620	AB57_2745	membrane protein	-9.08	0.0000
AB57_RS00880	AB57_0172	PadR family transcriptional regulator	-9.08	0.0094
AB57_RS03540	AB57_0693	hypothetical protein	-9.07	0.0038
AB57_RS08135	AB57_1626	allantoin permease	-9.07	0.0003
AB57_RS19160	AB57_3871	4-hydroxyphenylpyruvate dioxygenase	-9.07	0.0078
AB57_RS00935	AB57_0183	MULTISPECIES: Fur family transcriptional regulator	-9.02	0.0043

Table S2. continued.

Locus tag	Locus tag	Function	Log_2 Fitness defect in spleen	*P* value
AB57_RS04245	AB57_0829	methionine--tRNA ligase	-9.02	0.0012
AB57_RS19435	AB57_3924	membrane protein	-9.00	0.0003
AB57_RS00340	AB57_0065	TetR family transcriptional regulator	-8.97	0.0038
AB57_RS01915	AB57_4828	tRNA-Trp	-8.97	0.0052
AB57_RS11190	AB57_2247	N-acetyl-gamma-glutamyl-phosphate reductase	-8.95	0.0034
AB57_RS03840	AB57_0752	hypothetical protein	-8.95	0.0094
AB57_RS18715	AB57_3777	pyruvate dehydrogenase	-8.92	0.0019
AB57_RS04780	AB57_0935	phosphomannomutase	-8.90	0.0084
AB57_RS14655	AB57_RS14655	ribonuclease 3	-8.90	0.0001
AB57_RS18200	AB57_3672	heme ABC transporter ATP-binding protein	-8.86	0.0091
AB57_RS08130	AB57_1625	MULTISPECIES: AsnC family transcriptional regulator	-8.86	0.0001
AB57_RS19460	AB57_3929	hypothetical protein	-8.85	0.0002
AB57_RS17790	AB57_3593	hypothetical protein	-8.85	0.0001
AB57_RS17115	AB57_3463	TetR family transcriptional regulator	-8.84	0.0016
AB57_RS19230	AB57_3885	dienelactone hydrolase	-8.84	0.0009
AB57_RS05910	AB57_1169	MULTISPECIES: GntR family transcriptional regulator	-8.82	0.0000
AB57_RS16190		rod shape-determining protein MreD	-8.81	0.0058
AB57_RS02165	AB57_0412	preprotein translocase subunit SecG	-8.79	0.0023
AB57_RS00085	AB57_0017	DNA gyrase subunit B	-8.79	0.0049
AB57_RS14295	AB57_2880	2,4-diaminobutyrate decarboxylase	-8.79	0.0013
AB57_RS05665	AB57_1125	cobalt transporter	-8.78	0.0000
AB57_RS00485	AB57_0095	NAD-dependent dehydratase	-8.78	0.0024
AB57_RS14750	AB57_2981	7-carboxy-7-deazaguanine synthase	-8.78	0.0091
AB57_RS00750	AB57_0148	enoyl-CoA hydratase	-8.77	0.0024
AB57_RS00830	AB57_0162	BolA family transcriptional regulator	-8.76	0.0000
AB57_RS02660	AB57_0511	lauroyl acyltransferase	-8.76	0.0046
AB57_RS16810	AB57_3405	LPS biosynthesis protein	-8.75	0.0027
AB57_RS00210	AB57_0043	phosphoribosylaminoimidazole carboxylase	-8.74	0.0037
AB57_RS15740	AB57_3188	hypothetical protein	-8.73	0.0030

Table S2. continued.

Locus tag	Locus tag	Function	Log_2 Fitness defect in spleen	*P* value
AB57_RS17745	AB57_3583	succinylglutamate desuccinylase	-8.72	0.0000
AB57_RS18725		hypothetical protein	-8.71	0.0003
AB57_RS00220	AB57_0045	hypothetical protein	-8.69	0.0008
AB57_RS03490	AB57_0683	GTP pyrophosphokinase	-8.68	0.0095
AB57_RS00030	AB57_0006	RND transporter	-8.67	0.0020
AB57_RS03280	AB57_0637	NAD(P)-binding enzyme	-8.66	0.0009
AB57_RS19190	AB57_3877	hypothetical protein	-8.65	0.0013
AB57_RS00255	AB57_0052	MULTISPECIES: hypothetical protein	-8.62	0.0000
AB57_RS01690	AB57_0322	peptidase S41	-8.61	0.0006
AB57_RS16835	AB57_3410	glutamine-synthetase adenylyltransferase	-8.60	0.0021
AB57_RS00010	AB57_0002	anhydro-N-acetylmuramic acid kinase	-8.60	0.0048
AB57_RS02895	AB57_0558	universal stress protein A	-8.60	0.0008
AB57_RS06935	AB57_1373	membrane protein	-8.58	0.0062
AB57_RS17620	AB57_3557	inositol monophosphatase	-8.58	0.0051
AB57_RS02385	AB57_0454	hypothetical protein	-8.57	0.0030
AB57_RS16370	AB57_3312	chemotaxis protein	-8.56	0.0000
AB57_RS13015	AB57_2622	thiamine biosynthesis protein ThiS	-8.55	0.0000
AB57_RS11540	AB57_2319	ribosome-recycling factor	-8.53	0.0002
AB57_RS02775	AB57_0534	formyltetrahydrofolate deformylase	-8.53	0.0015
AB57_RS11345	AB57_2279	branched-chain amino acid transporter 2 carrier protein BrnQ	-8.53	0.0001
AB57_RS01695	AB57_0323	two-component system response regulator	-8.52	0.0001
AB57_RS04785	AB57_0936	acetylglutamate kinase	-8.52	0.0047
AB57_RS01205	AB57_0236	pyrophosphatase	-8.51	0.0062
AB57_RS00740	AB57_0146	AMP-binding protein	-8.49	0.0000
AB57_RS11305	AB57_2270	hypothetical protein	-8.49	0.0034
AB57_RS18225	AB57_3677	MULTISPECIES: DNA-binding response regulator	-8.48	0.0017
AB57_RS17855	AB57_3607	D-3-phosphoglycerate dehydrogenase	-8.48	0.0027
AB57_RS08750	AB57_1755	DNA-binding protein	-8.48	0.0002
AB57_RS00215	AB57_0044	lytic transglycosylase	-8.47	0.0000

Table S2. continued.

Locus tag	Locus tag	Function	Log$_2$ Fitness defect in spleen	*P* value
AB57_RS00420	AB57_0081	fatty acid desaturase	-8.46	0.0062
AB57_RS09275	AB57_1861	peptidylprolyl isomerase	-8.45	0.0019
AB57_RS11480	AB57_2307	DNA recombination/repair protein RecA	-8.43	0.0044
AB57_RS19235	AB57_3886	hydrolase	-8.42	0.0076
AB57_RS12950		colicin V biosynthesis protein	-8.42	0.0059
AB57_RS01375		MULTISPECIES: transcriptional regulator	-8.41	0.0000
AB57_RS02120	AB57_0403	hypothetical protein	-8.41	0.0000
AB57_RS18840	AB57_3803	5-hydroxyisourate hydrolase	-8.40	0.0031
AB57_RS09090	AB57_1824	hypothetical protein	-8.40	0.0000
AB57_RS16195	AB57_3282	rod shape-determining protein MreC	-8.39	0.0000
AB57_RS15870	AB57_3216	hypothetical protein	-8.39	0.0075
AB57_RS00930	AB57_0182	zinc ABC transporter ATP-binding protein	-8.38	0.0007
AB57_RS00015	AB57_0003	hydrolase	-8.38	0.0034
GenBank:repeat_region:NC_011586:315546:317142			-8.36	0.0030
AB57_RS08335	AB57_1669	hypothetical protein	-8.36	0.0075
AB57_RS13805	AB57_2778	MULTISPECIES: hypothetical protein	-8.35	0.0003
AB57_RS06615	AB57_1310	integrase	-8.35	0.0002
AB57_RS08475	AB57_1697	L-asparaginase	-8.35	0.0032
AB57_RS03635	AB57_0712	tRNA(Ile)-lysidine synthase	-8.34	0.0003
AB57_RS15230	AB57_3083	3\',5\'-cyclic-nucleotide phosphodiesterase	-8.34	0.0000
AB57_RS16760	AB57_3395	phospholipase D	-8.34	0.0000
AB57_RS16360	AB57_3310	hypothetical protein	-8.33	0.0046
AB57_RS02255	AB57_0427	2-octaprenylphenol hydroxylase	-8.33	0.0037
AB57_RS00070	AB57_0014	glutathione ABC transporter ATP-binding protein	-8.32	0.0020
AB57_RS15470	AB57_3133	hypothetical protein	-8.32	0.0003
AB57_RS15265	AB57_3090	methyltransferase	-8.29	0.0001
AB57_RS00415	AB57_0080	oxidoreductase	-8.29	0.0011
AB57_RS00185	AB57_0038	hypothetical protein	-8.29	0.0020

Table S2. continued.

Locus tag	Locus tag	Function	Log$_2$ Fitness defect in spleen	*P* value
AB57_RS02510	AB57_0480	hypothetical protein	-8.28	0.0002
AB57_RS01380	AB57_0271	MULTISPECIES: mercuric reductase	-8.28	0.0027
AB57_RS16720	AB57_3387	DNA-binding response regulator	-8.28	0.0030
AB57_RS17985	AB57_3633	hypothetical protein	-8.28	0.0029
AB57_RS01565	AB57_0297	DNA invertase Pin	-8.27	0.0000
AB57_RS04405	AB57_0863	SMC-Scp complex subunit ScpB	-8.27	0.0050
AB57_RS17585	AB57_3549	MULTISPECIES: toluene-tolerance protein (Ttg2E)	-8.27	0.0000
AB57_RS00840	AB57_0164	chromosome partitioning protein ParA	-8.26	0.0033
AB57_RS05145	AB57_1012	hypothetical protein	-8.26	0.0002
AB57_RS13735	AB57_2767	adenosylhomocysteinase	-8.25	0.0029
AB57_RS01775	AB57_0339	NUDIX hydrolase	-8.25	0.0001
AB57_RS00165	AB57_0034	hypothetical protein	-8.25	0.0012
GenBank:repeat_region:NC_011586:2653756:2653874			-8.24	0.0030
AB57_RS04255	AB57_0831	ATP-binding protein	-8.24	0.0001
AB57_RS03525	AB57_0690	pantothenate synthetase	-8.24	0.0009
AB57_RS02085	AB57_0396	transcriptional regulator	-8.24	0.0051
AB57_RS01390		MULTISPECIES: hypothetical protein	-8.24	0.0044
AB57_RS15310	AB57_3096	MULTISPECIES: ATP-dependent metalloprotease	-8.23	0.0094
AB57_RS15620	AB57_3163	hypothetical protein	-8.22	0.0003
AB57_RS14380	AB57_2904	type I-F CRISPR-associated protein Csy1	-8.22	0.0000
AB57_RS12490	AB57_2520	tRNA uridine 5-carboxymethylaminomethyl modification enzyme MnmG	-8.21	0.0000
AB57_RS15415		hypothetical protein	-8.20	0.0001
AB57_RS18920	AB57_3819	glutamate--cysteine ligase	-8.19	0.0006
AB57_RS02575	AB57_0493	membrane protein	-8.19	0.0003
AB57_RS05115	AB57_1006	MULTISPECIES: choline transporter BetT	-8.19	0.0018
AB57_RS19025	AB57_3840	N utilization substance protein B homolog	-8.18	0.0027

Table S2. continued.

Locus tag	Locus tag	Function	Log_2 Fitness defect in spleen	*P* value
AB57_RS18720	AB57_3778	peptidase M23	-8.18	0.0000
AB57_RS03155	AB57_0611	MULTISPECIES: glutaredoxin	-8.18	0.0029
AB57_RS01295	AB57_0255	MULTISPECIES: arsenate reductase	-8.17	0.0002
AB57_RS19065	AB57_3852	hypothetical protein	-8.17	0.0044
AB57_RS02060	AB57_0391	UvrABC system protein C	-8.17	0.0015
AB57_RS14820	AB57_2995	Holliday junction DNA helicase RuvB	-8.16	0.0035
AB57_RS01665	AB57_0317	DNA polymerase III subunit chi	-8.16	0.0039
AB57_RS19145	AB57_3868	maleylacetoacetate isomerase	-8.15	0.0000
AB57_RS17035	AB57_3446	MULTISPECIES: L-rhamnonate dehydratase	-8.15	0.0083
AB57_RS04905	AB57_0960	MULTISPECIES: hypothetical protein	-8.15	0.0000
AB57_RS03530	AB57_0691	glmZ(sRNA)-inactivating NTPase	-8.13	0.0037
AB57_RS02145	AB57_0408	N-methyltransferase	-8.13	0.0008
AB57_RS04205	AB57_0821	MULTISPECIES: hypothetical protein	-8.13	0.0030
AB57_RS16120	AB57_3266	oxidoreductase	-8.13	0.0015
AB57_RS04170	AB57_0814	cold-shock protein	-8.12	0.0002
AB57_RS01370		MULTISPECIES: mercury resistance protein	-8.11	0.0037
AB57_RS07340	AB57_1460	hypothetical protein	-8.10	0.0002
AB57_RS06905	AB57_1367	cold-shock protein	-8.10	0.0002
AB57_RS14015	AB57_2822	acinetobactin utilization protein	-8.10	0.0008
AB57_RS16800	AB57_3404	hypothetical protein	-8.10	0.0040
AB57_RS10150	AB57_2035	hypothetical protein	-8.09	0.0052
AB57_RS06010	AB57_1189	MULTISPECIES: hypothetical protein, partial	-8.09	0.0003
AB57_RS08950	AB57_1795	cobyric acid synthase CobQ	-8.09	0.0001
AB57_RS14245	AB57_2870	surface adhesion protein	-8.09	0.0055
AB57_RS14230	AB57_2867	MULTISPECIES: High frequency lysogenization protein HflD homolog	-8.08	0.0034
AB57_RS00645	AB57_0128	hypothetical protein	-8.08	0.0000
AB57_RS00390	AB57_0075	MULTISPECIES: phosphoglycolate phosphatase	-8.08	0.0000
AB57_RS17850	AB57_3606	FAD-linked oxidase	-8.08	0.0031
AB57_RS15240	AB57_3085	phosphoenolpyruvate carboxykinase	-8.05	0.0004

Table S2. continued.

Locus tag	Locus tag	Function	Log_2 Fitness defect in spleen	*P* value
AB57_RS01875	AB57_0359	pseudogene	-8.05	0.0039
AB57_RS05150	AB57_1013	peptidylprolyl isomerase	-8.05	0.0012
AB57_RS12555	AB57_2533	TetR family transcriptional regulator	-8.05	0.0075
AB57_RS15525	AB57_3144	HxlR family transcriptional regulator	-8.04	0.0001
AB57_RS08995	AB57_1805	deoxyribonuclease	-8.04	0.0000
AB57_RS14185	AB57_2858	LysR family transcriptional regulator	-8.04	0.0016
AB57_RS18730	AB57_RS18730	pseudogene	-8.03	0.0031
AB57_RS09025	AB57_1811	6-carboxy-5,6,7,8-tetrahydropterin synthase	-8.03	0.0075
AB57_RS04765	AB57_0932	outer membrane protein	-8.03	0.0001
AB57_RS13090	AB57_2639	pseudogene	-8.02	0.0048
AB57_RS01645	AB57_0313	MULTISPECIES: restriction endonuclease subunit M	-8.02	0.0040
AB57_RS00585	AB57_0116	L-lactate permease	-8.01	0.0018
AB57_RS19470		alcohol dehydrogenase	-8.01	0.0049
AB57_RS03470	AB57_RS03470	pseudogene	-8.00	0.0075
AB57_RS05550	AB57_1099	methyltransferase	-8.00	0.0000
AB57_RS03450	AB57_0674	MULTISPECIES: ribosome silencing factor RsfS	-8.00	0.0013
AB57_RS07335	AB57_1459	hypothetical protein	-8.00	0.0094
AB57_RS02355	AB57_0448	type II secretion system protein GspG	-7.99	0.0025
AB57_RS19380	AB57_3917	membrane protein	-7.99	0.0094
AB57_RS03610	AB57_0707	hypothetical protein	-7.99	0.0002
AB57_RS16805	AB57_3403	glycosyl transferase	-7.99	0.0017
GenBank:repeat_region:NC_011586:383486:384688			-7.98	0.0048
AB57_RS14650	AB57_2960	GTPase Era	-7.98	0.0060
AB57_RS12195	AB57_2458	membrane protein	-7.97	0.0030
AB57_RS19280	AB57_3896	2,5-diketo-D-gluconic acid reductase	-7.97	0.0053
AB57_RS08620	AB57_1728	amino acid ABC transporter ATP-binding protein	-7.97	0.0030
AB57_RS01910	AB57_0362	elongation factor Tu	-7.97	0.0048
AB57_RS11155	AB57_2240	hypothetical protein	-7.97	0.0008

Table S2. continued.

Locus tag	Locus tag	Function	Log_2 Fitness defect in spleen	*P* value
AB57_RS03250	AB57_0630	hypothetical protein	-7.97	0.0000
AB57_RS01570		MULTISPECIES: hypothetical protein	-7.97	0.0018
AB57_RS03845	AB57_0753	MULTISPECIES: D-ribulose-5-phosphate 3-epimerase	-7.97	0.0008
AB57_RS01195	AB57_0234	membrane protein	-7.95	0.0017
AB57_RS18465	AB57_3725	MULTISPECIES: hypothetical protein	-7.94	0.0001
AB57_RS19385	AB57_3918	glutathione S-transferase	-7.94	0.0008
AB57_RS17595	AB57_3552	mammalian cell entry protein	-7.94	0.0009
AB57_RS01920	AB57_0363	preprotein translocase subunit SecE	-7.93	0.0030
AB57_RS00315	AB57_0060	MULTISPECIES: lipoprotein signal peptidase	-7.93	0.0038
AB57_RS16200	AB57_3283	MULTISPECIES: rod shape-determining protein MreB	-7.93	0.0010
AB57_RS02905	AB57_0559	MULTISPECIES: hypothetical protein	-7.93	0.0008
AB57_RS03070	AB57_0594	hypothetical protein	-7.93	0.0044
AB57_RS05490	AB57_1086	MULTISPECIES: LysR family transcriptional regulator	-7.91	0.0027
AB57_RS16115	AB57_3265	molecular chaperone	-7.91	0.0044
AB57_RS03645	AB57_0714	MULTISPECIES: membrane protein	-7.91	0.0017
AB57_RS17740	AB57_3582	hypothetical protein	-7.91	0.0027
AB57_RS00335	AB57_0064	5\'-nucleosidase	-7.90	0.0038
AB57_RS18305	AB57_3692	homoserine kinase	-7.90	0.0052
AB57_RS01100	AB57_0213	cysteine synthase A	-7.90	0.0000
AB57_RS00755	AB57_0149	enoly-CoA hydratase	-7.89	0.0035
AB57_RS01165	AB57_0228	membrane protein	-7.89	0.0000
AB57_RS17605	AB57_3554	ABC transporter ATP-binding protein	-7.89	0.0004
AB57_RS02900		MULTISPECIES: hypothetical protein	-7.89	0.0006
AB57_RS14970	AB57_3029	RND transporter	-7.88	0.0094
AB57_RS09630	AB57_1934	MULTISPECIES: hypothetical protein	-7.88	0.0000
AB57_RS06170	AB57_1221	hypothetical protein	-7.87	0.0001
AB57_RS01660	AB57_0316	hypothetical protein	-7.87	0.0046
AB57_RS02795	AB57_0538	peptide-methionine (S)-S-oxide reductase	-7.87	0.0001
AB57_RS18180	AB57_3668	RND transporter	-7.87	0.0034
AB57_RS01620	AB57_0308	hypothetical protein	-7.86	0.0002

Table S2. continued.

Locus tag	Locus tag	Function	Log_2 Fitness defect in spleen	*P* value
AB57_RS18030	AB57_3642	3-dehydroquinate synthase	-7.86	0.0032
AB57_RS18945	AB57_3824	MATE family efflux transporter	-7.86	0.0025
AB57_RS13875	AB57_2792	xanthine dehydrogenase	-7.86	0.0003
AB57_RS16575	AB57_3356	3-ketoacyl-CoA thiolase	-7.86	0.0000
AB57_RS15140	AB57_3065	hypothetical protein	-7.86	0.0016
AB57_RS16520	AB57_3344	membrane protein	-7.84	0.0024
AB57_RS00810	AB57_0160	hypothetical protein	-7.84	0.0041
AB57_RS04860	AB57_0951	hypothetical protein	-7.84	0.0003
AB57_RS08600	AB57_1724	glutathione S-transferase	-7.82	0.0031
AB57_RS14805	AB57_2992	phosphoribosylformylglycinamidine synthase	-7.82	0.0046
AB57_RS14670	AB57_2964	elongation factor 4	-7.82	0.0002
AB57_RS02360	AB57_0449	hypothetical protein	-7.82	0.0002
AB57_RS05460	AB57_1080	MULTISPECIES: sulfate adenylyltransferase subunit 2	-7.81	0.0026
AB57_RS15345	AB57_3103	transcription elongation factor GreA	-7.81	0.0034
AB57_RS00330	AB57_0063	C4-dicarboxylate ABC transporter	-7.81	0.0003
AB57_RS18710	AB57_3776	dihydrolipoamide acetyltransferase	-7.80	0.0015
AB57_RS14385	AB57_2905	type I-F CRISPR-associated helicase Cas3	-7.80	0.0094
AB57_RS03650	AB57_0715	DNA polymerase I	-7.79	0.0036
AB57_RS04080	AB57_0796	MULTISPECIES: DNA-binding response regulator	-7.79	0.0080
AB57_RS14180	AB57_2857	ATP-dependent protease	-7.79	0.0040
AB57_RS02340	AB57_0445	MULTISPECIES: potassium transporter	-7.78	0.0000
AB57_RS16785	AB57_3400	glycosyl transferase	-7.78	0.0027
GenBank:repeat_region:NC_011586:308009:309004			-7.77	0.0036
AB57_RS18940	AB57_3823	aminotransferase	-7.77	0.0034
AB57_RS08520	AB57_1706	phosphoribosylglycinamide formyltransferase	-7.77	0.0005
AB57_RS12945	AB57_2607	amidophosphoribosyltransferase	-7.76	0.0013
AB57_RS14215	AB57_2864	hypothetical protein	-7.76	0.0000
AB57_RS17825	AB57_3600	multidrug transporter	-7.75	0.0018

Table S2. continued.

Locus tag	Locus tag	Function	Log_2 Fitness defect in spleen	*P* value
AB57_RS12920	AB57_2602	GatB/Yqey domain protein	-7.75	0.0001
AB57_RS13780	AB57_2776	Nif3-like dinuclear metal center hexameric protein	-7.75	0.0075
AB57_RS03705	AB57_0727	hypothetical protein	-7.75	0.0008
AB57_RS12515	AB57_2525	purine biosynthesis protein purH	-7.75	0.0031
AB57_RS03085	AB57_0597	glycosyl transferase	-7.73	0.0002
AB57_RS06015	AB57_1190	hypothetical protein	-7.73	0.0002
AB57_RS03160	AB57_0612	MULTISPECIES: rhodanese	-7.73	0.0010
AB57_RS15300	AB57_3094	hypothetical protein	-7.73	0.0030
AB57_RS03285	AB57_0639	LysR family transcriptional regulator	-7.73	0.0038
AB57_RS00690	AB57_0136	hypothetical protein	-7.71	0.0000
AB57_RS17510	AB57_3534	ATP-binding protein	-7.71	0.0001
AB57_RS03510	AB57_0687	poly(A) polymerase	-7.71	0.0091
AB57_RS17910	AB57_3618	MULTISPECIES: 30S ribosomal protein S16	-7.71	0.0049
AB57_RS06030	AB57_1193	feruloyl-CoA synthase	-7.70	0.0011
AB57_RS12145	AB57_2448	DNA mismatch repair protein MutL	-7.70	0.0009
AB57_RS19080	AB57_3855	hypothetical protein	-7.69	0.0044
AB57_RS13715	AB57_2763	lipoyl synthase	-7.69	0.0015
AB57_RS07430	AB57_1480	type VI secretion protein EvpB	-7.69	0.0008
AB57_RS14645		hypothetical protein	-7.68	0.0046
AB57_RS01220	AB57_0240	dihydrodipicolinate synthase	-7.68	0.0000
AB57_RS07030	AB57_1393	hypothetical protein	-7.67	0.0030
AB57_RS00615	AB57_0122	MULTISPECIES: 2-methylisocitrate lyase	-7.67	0.0038
AB57_RS04250	AB57_0830	hypothetical protein	-7.67	0.0002
AB57_RS18680	AB57_3770	inosine-5-monophosphate dehydrogenase	-7.67	0.0065
AB57_RS16350	AB57_3308	lysophospholipase	-7.66	0.0015
AB57_RS02125	AB57_0404	pseudogene	-7.66	0.0019
AB57_RS11785	AB57_2372	phosphotyrosine protein phosphatase	-7.65	0.0030
AB57_RS11775	AB57_2370	hypothetical protein	-7.65	0.0002
AB57_RS03170	AB57_0614	MULTISPECIES: oligoribonuclease	-7.65	0.0055
AB57_RS00925	AB57_0181	DNA repair protein HhH-GPD	-7.64	0.0026

Table S2. continued.

Locus tag	Locus tag	Function	Log_2 Fitness defect in spleen	*P* value
AB57_RS02110	AB57_0401	MULTISPECIES: hypothetical protein	-7.64	0.0022
AB57_RS14910	AB57_3014	MULTISPECIES: phosphoribosylformylglycinamidine cyclo-ligase	-7.63	0.0011
AB57_RS00205	AB57_0042	MULTISPECIES: phosphoribosylaminoimidazole carboxylase	-7.63	0.0026
AB57_RS16955	AB57_3434	oxidoreductase	-7.63	0.0012
AB57_RS19195	AB57_3878	MULTISPECIES: phosphoribosylaminoimidazole-succinocarboxamide synthase	-7.62	0.0031
AB57_RS02690	AB57_0517	hypothetical protein	-7.62	0.0001
AB57_RS01270	AB57_0250	MULTISPECIES: thioredoxin reductase	-7.61	0.0011
AB57_RS18625	AB57_3758	hypothetical protein	-7.61	0.0008
AB57_RS16590	AB57_3359	patatin family protein	-7.61	0.0075
AB57_RS03150	AB57_0610	protein-export protein SecB	-7.61	0.0042
AB57_RS00720	AB57_0142	alanine glycine permease	-7.60	0.0035
AB57_RS01700	AB57_0324	histidine kinase	-7.59	0.0032
AB57_RS01385	AB57_0272	MULTISPECIES: mercury transporter MerC	-7.59	0.0028
AB57_RS14710	AB57_2972	ABC transporter permease	-7.59	0.0003
AB57_RS05185	AB57_1020	MULTISPECIES: IclR family transcriptional regulator	-7.59	0.0003
AB57_RS00775	AB57_0153	LuxR family transcriptional regulator	-7.58	0.0020
AB57_RS01535	AB57_0293	MULTISPECIES: ANT(3\'\')-Ia family aminoglycoside nucleotidyltransferase AadA1	-7.58	0.0026
AB57_RS05260	AB57_1035	MFS transporter permease	-7.57	0.0003
AB57_RS07540	AB57_1502	hypothetical protein	-7.56	0.0022
AB57_RS00680	AB57_0134	polyketide cyclase	-7.56	0.0011
AB57_RS02470	AB57_0472	MATE family efflux transporter	-7.54	0.0021
AB57_RS00425	AB57_0082	MULTISPECIES: ribonuclease PH	-7.53	0.0001
AB57_RS00785	AB57_0155	acyl-CoA dehydrogenase	-7.53	0.0000
AB57_RS03825	AB57_0749	methyltransferase	-7.52	0.0001
AB57_RS03695	AB57_0725	MULTISPECIES: anti-anti-sigma factor	-7.52	0.0009
AB57_RS17010	AB57_3441	phosphohydrolase	-7.51	0.0003
AB57_RS14205	AB57_2862	hypothetical protein	-7.51	0.0001

Table S2. continued.

Locus tag	Locus tag	Function	Log_2 Fitness defect in spleen	*P* value
AB57_RS03505	AB57_0686	transporter	-7.51	0.0001
AB57_RS00080	AB57_0016	cytochrome B	-7.50	0.0031
AB57_RS18175	AB57_3667	hemolysin D	-7.50	0.0006
AB57_RS03425	AB57_0669	transporter	-7.49	0.0001
AB57_RS18165	AB57_3665	cation transporter	-7.49	0.0003
AB57_RS14680	AB57_2966	serine protease	-7.49	0.0000
AB57_RS14480	AB57_RS14480	pseudogene	-7.49	0.0075
AB57_RS02130	AB57_0405	23S rRNA (guanosine-2\'-O-)-methyltransferase	-7.49	0.0043
AB57_RS02335	AB57_0444	heat-shock protein Hsp33	-7.49	0.0000
AB57_RS03255	AB57_0631	peptide chain release factor 3	-7.48	0.0044
AB57_RS08925	AB57_1790	peptidylprolyl isomerase	-7.48	0.0045
AB57_RS16545	AB57_3349	hypothetical protein	-7.48	0.0026
AB57_RS18985	AB57_3832	nicotinate phosphoribosyltransferase	-7.48	0.0005
AB57_RS03775	AB57_0739	protein FilA	-7.48	0.0041
AB57_RS01425		MULTISPECIES: hypothetical protein	-7.47	0.0030
AB57_RS02930	AB57_0565	MULTISPECIES: endonuclease	-7.46	0.0011
AB57_RS17305	AB57_3493	ribonuclease R	-7.46	0.0049
AB57_RS05165	AB57_1016	poly-beta-1,6-N-acetyl-D-glucosamine biosynthesis protein PgaD	-7.45	0.0001
AB57_RS19420	AB57_3921	MULTISPECIES: DNA replication protein	-7.45	0.0016
AB57_RS17665	AB57_3566	membrane protein	-7.45	0.0001
AB57_RS05605	AB57_1111	lon protease	-7.45	0.0013
AB57_RS16655	AB57_3373	tryptophan synthase subunit alpha	-7.45	0.0052
AB57_RS15725	AB57_4816	tRNA-Arg	-7.45	0.0014
AB57_RS04730	AB57_0925	threonine transporter RhtB	-7.45	0.0003
AB57_RS14800	AB57_2991	Bcr/CflA family drug resistance efflux transporter	-7.45	0.0000
AB57_RS18025	AB57_3641	hypothetical protein	-7.45	0.0018
AB57_RS05410	AB57_1069	polyphosphate kinase	-7.44	0.0008
AB57_RS01650	AB57_0314	riboflavin synthase subunit alpha	-7.44	0.0060
AB57_RS17600	AB57_3553	ABC transporter permease	-7.43	0.0023

Table S2. continued.

Locus tag	Locus tag	Function	Log_2 Fitness defect in spleen	*P* value
AB57_RS16480	AB57_3334	hypothetical protein	-7.43	0.0000
AB57_RS04545	AB57_0888	competence protein ComL	-7.42	0.0003
AB57_RS02865	AB57_RS02865	pseudogene	-7.42	0.0015
AB57_RS16475	AB57_3333	peptidyl-prolyl cis-trans isomerase	-7.42	0.0003
AB57_RS01185	AB57_0232	DNA topoisomerase IV subunit A	-7.42	0.0075
AB57_RS17180	AB57_3472	hypothetical protein	-7.41	0.0044
AB57_RS00815	AB57_0161	phosphopantetheine-protein transferase	-7.41	0.0025
AB57_RS02620	AB57_0502	tRNA pseudouridine synthase A	-7.41	0.0040
AB57_RS15445	AB57_3128	succinate dehydrogenase iron-sulfur subunit	-7.41	0.0032
AB57_RS05455	AB57_1078	hypothetical protein	-7.40	0.0021
AB57_RS16920	AB57_3428	tRNA 5-methylaminomethyl-2-thiouridine biosynthesis bifunctional protein MnmC	-7.40	0.0026
AB57_RS06775	AB57_1343	MULTISPECIES: 3-oxoacyl-ACP synthase	-7.40	0.0069
AB57_RS13745	AB57_2769	RNA methyltransferase	-7.40	0.0001
AB57_RS11325	AB57_2275	MULTISPECIES: glutaredoxin	-7.40	0.0026
AB57_RS05425	AB57_1072	alpha/beta hydrolase	-7.39	0.0038
AB57_RS16275	AB57_3299	membrane protein	-7.38	0.0031
AB57_RS13405	AB57_2702	head morphogenesis protein	-7.38	0.0030
AB57_RS07145	AB57_1417	hypothetical protein	-7.38	0.0010
AB57_RS03685	AB57_0723	hypothetical protein	-7.38	0.0026
AB57_RS11375	AB57_2286	1-phosphofructokinase	-7.37	0.0009
AB57_RS08755	AB57_1756	N-acetyltransferase	-7.36	0.0017
AB57_RS16665	AB57_3375	pseudogene	-7.36	0.0012
AB57_RS01445		MULTISPECIES: hypothetical protein	-7.36	0.0002
AB57_RS11335	AB57_2277	LysR family transcriptional regulator	-7.35	0.0001
AB57_RS17575	AB57_3547	hypothetical protein	-7.35	0.0001
AB57_RS13630	AB57_2747	hypothetical protein	-7.35	0.0040
AB57_RS17835	AB57_3603	NADPH quinone reductase MdaB	-7.34	0.0003
AB57_RS18930	AB57_3821	hypothetical protein	-7.34	0.0045
AB57_RS02135	AB57_0407	hypothetical protein	-7.34	0.0000

Table S2. continued.

Locus tag	Locus tag	Function	Log_2 Fitness defect in spleen	*P* value
AB57_RS13645	AB57_2750	hypothetical protein	-7.33	0.0003
AB57_RS00535	AB57_0106	acetyltransferase	-7.33	0.0049
AB57_RS17080	AB57_3455	hypothetical protein	-7.33	0.0006
AB57_RS13610	AB57_2742	integrase	-7.33	0.0030
AB57_RS04590	AB57_0896	transcriptional regulator	-7.33	0.0001
AB57_RS06350	AB57_1257	pseudogene	-7.33	0.0003
AB57_RS03800	AB57_0744	protein FilF	-7.33	0.0079
AB57_RS05565	AB57_1102	membrane protein	-7.32	0.0021
AB57_RS18875	AB57_3810	esterase	-7.31	0.0031
AB57_RS00195	AB57_0040	UDP-N-acetylmuramate:L-alanyl-gamma-D-glutamyl-meso-diaminopimelate ligase	-7.31	0.0006
AB57_RS02050	AB57_0389	MULTISPECIES: aspartyl/asparaginyl beta-hydroxylase	-7.31	0.0000
AB57_RS17965	AB57_3629	hypothetical protein	-7.30	0.0083
AB57_RS15540	AB57_3147	serine--tRNA ligase	-7.30	0.0003
AB57_RS02105	AB57_0400	MULTISPECIES: Holliday junction DNA helicase	-7.30	0.0016
AB57_RS17050	AB57_3449	dimethyladenosine transferase	-7.30	0.0050
AB57_RS04335	AB57_0848	MULTISPECIES: GntR family transcriptional regulator	-7.30	0.0003
AB57_RS18205	AB57_3673	O-acetylhomoserine aminocarboxypropyltransferase	-7.29	0.0012
AB57_RS15615	AB57_3162	MULTISPECIES: oxidoreductase	-7.29	0.0026
AB57_RS10540	AB57_2113	NUDIX hydrolase	-7.29	0.0008
AB57_RS03210	AB57_0622	leucine--tRNA ligase	-7.29	0.0039
AB57_RS00520	AB57_0103	glycosyl transferase family 1	-7.29	0.0094
AB57_RS00155	AB57_0032	hypothetical protein	-7.28	0.0047
AB57_RS03835	AB57_0751	esterase	-7.28	0.0026
AB57_RS16910	AB57_RS16910	pseudogene	-7.28	0.0001
AB57_RS14260	AB57_2873	high frequency lysogenization protein HflD	-7.28	0.0062
AB57_RS18475	AB57_3727	NAD-dependent succinate-semialdehyde dehydrogenase	-7.28	0.0035
AB57_RS18630	AB57_3760	transporter	-7.28	0.0022
AB57_RS16890	AB57_3420	hypothetical protein	-7.28	0.0002
AB57_RS05095	AB57_1002	oxygen-dependent choline dehydrogenase	-7.27	0.0057

Table S2. continued.

Locus tag	Locus tag	Function	Log_2 Fitness defect in spleen	*P* value
AB57_RS12180	AB57_2455	membrane protein	-7.27	0.0011
AB57_RS00005	AB57_0001	tyrosine--tRNA ligase	-7.26	0.0094
AB57_RS00100	AB57_0020	chromosomal replication initiator protein DnaA	-7.26	0.0023
AB57_RS03245	AB57_0629	TetR family transcriptional regulator	-7.25	0.0072
AB57_RS00250	AB57_0051	hypothetical protein	-7.25	0.0032
AB57_RS01190	AB57_0233	long-chain fatty acid--CoA ligase	-7.25	0.0004
AB57_RS02995	AB57_0579	trigger factor	-7.24	0.0001
AB57_RS15750	AB57_3190	hypothetical protein	-7.23	0.0094
AB57_RS05470	AB57_1082	hypothetical protein	-7.22	0.0049
AB57_RS03765	AB57_0737	histidinol-phosphate aminotransferase	-7.22	0.0004
AB57_RS00075	AB57_0015	membrane protein	-7.22	0.0022
AB57_RS16170	AB57_3277	O-acetyltransferase	-7.22	0.0007
AB57_RS03025	AB57_0585	phosphate acetyltransferase	-7.22	0.0001
AB57_RS04830	AB57_0945	type IV pili twitching motility protein PilT	-7.22	0.0005
AB57_RS18620	AB57_3756	acetyl-coenzyme A synthetase	-7.22	0.0000
AB57_RS12500	AB57_2522	ribosomal protein L11 methyltransferase	-7.21	0.0008
AB57_RS00410	AB57_0079	TetR family transcriptional regulator	-7.21	0.0019
AB57_RS17915	AB57_3619	pilus assembly protein PilE	-7.21	0.0002
AB57_RS02785	AB57_0536	MULTISPECIES: biopolymer transporter ExbB	-7.20	0.0030
AB57_RS02915	AB57_0562	MULTISPECIES: hypothetical protein	-7.20	0.0002
AB57_RS09220	AB57_1850	MULTISPECIES: guanylate cyclase	-7.20	0.0001
AB57_RS01715	AB57_0327	threonine synthase	-7.19	0.0039
AB57_RS05730	AB57_1138	microcin C ABC transporter permease YejB	-7.19	0.0008
AB57_RS12460	AB57_2513	alanine racemase	-7.18	0.0002
AB57_RS16670	AB57_3377	hypothetical protein	-7.18	0.0015
AB57_RS18575	AB57_3747	actetate permease	-7.18	0.0052
AB57_RS05795	AB57_1149	MULTISPECIES: ribonuclease T	-7.18	0.0002
AB57_RS05330	AB57_1052	D-serine dehydratase	-7.17	0.0038
AB57_RS17645	AB57_3562	hypothetical protein	-7.17	0.0050

Table S2. continued.

Locus tag	Locus tag	Function	Log_2 Fitness defect in spleen	*P* value
AB57_RS14925	AB57_3017	peptidase S49	-7.17	0.0011
AB57_RS07695	AB57_1533	helicase	-7.17	0.0075
AB57_RS13775	AB57_2775	2-alkenal reductase	-7.17	0.0005
AB57_RS01065	AB57_RS01065	pseudogene	-7.17	0.0035
AB57_RS15735	AB57_3186	hypothetical protein	-7.17	0.0055
AB57_RS17560	AB57_3544	ATP-dependent DNA helicase RecG	-7.17	0.0047
AB57_RS09370	AB57_1880	siderophore biosynthesis protein	-7.16	0.0030
AB57_RS02645	AB57_0508	endonuclease	-7.16	0.0000
AB57_RS08930	AB57_1791	LPS-assembly protein LptD	-7.16	0.0094
AB57_RS04165	AB57_0813	uracil phosphoribosyltransferase	-7.16	0.0048
AB57_RS03165	AB57_0613	GTPase	-7.16	0.0031
AB57_RS01150	AB57_RS01150	pseudogene	-7.15	0.0000
AB57_RS07180	AB57_1424	DNA mismatch repair protein MutS	-7.14	0.0016
AB57_RS07140	AB57_1416	secretion protein HlyD	-7.14	0.0000
AB57_RS15325	AB57_3099	hypothetical protein	-7.13	0.0026
AB57_RS01825	AB57_0349	DNA-binding response regulator	-7.13	0.0023
AB57_RS04840	AB57_0947	ATP-dependent dsDNA exonuclease	-7.12	0.0009
AB57_RS07100		cysteinyl-tRNA synthetase	-7.12	0.0016
AB57_RS19200	AB57_3879	hypothetical protein	-7.12	0.0003
AB57_RS18790	AB57_3793	arsenate reductase	-7.12	0.0006
AB57_RS15425	AB57_3123	MULTISPECIES: succinyl-CoA ligase subunit beta	-7.12	0.0053
AB57_RS17655	AB57_3564	hypothetical protein	-7.12	0.0093
AB57_RS18960	AB57_3827	MULTISPECIES: DNA-binding response regulator	-7.11	0.0028
AB57_RS12495	AB57_2521	MULTISPECIES: hypothetical protein	-7.11	0.0002
AB57_RS03115	AB57_0603	DNA-binding protein	-7.11	0.0066
AB57_RS16555	AB57_3351	glucose dehydrogenase	-7.10	0.0004
AB57_RS13000		oxidoreductase	-7.10	0.0011
AB57_RS05085		hypothetical protein	-7.10	0.0008
AB57_RS03175	AB57_0615	membrane protein	-7.10	0.0020

Table S2. continued.

Locus tag	Locus tag	Function	Log_2 Fitness defect in spleen	*P* value
AB57_RS06735	AB57_1335	glutathionine S-transferase	-7.10	0.0016
AB57_RS01455		transposase	-7.10	0.0006
AB57_RS03080	AB57_0596	peptidase S41	-7.09	0.0000
AB57_RS04005	AB57_0781	MULTISPECIES: hypothetical protein	-7.09	0.0030
AB57_RS15235	AB57_3084	diacylglycerol kinase	-7.09	0.0001
AB57_RS15890	AB57_3220	methyltransferase	-7.08	0.0008
AB57_RS16915	AB57_3426	hypothetical protein	-7.08	0.0006
AB57_RS08965	AB57_1798	MULTISPECIES: biopolymer transporter ExbD	-7.08	0.0030
AB57_RS17045	AB57_3448	diadenosine tetraphosphatase	-7.08	0.0027
AB57_RS12875	AB57_4792		-7.08	0.0006
AB57_RS00310	AB57_0059	MULTISPECIES: peptidylprolyl isomerase	-7.07	0.0002
AB57_RS02970	AB57_0574	MULTISPECIES: methionine biosynthesis protein MetW	-7.07	0.0019
AB57_RS17320	AB57_3496	hypothetical protein	-7.06	0.0003
AB57_RS08875	AB57_1780	hypothetical protein	-7.06	0.0010
AB57_RS17550	AB57_3542	RND transporter	-7.06	0.0003
AB57_RS19035		phosphatidylglycerophosphatase A	-7.06	0.0002
AB57_RS14815	AB57_2994	Holliday junction DNA helicase RuvA	-7.05	0.0000
AB57_RS16700	AB57_3383	glycerophosphoryl diester phosphodiesterase	-7.05	0.0001
AB57_RS12870	AB57_4829	phosphohistidine phosphatase	-7.05	0.0006
AB57_RS15610	AB57_3160	polyketide cyclase	-7.05	0.0039
AB57_RS00685	AB57_0135	ligand-gated channel protein	-7.05	0.0004
GenBank:STS:NC_011586:1492875:1494163	D1S3688		-7.05	0.0038
AB57_RS12185	AB57_2456	transcriptional regulator	-7.04	0.0000
AB57_RS03040	AB57_0588	2-dehydro-3-deoxyphosphogluconate aldolase	-7.04	0.0055
AB57_RS04485	AB57_0880	peptidyl-tRNA hydrolase	-7.04	0.0059
AB57_RS01555		MULTISPECIES: hypothetical protein	-7.04	0.0030
AB57_RS16485	AB57_3335	MULTISPECIES: large-conductance mechanosensitive channel	-7.03	0.0052

Table S2. continued.

Locus tag	Locus tag	Function	Log$_2$ Fitness defect in spleen	*P* value
AB57_RS09215	AB57_1849	MULTISPECIES: hypothetical protein	-7.03	0.0002
AB57_RS02395	AB57_0456	hypothetical protein	-7.03	0.0001
AB57_RS18910	AB57_3817	type VI secretion system protein	-7.02	0.0002
AB57_RS00675	AB57_0133	hypothetical protein	-7.02	0.0002
AB57_RS11395	AB57_2290	hypothetical protein	-7.02	0.0002
AB57_RS03855	AB57_0755	hypothetical protein	-7.01	0.0000
AB57_RS17100	AB57_3459	acetyl-CoA carboxylase carboxyltransferase subunit	-7.00	0.0010
AB57_RS02585	AB57_0495	3-isopropylmalate dehydratase small subunit	-7.00	0.0012
GenBank:repeat_region:NC_011586:275891:279266			-7.00	0.0034
AB57_RS16130	AB57_3268	undecaprenyl-diphosphatase	-7.00	0.0055
AB57_RS18980	AB57_3831	thiosulfate sulfurtransferase	-6.99	0.0000
AB57_RS14210	AB57_2863	D-alanyl-D-alanine carboxypeptidase	-6.99	0.0035
AB57_RS01365		MULTISPECIES: hypothetical protein	-6.99	0.0002
AB57_RS17075	AB57_3454	peptidase	-6.98	0.0010
AB57_RS01490	AB57_0287	transposase	-6.98	0.0007
AB57_RS07015		phage head morphogenesis protein	-6.97	0.0017
AB57_RS14830	AB57_2997	MULTISPECIES: thioesterase	-6.96	0.0008
AB57_RS09990	AB57_2007	MULTISPECIES: ferrous iron transporter B	-6.96	0.0094
AB57_RS17880	AB57_3612	tRNA pseudouridine synthase B	-6.96	0.0047
AB57_RS13845	AB57_2786	anthranilate synthase subunit II	-6.96	0.0046
AB57_RS03005	AB57_0581	ATP-dependent Clp protease ATP-binding subunit ClpX	-6.96	0.0000
AB57_RS02705	AB57_0520	MULTISPECIES: hypothetical protein	-6.96	0.0016
AB57_RS04940	AB57_0967	hypothetical protein	-6.96	0.0087
AB57_RS00945	AB57_0185	glutathione peroxidase	-6.96	0.0062
AB57_RS19075	AB57_3854	haloacid dehalogenase	-6.96	0.0044
AB57_RS19250	AB57_3889	MULTISPECIES: hypothetical protein	-6.95	0.0000
AB57_RS19165	AB57_3872	hypothetical protein	-6.95	0.0018
AB57_RS19245	AB57_3888	peptidase M23	-6.95	0.0000

Table S2. continued.

Locus tag	Locus tag	Function	Log_2 Fitness defect in spleen	*P* value
AB57_RS18130	AB57_4798	tRNA-Glu	-6.95	0.0007
AB57_RS11125	AB57_2233	hypothetical protein	-6.95	0.0046
AB57_RS01850	AB57_0354	thioesterase	-6.94	0.0046
AB57_RS17660	AB57_3565	PspC family transcriptional regulator	-6.94	0.0017
AB57_RS07020	AB57_1391	hypothetical protein	-6.93	0.0094
AB57_RS02565	AB57_0491	haloacid dehalogenase	-6.92	0.0000
AB57_RS00550	AB57_RS00550	pseudogene	-6.92	0.0044
AB57_RS12560	AB57_2534	mutlidrug resistance protein	-6.91	0.0081
AB57_RS17625	AB57_3558	1-deoxy-D-xylulose-5-phosphate synthase	-6.91	0.0070
AB57_RS09625	AB57_1933	2,3-butanediol dehydrogenase	-6.90	0.0002
AB57_RS00940	AB57_0184	MULTISPECIES: DNA repair protein HhH-GPD	-6.90	0.0016
AB57_RS04875	AB57_0954	delta-aminolevulinic acid dehydratase	-6.90	0.0058
AB57_RS15605	AB57_3161	metal-binding protein	-6.90	0.0000
AB57_RS01210	AB57_0237	membrane protein	-6.90	0.0009
AB57_RS15185	AB57_3073	protease HtpX	-6.90	0.0013
AB57_RS04550	AB57_0889	23S rRNA pseudouridine synthase D	-6.89	0.0014
AB57_RS08955	AB57_1796	chromosome partitioning protein	-6.88	0.0026
AB57_RS00455	AB57_0089	peptidyl-prolyl cis-trans isomerase	-6.88	0.0027
AB57_RS14735	AB57_2978	CysB family transcriptional regulator	-6.87	0.0022
AB57_RS18435	AB57_3719	MFS transporter	-6.87	0.0050
AB57_RS16325	AB57_3303	peptidase	-6.87	0.0001
AB57_RS00820	AB57_4835	tRNA-Gly	-6.87	0.0054
AB57_RS00170	AB57_0035	carboxylate-amine ligase	-6.87	0.0025
AB57_RS19370	AB57_3914	hypothetical protein	-6.87	0.0011
AB57_RS17315	AB57_3495	metal-binding protein	-6.87	0.0014
GenBank:repeat_region:NC_011586:8613:12194			-6.87	0.0000
AB57_RS16635	AB57_3369	protein-tyrosine phosphatase	-6.86	0.0000
AB57_RS04720	AB57_0922	thioredoxin reductase	-6.86	0.0011

Table S2. continued.

Locus tag	Locus tag	Function	Log_2 Fitness defect in spleen	*P* value
AB57_RS09250	AB57_1856	cysteine desulfurase IscS	-6.85	0.0030
AB57_RS18445	AB57_3721	aldehyde-activating protein	-6.85	0.0022
AB57_RS04535	AB57_0887	glutamyl-tRNA reductase	-6.85	0.0055
AB57_RS04885	AB57_0956	MULTISPECIES: hemolysin D	-6.85	0.0026
AB57_RS14335	AB57_2891	membrane protein	-6.84	0.0048
AB57_RS04315	AB57_0844	signal recognition particle protein	-6.84	0.0030
AB57_RS03190	AB57_0618	MacB family efflux pump subunit	-6.84	0.0038
AB57_RS18885	AB57_3812	hypothetical protein	-6.84	0.0003
AB57_RS03380	AB57_0660	MULTISPECIES: two-component system sensor histidine kinase	-6.83	0.0094
AB57_RS12915	AB57_2601	30S ribosomal protein S21	-6.83	0.0003
AB57_RS18125	AB57_4826	tRNA-Ala	-6.83	0.0006
AB57_RS05480	AB57_1084	citrate transporter	-6.82	0.0000
AB57_RS14455	AB57_2921	isocitrate dehydrogenase	-6.82	0.0031
AB57_RS17955	AB57_3627	guanylate kinase	-6.82	0.0003
AB57_RS07480	AB57_1490	ATPase	-6.82	0.0052
AB57_RS15420	AB57_3122	MULTISPECIES: succinyl-CoA synthetase subunit alpha	-6.81	0.0001
AB57_RS07090		integrase	-6.81	0.0010
AB57_RS14465	AB57_2923	D-alanyl-D-alanine carboxypeptidase	-6.80	0.0000
AB57_RS18845	AB57_3804	uracil permease	-6.80	0.0025
AB57_RS01955	AB57_0370	DNA-directed RNA polymerase subunit beta\'	-6.80	0.0003
AB57_RS03195	AB57_0619	MacA family efflux pump subunit	-6.80	0.0007
AB57_RS18105	AB57_3657	ribosomal RNA small subunit methyltransferase H	-6.80	0.0033
AB57_RS16610	AB57_3364	MFS transporter	-6.80	0.0034
AB57_RS05355	AB57_1057	membrane protein	-6.79	0.0002
AB57_RS18260	AB57_3682	MULTISPECIES: imidazoleglycerol-phosphate dehydratase	-6.79	0.0028
AB57_RS18815	AB57_3798	hypothetical protein	-6.79	0.0041
AB57_RS13070	AB57_2634	C4-dicarboxylate ABC transporter	-6.79	0.0042
AB57_RS17615	AB57_3556	DEAD/DEAH box helicase	-6.78	0.0015
AB57_RS03790	AB57_0742	protein FilD	-6.78	0.0027

Table S2. continued.

Locus tag	Locus tag	Function	Log_2 Fitness defect in spleen	*P* value
AB57_RS17265	AB57_3489	hypothetical protein	-6.77	0.0005
AB57_RS15135		hypothetical protein	-6.77	0.0030
AB57_RS18020	AB57_3640	glutamate synthase subunit alpha	-6.77	0.0012
AB57_RS13290	AB57_2681	hypothetical protein	-6.77	0.0028
AB57_RS03975	AB57_0775	long-chain fatty acid transporter	-6.77	0.0062
AB57_RS00670	AB57_0132	pseudogene	-6.77	0.0026
AB57_RS10845	AB57_2176	MULTISPECIES: GntR family transcriptional regulator	-6.77	0.0018
AB57_RS01865	AB57_0357	general secretion pathway protein	-6.76	0.0002
AB57_RS18300	AB57_3691	cold-shock protein	-6.76	0.0026
AB57_RS17165	AB57_3469	CDP-diacylglycerol--serine O-phosphatidyltransferase	-6.76	0.0001
AB57_RS13540	AB57_2730	hypothetical protein	-6.76	0.0014
AB57_RS18590	AB57_3750	hypothetical protein	-6.76	0.0003
AB57_RS00225	AB57_0046	hypothetical protein	-6.76	0.0055
AB57_RS00800	AB57_0158	RND transporter	-6.75	0.0045
AB57_RS04185	AB57_0817	ferredoxin-NADP reductase	-6.75	0.0001
AB57_RS16640	AB57_3370	cell division protein	-6.75	0.0014
AB57_RS19330	AB57_3906	phosphoenolpyruvate carboxylase	-6.75	0.0010
AB57_RS03020	AB57_0584	fumarate hydratase	-6.74	0.0020
AB57_RS12505	AB57_2523	hypothetical protein	-6.74	0.0001
AB57_RS19205	AB57_3880	formate transporter	-6.74	0.0000
AB57_RS05335	AB57_1053	hypothetical protein	-6.74	0.0020
AB57_RS00045	AB57_0009	class C beta-lactamase	-6.74	0.0058
AB57_RS07095	AB57_4838	cysteine--tRNA ligase	-6.73	0.0010
AB57_RS05485	AB57_1085	hypothetical protein	-6.73	0.0030
AB57_RS01805	AB57_0345	MULTISPECIES: transcriptional regulator	-6.73	0.0003
AB57_RS15985	AB57_3238	nuclease	-6.73	0.0003
AB57_RS11575	AB57_2328	hypothetical protein	-6.73	0.0002
AB57_RS05580	AB57_1106	iron-sulfur protein	-6.72	0.0054
AB57_RS15670	AB57_3174	lipid A phosphoethanolamine transferase	-6.72	0.0022

Table S2. continued.

Locus tag	Locus tag	Function	Log_2 Fitness defect in spleen	*P* value
AB57_RS04465	AB57_0876	MULTISPECIES: 3,4-dihydroxy-2-butanone-4-phosphate synthase	-6.72	0.0062
AB57_RS18695	AB57_3773	ligand-gated channel protein	-6.71	0.0008
AB57_RS02630	AB57_0504	hypothetical protein	-6.71	0.0054
AB57_RS00025	AB57_0005	hypothetical protein	-6.71	0.0038
AB57_RS07675	AB57_1529	MULTISPECIES: PaaX family transcriptional regulator	-6.71	0.0003
AB57_RS12790	AB57_2580	sodium:glutamate symporter	-6.70	0.0020
AB57_RS11470	AB57_2305	haloacid dehalogenase	-6.70	0.0030
AB57_RS17960	AB57_3628	MULTISPECIES: DNA-directed RNA polymerase subunit omega	-6.69	0.0023
AB57_RS02485	AB57_0475	short-chain dehydrogenase	-6.69	0.0056
AB57_RS18665	AB57_3767	recombination protein RecJ	-6.69	0.0002
AB57_RS04810	AB57_0941	hypothetical protein	-6.68	0.0094
AB57_RS03760	AB57_0734	pseudogene	-6.68	0.0002
AB57_RS01200	AB57_0235	membrane protein	-6.68	0.0006
AB57_RS04215	AB57_0823	1-aminocyclopropane-1-carboxylate deaminase	-6.68	0.0058
AB57_RS18390	AB57_3710	HxlR family transcriptional regulator	-6.68	0.0015
GenBank:repeat_region:NC_011586:6676:8609			-6.67	0.0058
AB57_RS00265	AB57_0054	esterase	-6.67	0.0000
AB57_RS11230	AB57_2256	peptidase M16	-6.67	0.0026
AB57_RS18155	AB57_3663	hypothetical protein	-6.67	0.0017
AB57_RS01050	AB57_0206	TonB-dependent receptor	-6.67	0.0006
AB57_RS18925	AB57_3820	membrane protein	-6.67	0.0013
AB57_RS00355	AB57_0068	MULTISPECIES: alkanesulfonate monooxygenase	-6.67	0.0010
AB57_RS18525	AB57_3737	thiaminase II	-6.66	0.0008
AB57_RS02590	AB57_0496	hypothetical protein	-6.66	0.0062
AB57_RS16025	AB57_3246	DNA helicase	-6.66	0.0030
AB57_RS12355	AB57_2491	hypothetical protein	-6.66	0.0001
AB57_RS16705	AB57_3384	hypothetical protein	-6.66	0.0023

Table S2. continued.

Locus tag	Locus tag	Function	Log_2 Fitness defect in spleen	*P* value
AB57_RS17815	AB57_3598	hypothetical protein	-6.66	0.0003
AB57_RS12140	AB57_2447	tRNA dimethylallyltransferase	-6.65	0.0002
AB57_RS16465		formamidopyrimidine-DNA glycosylase	-6.65	0.0008
AB57_RS08725	AB57_1749	hypothetical protein	-6.65	0.0002
AB57_RS01345	AB57_RS01345	pseudogene	-6.65	0.0000
AB57_RS03830	AB57_0750	hypothetical protein	-6.65	0.0024
AB57_RS17610		hypothetical protein	-6.65	0.0003
AB57_RS04845	AB57_0948	ATP-dependent dsDNA exonuclease	-6.65	0.0052
AB57_RS15595	AB57_3158	adeC/adeK/oprM family multidrug efflux complex outer membrane factor	-6.65	0.0017
AB57_RS16185	AB57_3280	septum formation inhibitor Maf	-6.64	0.0012
AB57_RS00735	AB57_0145	3-hydroxyisobutyrate dehydrogenase	-6.64	0.0006
AB57_RS05370	AB57_1060	hypothetical protein	-6.63	0.0003
GenBank:STS:NC_011586:297629:297780	D13S22		-6.63	0.0000
AB57_RS00440	AB57_0085	nicotinate-nucleotide pyrophosphorylase	-6.63	0.0008
AB57_RS12880	AB57_4830	nitroreductase	-6.62	0.0016
AB57_RS17775	AB57_3590	S-methylmethionine transporter	-6.62	0.0011
AB57_RS17505	AB57_3533	cystathionine beta-lyase	-6.61	0.0000
AB57_RS13740	AB57_2768	5,10-methylenetetrahydrofolate reductase	-6.61	0.0026
AB57_RS00065	AB57_0013	RND transporter	-6.60	0.0054
AB57_RS02570	AB57_0492	MULTISPECIES: LysR family transcriptional regulator	-6.60	0.0008
AB57_RS15250	AB57_3087	glycerol kinase	-6.60	0.0023
AB57_RS04865	AB57_0953	nucleoside-diphosphate sugar epimerase	-6.60	0.0028
AB57_RS11210	AB57_2252	threonine dehydratase	-6.60	0.0009
AB57_RS06690	AB57_1325	ribosomal RNA large subunit methyltransferase K/L	-6.59	0.0000
AB57_RS18895	AB57_RS18895	pseudogene	-6.59	0.0009
AB57_RS16355	AB57_3309	coproporphyrinogen III oxidase	-6.58	0.0016

Table S2. continued.

Locus tag	Locus tag	Function	Log_2 Fitness defect in spleen	*P* value
AB57_RS05790	AB57_4797	tRNA-Glu	-6.57	0.0075
AB57_RS01265	AB57_0249	pyridine nucleotide-disulfide oxidoreductase	-6.57	0.0015
AB57_RS18380	AB57_3708	hypothetical protein	-6.57	0.0011
AB57_RS01110	AB57_0215	FMN reductase	-6.57	0.0030
AB57_RS15490	AB57_3137	RNA polymerase sigma factor RpoD	-6.57	0.0001
AB57_RS01710	AB57_0326	D-alanyl-D-alanine endopeptidase	-6.57	0.0011
AB57_RS15295	AB57_3093	hypothetical protein	-6.57	0.0022
AB57_RS02610	AB57_0500	MULTISPECIES: translation initiation factor IF-1	-6.57	0.0094
AB57_RS04880	AB57_0955	hypothetical protein	-6.56	0.0047
AB57_RS12360		hypothetical protein	-6.55	0.0004
AB57_RS02695	AB57_0518	Zn-dependent oxidoreductase	-6.55	0.0036
AB57_RS19325	AB57_3905	TetR family transcriptional regulator	-6.55	0.0039
AB57_RS16180	AB57_3279	ribonuclease G	-6.55	0.0018
AB57_RS02410	AB57_0460	ferrochelatase	-6.55	0.0050
AB57_RS15745	AB57_3189	hypothetical protein	-6.55	0.0012
AB57_RS14545	AB57_2940	glutamate 5-kinase	-6.55	0.0003
AB57_RS16940	AB57_3431	acyl-CoA thioesterase	-6.55	0.0043
AB57_RS07280	AB57_1449	hypothetical protein	-6.54	0.0030
AB57_RS01440		MULTISPECIES: hypothetical protein	-6.54	0.0024
AB57_RS04480	AB57_0879	aspartate 1-decarboxylase	-6.54	0.0050
AB57_RS01410	AB57_0273	MULTISPECIES: transposase	-6.54	0.0013
AB57_RS19365	AB57_3913	16S rRNA methyltransferase	-6.54	0.0031
AB57_RS14860	AB57_3004	hypothetical protein	-6.54	0.0028
AB57_RS12165	AB57_2453	acetyltransferase	-6.54	0.0016
AB57_RS02605	AB57_0499	hypothetical protein	-6.54	0.0094
AB57_RS03350	AB57_0654	MULTISPECIES: hypothetical protein	-6.53	0.0002
AB57_RS16675	AB57_3378	MULTISPECIES: hypothetical protein	-6.52	0.0020
AB57_RS03625	AB57_0710	exopolyphosphatase	-6.52	0.0002
AB57_RS12455	AB57_2512	DNA helicase	-6.52	0.0003

Table S2. continued.

Locus tag	Locus tag	Function	Log_2 Fitness defect in spleen	*P* value
AB57_RS02370	AB57_0451	apolipoprotein N-acyltransferase	-6.52	0.0030
AB57_RS04705	AB57_0919	alanine acetyltransferase	-6.52	0.0029
AB57_RS16580	AB57_3357	3-hydroxyacyl-CoA dehydrogenase	-6.51	0.0031
AB57_RS16470		formamidopyrimidine-DNA glycosylase	-6.51	0.0094
AB57_RS17570	AB57_3546	hypothetical protein	-6.51	0.0013
AB57_RS01450	AB57_0280	MULTISPECIES: chloramphenicol acetyltransferase CAT	-6.51	0.0005
AB57_RS15730	AB57_3187	integrase	-6.51	0.0034
AB57_RS02445	AB57_0467	hypothetical protein	-6.50	0.0026
AB57_RS13750	AB57_2770	MULTISPECIES: transposase	-6.50	0.0030
AB57_RS03675	AB57_0720	organic hydroperoxide resistance protein	-6.50	0.0002
AB57_RS18425	AB57_3718	permease	-6.50	0.0004
AB57_RS11790	AB57_2373	lactonase	-6.50	0.0020
AB57_RS17540	AB57_3540	acyl-CoA thioesterase II	-6.49	0.0028
AB57_RS12350	AB57_2490	LuxR family transcriptional regulator	-6.49	0.0025
AB57_RS02180	AB57_4843	tRNA-Met	-6.49	0.0046
AB57_RS06725	AB57_1333	tRNA (guanine-N(7)-)-methyltransferase	-6.49	0.0063
AB57_RS18560	AB57_3744	membrane protein	-6.49	0.0042
AB57_RS03885	AB57_0761	N-acetylglutamate synthase	-6.49	0.0012
AB57_RS11900	AB57_2395	universal stress protein	-6.48	0.0094
AB57_RS14540	AB57_2939	phosphoserine phosphatase	-6.48	0.0001
AB57_RS06755	AB57_1339	O-methyltransferase	-6.48	0.0018
AB57_RS01845	AB57_0353	membrane protein	-6.48	0.0010
AB57_RS15555	AB57_3150	MULTISPECIES: 50S ribosomal protein L27	-6.47	0.0044
AB57_RS01560		MULTISPECIES: hypothetical protein	-6.47	0.0015
AB57_RS16960	AB57_3435	hypothetical protein	-6.47	0.0012
AB57_RS19425	AB57_3922	MULTISPECIES: hypothetical protein	-6.47	0.0016
AB57_RS00385	AB57_0074	MULTISPECIES: oxoacyl-ACP reductase	-6.47	0.0024
AB57_RS04055	AB57_0791	membrane protein	-6.46	0.0022
AB57_RS14175	AB57_2856	transporter	-6.46	0.0019
AB57_RS05135	AB57_1010	cobalamin biosynthesis protein	-6.46	0.0001

Table S2. continued.

Locus tag	Locus tag	Function	Log_2 Fitness defect in spleen	*P* value
AB57_RS07505	AB57_1495	D-Ala-D-Ala carboxypeptidase	-6.46	0.0030
AB57_RS02290	AB57_0435	exonuclease V subunit beta	-6.46	0.0000
AB57_RS19220	AB57_3883	hypothetical protein	-6.45	0.0018
AB57_RS00910	AB57_0178	arginine--tRNA ligase	-6.45	0.0032
AB57_RS15590	AB57_3157	AdeB family multidrug efflux RND transporter permease subunit	-6.45	0.0001
AB57_RS16390	AB57_3316	hypothetical protein	-6.45	0.0008
AB57_RS05445	AB57_1076	esterase	-6.45	0.0062
AB57_RS07320	AB57_1457	voltage-gated chloride channel protein	-6.45	0.0064
AB57_RS19185	AB57_3876	4-hydroxy-tetrahydrodipicolinate synthase	-6.45	0.0046
AB57_RS15340	AB57_3102	carbamoyl phosphate synthase large subunit	-6.45	0.0000
AB57_RS02425	AB57_0463	SAM-dependent methyltransferase	-6.44	0.0018
AB57_RS02990	AB57_0578	ligand-gated channel protein	-6.44	0.0022
AB57_RS11330	AB57_2276	hypothetical protein	-6.44	0.0004
AB57_RS01640	AB57_0312	deoxycytidylate deaminase	-6.44	0.0008
AB57_RS15810	AB57_3205	hypothetical protein	-6.44	0.0003
AB57_RS07220	AB57_1434	hypothetical protein	-6.44	0.0008
GenBank:repeat_region:NC_011586:137219:138259			-6.44	0.0051
AB57_RS10695		MULTISPECIES: hypothetical protein	-6.44	0.0002
AB57_RS02350	AB57_0447	type II secretion system protein GspF	-6.43	0.0002
AB57_RS04345	AB57_0850	cell division protein ZipA	-6.43	0.0029
AB57_RS05430	AB57_1073	pyridine nucleotide-disulfide oxidoreductase	-6.43	0.0033
AB57_RS07315	AB57_1456	acetyl/propionyl-CoA carboxylase subuit alpha	-6.43	0.0044
AB57_RS14690	AB57_2968	thioesterase	-6.42	0.0008
AB57_RS07620	AB57_1518	enoyl-CoA hydratase	-6.42	0.0075
AB57_RS03065	AB57_0593	hydrolase	-6.41	0.0054
AB57_RS00805	AB57_0159	porin	-6.41	0.0033
AB57_RS15065	AB57_3049	membrane protein	-6.41	0.0001

Table S2. continued.

Locus tag	Locus tag	Function	Log_2 Fitness defect in spleen	*P* value
AB57_RS11550	AB57_2321	ribosomal protein S12 methylthiotransferase RimO	-6.41	0.0068
AB57_RS14340	AB57_2892	RNA polymerase-associated protein RapA	-6.41	0.0003
AB57_RS00780	AB57_0154	acyl-CoA synthetase	-6.40	0.0056
AB57_RS16135	AB57_3269	PaaX family transcriptional regulator	-6.40	0.0085
AB57_RS19180	AB57_3875	bifunctional pyrazinamidase/nicotinamidase	-6.40	0.0037
AB57_RS18880	AB57_3811	hypothetical protein	-6.40	0.0039
AB57_RS16765	AB57_3396	TonB-dependent receptor	-6.39	0.0071
AB57_RS11220	AB57_2254	potassium transporter TrkH	-6.39	0.0001
AB57_RS08230	AB57_1646	hypothetical protein	-6.38	0.0001
AB57_RS00640	AB57_0127	MULTISPECIES: transposase	-6.38	0.0068
AB57_RS09665	AB57_1942	AraC family transcriptional regulator	-6.38	0.0014
AB57_RS00370	AB57_0071	N-acetylglutamate synthase	-6.38	0.0008
AB57_RS18215	AB57_3675	sulfate permease	-6.37	0.0045
AB57_RS15600	AB57_3159	MULTISPECIES: hypothetical protein	-6.37	0.0002
AB57_RS19450	AB57_3927	MULTISPECIES: hypothetical protein	-6.36	0.0010
AB57_RS18120	AB57_3660	glutamate--tRNA ligase	-6.36	0.0002
AB57_RS16265	AB57_3297	P-nitrobenzoate reductase NfnB	-6.36	0.0002
AB57_RS02980	AB57_0576	pseudogene	-6.36	0.0023
AB57_RS19440	AB57_3925	MULTISPECIES: hypothetical protein	-6.35	0.0006
AB57_RS07175	AB57_1423	GCN5 family N-acetyltransferase	-6.35	0.0008
AB57_RS17755	AB57_3585	N-succinylglutamate 5-semialdehyde dehydrogenase	-6.35	0.0033
AB57_RS14375	AB57_RS14375	pseudogene	-6.35	0.0039
AB57_RS02730	AB57_0525	MULTISPECIES: RNA-binding protein	-6.34	0.0048
AB57_RS02875	AB57_0554	hypothetical protein	-6.34	0.0007
AB57_RS19170	AB57_3873	MATE family efflux transporter	-6.33	0.0022
AB57_RS15560	AB57_3151	MULTISPECIES: 50S ribosomal protein L21	-6.33	0.0062
AB57_RS01800	AB57_0344	RND transporter	-6.33	0.0027
AB57_RS02805	AB57_0540	MULTISPECIES: diacylglycerol kinase	-6.33	0.0069
AB57_RS01360	AB57_0268	MULTISPECIES: transposase	-6.33	0.0015
AB57_RS16340	AB57_3306	bacteriolytic lipoprotein entericidin B	-6.33	0.0002

Table S2. continued.

Locus tag	Locus tag	Function	Log_2 Fitness defect in spleen	*P* value
AB57_RS00905	AB57_0177	cell division protein	-6.33	0.0020
AB57_RS18835	AB57_3802	OHCU decarboxylase	-6.33	0.0048
AB57_RS18330	AB57_3698	hypothetical protein	-6.32	0.0030
AB57_RS16740	AB57_3391	MULTISPECIES: acyl-CoA dehydrogenase	-6.32	0.0000
AB57_RS18295	AB57_3690	MULTISPECIES: membrane protein	-6.32	0.0066
AB57_RS19095	AB57_3858	permease	-6.31	0.0016
AB57_RS14430	AB57_2914	LysR family transcriptional regulator, partial	-6.31	0.0015
AB57_RS15320	AB57_3098	MULTISPECIES: RNA-binding protein	-6.31	0.0002
AB57_RS16845	AB57_4796	tRNA-Leu	-6.31	0.0025
AB57_RS08510	AB57_1704	succinyldiaminopimelate transaminase	-6.31	0.0001
AB57_RS11475	AB57_2306	RNA-binding protein	-6.30	0.0030
AB57_RS05100	AB57_1003	NAD/NADP-dependent betaine aldehyde dehydrogenase	-6.29	0.0001
AB57_RS02505	AB57_0478	ribonuclease E	-6.29	0.0032
AB57_RS15260	AB57_3089	MULTISPECIES: hypothetical protein	-6.28	0.0015
AB57_RS01260	AB57_0248	MULTISPECIES: hypothetical protein	-6.28	0.0004
AB57_RS11350	AB57_2280	hypothetical protein	-6.28	0.0008
AB57_RS05495	AB57_1087	isocitrate lyase	-6.28	0.0016
AB57_RS18315	AB57_3694	hypothetical protein	-6.28	0.0005
GenBank:STS:NC_011586:297629:297780	D13S22		-6.27	0.0014
AB57_RS07550	AB57_1503	transcriptional regulator	-6.27	0.0030
AB57_RS10780		transcriptional regulator	-6.27	0.0014
AB57_RS00715	AB57_0141	alanine glycine permease	-6.27	0.0012
AB57_RS11690	AB57_2351	nitrate reductase	-6.27	0.0001
AB57_RS05725	AB57_1137	peptide transporter	-6.27	0.0013
AB57_RS14305	AB57_2882	hypothetical protein	-6.26	0.0001
AB57_RS14450	AB57_2920	pseudouridine synthase	-6.26	0.0063
AB57_RS06155	AB57_1218	adenine permease	-6.26	0.0034
AB57_RS14005	AB57_2819	acinetobactin biosynthesis protein	-6.25	0.0003

Table S2. continued.

Locus tag	Locus tag	Function	Log$_2$ Fitness defect in spleen	*P* value
AB57_RS00380	AB57_0073	MULTISPECIES: hypothetical protein	-6.25	0.0000
AB57_RS17700	AB57_3574	hypothetical protein	-6.25	0.0016
AB57_RS18280	AB57_3687	MULTISPECIES: 1-(5-phosphoribosyl)-5	-6.25	0.0032
AB57_RS18335	AB57_3699	amino acid transporter LysE	-6.25	0.0041
AB57_RS03045	AB57_0589	permease	-6.24	0.0003
AB57_RS18650	AB57_3763	ArsR family transcriptional regulator	-6.24	0.0046
AB57_RS00890	AB57_0174	quercetin 2,3-dioxygenase	-6.24	0.0028
AB57_RS01575		MULTISPECIES: conjugal transfer protein TrbI	-6.24	0.0008
AB57_RS14200	AB57_2861	hypothetical protein	-6.23	0.0008
AB57_RS19355	AB57_3911	dihydroxy-acid dehydratase	-6.23	0.0007
AB57_RS08880	AB57_1781	serine/threonine transporter SstT	-6.23	0.0009
AB57_RS00465	AB57_0091	tyrosine-protein kinase	-6.23	0.0024
AB57_RS13905	AB57_2798	hypothetical protein	-6.22	0.0006
AB57_RS18870	AB57_3809	DNA topoisomerase IV subunit B	-6.22	0.0008
AB57_RS16855	AB57_3413	membrane protein	-6.22	0.0000
AB57_RS07165	AB57_1421	hypothetical protein	-6.21	0.0050
AB57_RS14255	AB57_2872	phosphate ABC transporter ATP-binding protein	-6.21	0.0049
AB57_RS08485	AB57_1699	aminotransferase AlaT	-6.21	0.0028
AB57_RS15675	AB57_3175	porin	-6.21	0.0025
AB57_RS04280	AB57_0836	hypothetical protein	-6.21	0.0001
AB57_RS02550	AB57_0488	catalase-peroxidase	-6.21	0.0044
AB57_RS01525	AB57_0292	MULTISPECIES: GCN5 family acetyltransferase	-6.20	0.0003
AB57_RS13840	AB57_2785	peptidase M24	-6.20	0.0015
AB57_RS05080	AB57_0999	homocysteine methyltransferase	-6.20	0.0055
AB57_RS02515	AB57_0481	23S rRNA pseudouridine synthase	-6.20	0.0051
AB57_RS03030	AB57_0586	acetate kinase	-6.20	0.0056
AB57_RS14640	AB57_2958	DNA repair protein RecO	-6.19	0.0018
AB57_RS08535	AB57_1709	amino acid transporter	-6.19	0.0030
AB57_RS05160	AB57_1015	poly-beta-1,6 N-acetyl-D-glucosamine synthase	-6.19	0.0035

Table S2. continued.

Locus tag	Locus tag	Function	Log_2 Fitness defect in spleen	*P* value
AB57_RS18565	AB57_3745	beta-aspartyl peptidase	-6.18	0.0001
AB57_RS06650	AB57_1317	aldo-keto reductase	-6.18	0.0030
AB57_RS07250	AB57_1442	type VI secretion system protein	-6.17	0.0021
AB57_RS03485	AB57_0682	23S rRNA (uracil(1939)-C(5))-methyltransferase RlmD	-6.17	0.0014
AB57_RS06940	AB57_1375	HxlR family transcriptional regulator	-6.17	0.0037
AB57_RS07905	AB57_1578	enoyl-CoA hydratase	-6.16	0.0019
AB57_RS04265	AB57_0833	hypothetical protein	-6.16	0.0016
AB57_RS13020	AB57_2623	MULTISPECIES: thiazole synthase	-6.16	0.0040
AB57_RS16945	AB57_3432	thioredoxin	-6.16	0.0008
AB57_RS16540	AB57_3348	MULTISPECIES: quaternary ammonium transporter	-6.16	0.0008
AB57_RS16245	AB57_3292	threonine dehydratase	-6.16	0.0017
AB57_RS16290	AB57_RS16290	pseudogene	-6.15	0.0002
AB57_RS15575	AB57_3154	recombinase	-6.15	0.0014
AB57_RS09000	AB57_1806	MULTISPECIES: TetR family transcriptional regulator	-6.15	0.0051
AB57_RS05940	AB57_1175	alanine glycine permease	-6.15	0.0075
AB57_RS01230	AB57_0242	GntR family transcriptional regulator	-6.14	0.0022
AB57_RS05465	AB57_1081	sulfate adenylyltransferase	-6.14	0.0000
AB57_RS18375	AB57_3707	HxlR family transcriptional regulator	-6.14	0.0006
AB57_RS04990	AB57_0978	membrane protein	-6.14	0.0030
AB57_RS00245	AB57_0050	LysR family transcriptional regulator	-6.14	0.0004
AB57_RS11000	AB57_2208	LysR family transcriptional regulator	-6.13	0.0030
AB57_RS14930	AB57_3019	lipid A biosynthesis acyltransferase	-6.13	0.0017
AB57_RS16550	AB57_3350	sulfite reductase	-6.12	0.0023
AB57_RS16600	AB57_3362	pseudogene	-6.12	0.0004
AB57_RS19350	AB57_3910	hypothetical protein	-6.12	0.0052
AB57_RS19155	AB57_3870	IclR family transcriptional regulator	-6.12	0.0021
AB57_RS15305	AB57_3095	dihydropteroate synthase	-6.12	0.0038
AB57_RS02975	AB57_0575	homoserine O-acetyltransferase	-6.11	0.0000
AB57_RS17040	AB57_3447	signal peptidase	-6.10	0.0035

Table S2. continued.

Locus tag	Locus tag	Function	Log_2 Fitness defect in spleen	*P* value
AB57_RS14425	AB57_2913	MULTISPECIES: L-cystine transporter tcyP	-6.10	0.0008
AB57_RS16840	AB57_3411	two-component system sensor histidine kinase	-6.10	0.0008
AB57_RS19475		flagellar protein	-6.10	0.0001
AB57_RS03680	AB57_RS03680	pseudogene	-6.09	0.0033
AB57_RS15945	AB57_3232	hypothetical protein	-6.09	0.0014
AB57_RS05320	AB57_1048	B12-dependent methionine synthase	-6.08	0.0040
AB57_RS00160	AB57_0033	guanine deaminase	-6.08	0.0015
AB57_RS11025	AB57_2213	MULTISPECIES: hypothetical protein	-6.08	0.0018
AB57_RS16850	AB57_3412	S-adenosylmethionine:tRNA ribosyltransferase-isomerase	-6.08	0.0001
AB57_RS00175	AB57_0036	hypothetical protein	-6.08	0.0052
AB57_RS13010	AB57_2621	Uncharacterized membrane protein YgdD	-6.08	0.0012
AB57_RS01510		MULTISPECIES: transposase	-6.08	0.0004
AB57_RS05710	AB57_1134	chemotaxis protein	-6.07	0.0001
AB57_RS13435	AB57_2708	MULTISPECIES: hypothetical protein	-6.07	0.0029
AB57_RS03050	AB57_0590	gluconokinase	-6.07	0.0022
AB57_RS14935	AB57_3020	pseudogene	-6.07	0.0043
AB57_RS14550	AB57_2941	GTPase Obg	-6.06	0.0049
AB57_RS06000	AB57_1187	MULTISPECIES: GntR family transcriptional regulator	-6.06	0.0009
AB57_RS01010	AB57_0198		-6.06	0.0052
AB57_RS18580	AB57_3748	MULTISPECIES: hypothetical protein	-6.06	0.0004
AB57_RS05705	AB57_1133	NADH pyrophosphatase	-6.06	0.0006
AB57_RS18070	AB57_3650	hypothetical protein	-6.06	0.0045
AB57_RS03135	AB57_0607	membrane protein	-6.05	0.0033
AB57_RS02345	AB57_0446	primosomal protein N\'	-6.05	0.0017
AB57_RS18210	AB57_3674	fatty acyl-CoA reductase	-6.05	0.0008
AB57_RS01635	AB57_0311	MULTISPECIES: NrdR family transcriptional regulator	-6.05	0.0041
AB57_RS14705	AB57_2971	4-amino-4-deoxychorismate lyase	-6.05	0.0010
AB57_RS05105	AB57_1004	MULTISPECIES: BetI family transcriptional regulator	-6.05	0.0026
AB57_RS05800	AB57_1150	MULTISPECIES: dihydroorotase	-6.05	0.0001

Table S2. continued.

Locus tag	Locus tag	Function	Log_2 Fitness defect in spleen	*P* value
AB57_RS05375	AB57_1061	pseudogene	-6.04	0.0001
AB57_RS14265	AB57_2874	phosphate ABC transporter permease	-6.04	0.0044
AB57_RS19445	AB57_3926	MULTISPECIES: hypothetical protein	-6.03	0.0018
AB57_RS14000	AB57_2817	MULTISPECIES: iron ABC transporter permease	-6.03	0.0002
GenBank:repeat_region:NC_011586:263572:269774			-6.03	0.0026
AB57_RS06345	AB57_1256	MULTISPECIES: hypothetical protein	-6.03	0.0036
AB57_RS17280	AB57_3491	membrane protein	-6.03	0.0002
AB57_RS01325	AB57_RS01325	pseudogene	-6.03	0.0007
AB57_RS06765	AB57_1341	alkyl hydroperoxide reductase subunit C	-6.02	0.0016
AB57_RS01300	AB57_0256	pseudogene	-6.02	0.0028
AB57_RS14475	AB57_2925	peptidyl-prolyl cis-trans isomerase	-6.01	0.0023
AB57_RS01055	AB57_0207	hypothetical protein	-6.01	0.0022
AB57_RS06035	AB57_1194	salicylaldehyde dehydrogenase	-6.01	0.0014
AB57_RS04970	AB57_0974	hypothetical protein	-6.01	0.0003
AB57_RS03850	AB57_0754	hydrolase	-6.01	0.0031
AB57_RS14875	AB57_3007	DNA-directed RNA polymerase sigma-70 factor	-6.01	0.0014
AB57_RS01615	AB57_0307	pseudogene	-6.01	0.0028
AB57_RS17070	AB57_3453	starvation protein A	-6.00	0.0006
AB57_RS16885	AB57_3419	phosphopantothenoylcysteine decarboxylase	-6.00	0.0017
AB57_RS18780	AB57_3791	orotate phosphoribosyltransferase	-6.00	0.0000
AB57_RS14855	AB57_3003	peptidoglycan-binding protein	-6.00	0.0002
AB57_RS16285	AB57_3301	membrane protein	-5.99	0.0003
AB57_RS18660	AB57_3766	outer membrane protein	-5.99	0.0000
AB57_RS11650	AB57_2341	HxlR family transcriptional regulator	-5.99	0.0035
AB57_RS14345	AB57_2893	RNA pseudouridine synthase	-5.99	0.0001
AB57_RS17525	AB57_3537	GTP-binding protein	-5.98	0.0053
AB57_RS05625	AB57_1116	cell division protein ZapA	-5.98	0.0006
AB57_RS17750	AB57_3584	N-succinylarginine dihydrolase	-5.98	0.0029

Table S2. continued.

Locus tag	Locus tag	Function	Log_2 Fitness defect in spleen	*P* value
AB57_RS16530	AB57_3346	acetyl-CoA acetyltransferase	-5.98	0.0003
AB57_RS03215	AB57_0623	hypothetical protein	-5.98	0.0000
AB57_RS07555	AB57_1504	hemolysin	-5.97	0.0017
AB57_RS08935	AB57_1792	phosphotransferase	-5.97	0.0048
AB57_RS16500	AB57_RS16500	pseudogene	-5.97	0.0056
AB57_RS14155	AB57_2852	pseudogene	-5.97	0.0044
AB57_RS09965	AB57_2002	S-(hydroxymethyl)glutathione dehydrogenase	-5.97	0.0001
AB57_RS18420	AB57_3716	hypothetical protein	-5.97	0.0010
AB57_RS03795	AB57_0743	protein FilE	-5.97	0.0002
AB57_RS17185	AB57_3473	hypothetical protein	-5.96	0.0030
AB57_RS11845	AB57_2384	hypothetical protein	-5.96	0.0030
AB57_RS04305	AB57_0842	tRNA-dihydrouridine synthase C	-5.96	0.0006
AB57_RS16330	AB57_3304	hypothetical protein	-5.96	0.0006
AB57_RS03300	AB57_0644	integrase	-5.95	0.0012
AB57_RS00660	AB57_0130	short-chain dehydrogenase	-5.95	0.0023
AB57_RS15510	AB57_3141	alcohol dehydrogenase	-5.95	0.0015
AB57_RS17675	AB57_3569	pseudogene	-5.94	0.0084
AB57_RS19375	AB57_3915	SNF family Na+-dependent transporter	-5.94	0.0006
AB57_RS17805	AB57_3596	chromosome partitioning protein ParA	-5.94	0.0014
AB57_RS18520	AB57_3736	membrane protein	-5.94	0.0005
AB57_RS00590	AB57_0117	MULTISPECIES: transcriptional regulator	-5.94	0.0001
AB57_RS18540	AB57_3740	NADPH:quinone oxidoreductase	-5.93	0.0004
AB57_RS03055	AB57_0591	aldehyde dehydrogenase	-5.93	0.0000
AB57_RS03560	AB57_0697	glutathione S-transferase, partial	-5.93	0.0063
AB57_RS09585	AB57_1925	hypothetical protein	-5.93	0.0024
AB57_RS14415	AB57_2911	protease	-5.92	0.0001
AB57_RS09085	AB57_1823	AraC family transcriptional regulator	-5.92	0.0019
AB57_RS16460	AB57_3330	DNA-3-methyladenine glycosylase	-5.92	0.0000
AB57_RS02615	AB57_0501	AraC family transcriptional regulator	-5.92	0.0000
AB57_RS04340	AB57_0849	chromosome segregation protein SMC	-5.91	0.0029

Table S2. continued.

Locus tag	Locus tag	Function	Log_2 Fitness defect in spleen	*P* value
AB57_RS14785	AB57_2989	enoly-CoA hydratase	-5.91	0.0030
AB57_RS04355	AB57_0852	MULTISPECIES: bacterioferritin	-5.91	0.0001
AB57_RS16895	AB57_3422	hypothetical protein	-5.91	0.0008
AB57_RS04415	AB57_0865	hypothetical protein	-5.90	0.0022
AB57_RS14330	AB57_2890	TetR family transcriptional regulator	-5.90	0.0014
AB57_RS00725	AB57_0143	MULTISPECIES: LysR family transcriptional regulator	-5.89	0.0039
AB57_RS17230	AB57_3482	hypothetical protein	-5.89	0.0001
AB57_RS19060	AB57_3851	DNA-cytosine methyltransferase	-5.89	0.0012
AB57_RS03620	AB57_0709	thioredoxin	-5.89	0.0030
AB57_RS18230	AB57_3678	two-component system sensor histidine kinase	-5.89	0.0046
AB57_RS17240	AB57_3484	tryptophan repressor-binding protein	-5.88	0.0003
AB57_RS04180	AB57_0816	LysR family transcriptional regulator	-5.88	0.0032
AB57_RS03945	AB57_0769	tricarballylate utilization protein B	-5.88	0.0030
AB57_RS16050	AB57_3253	pseudogene	-5.88	0.0047
AB57_RS19340	AB57_3908	transcriptional regulator	-5.87	0.0017
AB57_RS12065	AB57_2429	helicase	-5.87	0.0000
AB57_RS11595	AB57_2331	hypothetical protein	-5.87	0.0030
AB57_RS17770	AB57_3588	glutamate dehydrogenase	-5.87	0.0045
AB57_RS03295	AB57_0641	tRNA-dihydrouridine synthase A	-5.87	0.0015
AB57_RS03860	AB57_0756	copper oxidase	-5.86	0.0025
AB57_RS13765	AB57_2773	malic enzyme	-5.86	0.0025
AB57_RS04015	AB57_0783	MULTISPECIES: flavin reductase	-5.86	0.0001
AB57_RS17935	AB57_3623	pilus assembly protein PilW	-5.85	0.0006
AB57_RS00120	AB57_0024	insertase	-5.85	0.0030
AB57_RS14190	AB57_2859	peptidoglycan-binding protein	-5.85	0.0040
AB57_RS16040	AB57_3251	repressor	-5.85	0.0001
AB57_RS16155	AB57_3274	long-chain fatty acid transporter	-5.85	0.0021
AB57_RS00575	AB57_0114	UDP-glucose 4-epimerase	-5.85	0.0046
AB57_RS16900	AB57_3423	phosphoesterase	-5.85	0.0041
AB57_RS02260	AB57_0428	hypothetical protein	-5.83	0.0020

Table S2. continued.

Locus tag	Locus tag	Function	Log_2 Fitness defect in spleen	*P* value
AB57_RS19300	AB57_3900	hypothetical protein	-5.83	0.0044
AB57_RS01520	AB57_0291	MULTISPECIES: aminoglycoside 3-N-acetyltransferase	-5.82	0.0001
AB57_RS05700	AB57_1132	esterase	-5.82	0.0038
AB57_RS03220	AB57_0624	acetolactate synthase	-5.82	0.0030
AB57_RS00460	AB57_0090	peptidyl-prolyl cis-trans isomerase	-5.82	0.0004
AB57_RS04310	AB57_0843	cytochrome C assembly protein	-5.81	0.0005
AB57_RS03750	AB57_0732	UDP-N-acetylglucosamine 1-carboxyvinyltransferase	-5.80	0.0007
AB57_RS16045	AB57_3252	hypothetical protein	-5.80	0.0001
GenBank:repeat_region:NC_011586:3356919:3357108			-5.80	0.0001
AB57_RS11340	AB57_2278	methionine aminopeptidase	-5.80	0.0006
AB57_RS04475	AB57_0878	hypothetical protein	-5.79	0.0030
AB57_RS14920	AB57_3016	hydrolase	-5.78	0.0002
AB57_RS18800	AB57_3795	esterase	-5.78	0.0055
AB57_RS17870	AB57_3610	MULTISPECIES: hemerythrin	-5.78	0.0003
AB57_RS11235	AB57_RS11235	pseudogene	-5.77	0.0057
AB57_RS15480	AB57_3135	MULTISPECIES: hypothetical protein	-5.77	0.0009
AB57_RS08710	AB57_1746	pilus assembly protein	-5.77	0.0039
AB57_RS10690	AB57_2145	TetR family transcriptional regulator	-5.77	0.0003
AB57_RS15660	AB57_3172	two-component system sensor histidine kinase	-5.76	0.0003
AB57_RS12420	AB57_2505	MULTISPECIES: cytochrome O ubiquinol oxidase	-5.76	0.0014
AB57_RS12540	AB57_2530	hydroxyacylglutathione hydrolase	-5.76	0.0030
AB57_RS17810	AB57_3597	membrane protein	-5.76	0.0009
AB57_RS15175	AB57_3071	tRNA 2-thiocytidine biosynthesis protein TtcA	-5.76	0.0028
AB57_RS15245	AB57_3086	glycerol-3-phosphate dehydrogenase	-5.75	0.0040
AB57_RS18190	AB57_3670	hypothetical protein	-5.75	0.0055
AB57_RS18395	AB57_3711	hypothetical protein	-5.74	0.0008
AB57_RS04065	AB57_0793	hypothetical protein	-5.74	0.0003

Table S2. continued.

Locus tag	Locus tag	Function	Log_2 Fitness defect in spleen	*P* value
AB57_RS15815	AB57_3206	hypothetical protein	-5.74	0.0002
AB57_RS18935	AB57_3822	membrane protein	-5.74	0.0043
AB57_RS11885	AB57_2392	hypothetical protein	-5.74	0.0005
AB57_RS07485	AB57_1491	type VI secretion protein	-5.73	0.0018
AB57_RS11185	AB57_2246	hypothetical protein	-5.73	0.0043
AB57_RS08495	AB57_1701	glutathione peroxidase	-5.72	0.0008
AB57_RS02195	AB57_0415	translation initiation factor IF-2	-5.72	0.0051
AB57_RS04955	AB57_0970	hypothetical protein	-5.72	0.0009
AB57_RS14835	AB57_2998	gramicidin S synthase	-5.72	0.0016
AB57_RS14355	AB57_2895	MULTISPECIES: glutamate-1-semialdehyde 2,1-aminomutase	-5.72	0.0030
AB57_RS02270	AB57_0430	hypothetical protein	-5.71	0.0040
AB57_RS01530		MULTISPECIES: hypothetical protein	-5.71	0.0003
AB57_RS15765	AB57_3193	hypothetical protein	-5.71	0.0085
AB57_RS09445	AB57_1895	hypothetical protein	-5.71	0.0001
AB57_RS16735	AB57_3390	acyl-CoA dehydrogenase, partial	-5.71	0.0014
AB57_RS18585	AB57_3749	histidine kinase	-5.71	0.0027
AB57_RS03545	AB57_0694	acyl-CoA synthetase	-5.70	0.0015
AB57_RS18085	AB57_3653	UDP-N-acetylmuramoyl-tripeptide--D-alanyl-D-alanine ligase	-5.70	0.0008
AB57_RS04555	AB57_0890	pseudogene	-5.70	0.0003
AB57_RS04980	AB57_0976	hypothetical protein	-5.70	0.0001
AB57_RS02415	AB57_0462	hypothetical protein	-5.69	0.0021
AB57_RS01215	AB57_0239	hypothetical protein	-5.69	0.0003
AB57_RS03100	AB57_0600	MULTISPECIES: nucleoside diphosphate kinase	-5.69	0.0000
AB57_RS10145	AB57_2034	hypothetical protein	-5.69	0.0008
AB57_RS16595	AB57_3360	GNAT family acetyltransferase	-5.68	0.0001
AB57_RS01870	AB57_0358	type II secretion system protein GspD	-5.68	0.0025
AB57_RS08465	AB57_1695	RNA methyltransferase	-5.68	0.0001
AB57_RS18500	AB57_3732	branched-chain amino acid ABC transporter permease	-5.67	0.0005
AB57_RS18595	AB57_3751	MULTISPECIES: DNA-binding response regulator	-5.67	0.0035

Table S2. continued.

Locus tag	Locus tag	Function	Log_2 Fitness defect in spleen	*P* value
AB57_RS01435		hypothetical protein	-5.67	0.0040
AB57_RS18325	AB57_3696	aquaporin Z	-5.66	0.0030
AB57_RS11850	AB57_2385	LysR family transcriptional regulator	-5.66	0.0030
AB57_RS13510	AB57_2723	replication protein	-5.66	0.0030
AB57_RS13295	AB57_2682	phage tail tape measure protein	-5.66	0.0000
AB57_RS15315	AB57_3097	MULTISPECIES: ribosomal RNA large subunit methyltransferase E	-5.66	0.0040
AB57_RS01400		MULTISPECIES: MerR family transcriptional regulator	-5.66	0.0002
AB57_RS05770		hypothetical protein	-5.66	0.0034
AB57_RS08480	AB57_1698	glutamate symport protein	-5.66	0.0001
AB57_RS00190	AB57_0039	hypothetical protein	-5.65	0.0078
AB57_RS05110	AB57_1005	choline transporter	-5.65	0.0065
AB57_RS18365	AB57_3705	AraC family transcriptional regulator	-5.65	0.0036
AB57_RS14460	AB57_2922	serine protease	-5.64	0.0001
AB57_RS06005	AB57_1188	chlorogenate esterase	-5.64	0.0030
AB57_RS03640	AB57_0713	pyrroline-5-carboxylate reductase	-5.64	0.0017
AB57_RS12525	AB57_2527	hypothetical protein	-5.64	0.0001
AB57_RS16335	AB57_3305	hypothetical protein	-5.63	0.0015
AB57_RS19360	AB57_3912	methionyl-tRNA formyltransferase	-5.63	0.0003
AB57_RS02210	AB57_0418	pesticidal protein Cry5Aa	-5.63	0.0008
AB57_RS15940	AB57_3231	hypothetical protein	-5.63	0.0005
AB57_RS06100	AB57_1207	AraC family transcriptional regulator	-5.62	0.0004
AB57_RS13850	AB57_2787	aminopeptidase N	-5.62	0.0003
AB57_RS13320	AB57_2685	hypothetical protein	-5.62	0.0025
AB57_RS16680	AB57_3379	tryptophan synthase subunit beta	-5.61	0.0026
AB57_RS04300	AB57_0841	hypothetical protein	-5.61	0.0002
AB57_RS01670	AB57_0318	cytosol aminopeptidase	-5.61	0.0033
AB57_RS01840	AB57_0352	hypothetical protein	-5.61	0.0009
AB57_RS14150	AB57_2850	GTPase	-5.61	0.0022
AB57_RS12970	AB57_2613	phosphohistidine phosphatase	-5.61	0.0009

Table S2. continued.

Locus tag	Locus tag	Function	Log_2 Fitness defect in spleen	*P* value
AB57_RS04330	AB57_0847	membrane protein	-5.61	0.0003
AB57_RS14400	AB57_2908	metal-binding protein	-5.60	0.0018
AB57_RS13625	AB57_2746	NADPH-dependent 7-cyano-7-deazaguanine reductase	-5.60	0.0002
AB57_RS09410	AB57_1889	3-deoxy-7-phosphoheptulonate synthase	-5.60	0.0021
AB57_RS03980	AB57_0776	amidohydrolase	-5.60	0.0075
AB57_RS00865	AB57_0169	hypothetical protein	-5.59	0.0000
AB57_RS10630	AB57_2133	GntR family transcriptional regulator	-5.59	0.0003
AB57_RS18635	AB57_3759	16S rRNA methyltransferase	-5.59	0.0038
AB57_RS00545	AB57_0108	UDP-glucose 4-epimerase	-5.58	0.0004
AB57_RS04560	AB57_0891	flavodoxin	-5.58	0.0005
AB57_RS08825	AB57_1770	bifunctional proline dehydrogenase/pyrroline-5-carboxylate dehydrogenase	-5.58	0.0000
AB57_RS11510	AB57_2313	membrane protein	-5.58	0.0001
GenBank:repeat_region:NC_011586:286568:286690			-5.58	0.0034
AB57_RS12290	AB57_2477	kinase	-5.57	0.0004
AB57_RS19100	AB57_3859	histidine ammonia-lyase	-5.57	0.0000
AB57_RS11740	AB57_2361	hypothetical protein	-5.57	0.0015
AB57_RS01160	AB57_0227	MULTISPECIES: hypothetical protein	-5.56	0.0001
AB57_RS18515	AB57_3735	MFS transporter	-5.56	0.0048
AB57_RS14165	AB57_2854	bile acid:sodium symporter	-5.55	0.0062
AB57_RS13080	AB57_2636	hypothetical protein	-5.55	0.0003
AB57_RS19275	AB57_3894	LysR family transcriptional regulator	-5.54	0.0002
AB57_RS19070	AB57_3853	glutamate racemase	-5.54	0.0080
AB57_RS16865	AB57_3415	queuine tRNA-ribosyltransferase	-5.54	0.0000
AB57_RS05845	AB57_1158	type VI secretion system protein	-5.54	0.0034
AB57_RS18440	AB57_4854	tRNA-Thr	-5.54	0.0028
AB57_RS03270	AB57_0635	hydrolase	-5.54	0.0028
AB57_RS19090	AB57_3857	imidazolonepropionase	-5.54	0.0047

Table S2. continued.

Locus tag	Locus tag	Function	Log_2 Fitness defect in spleen	*P* value
AB57_RS04225	AB57_0825	MFS transporter	-5.53	0.0001
AB57_RS04090	AB57_0798	hypothetical protein	-5.53	0.0020
GenBank:STS:NC_011586:736648:737073	D1S3689		-5.53	0.0029
AB57_RS11380	AB57_2287	PTS fructose transporter subunit IIBC	-5.53	0.0010
AB57_RS05575	AB57_1105	endonuclease IV	-5.53	0.0027
AB57_RS19175	AB57_3874	sodium transporter	-5.53	0.0023
AB57_RS18265	AB57_3683	imidazole glycerol phosphate synthase subunit HisH	-5.53	0.0006
AB57_RS13085	AB57_2637	NAD kinase	-5.53	0.0004
AB57_RS10400	AB57_2082	AdeA/AdeI family multidrug efflux RND transporter periplasmic adaptor subunit	-5.52	0.0031
AB57_RS06645	AB57_1316	type I deoxyribonuclease HsdR	-5.52	0.0033
AB57_RS18220	AB57_3676	RNA-binding transcriptional accessory protein	-5.52	0.0027
AB57_RS17175	AB57_3471	oxidoreductase	-5.52	0.0053
AB57_RS19225	AB57_3884	MULTISPECIES: membrane protein	-5.52	0.0006
AB57_RS11490	AB57_2309	hypothetical protein	-5.52	0.0014
AB57_RS18270	AB57_3684	hypothetical protein	-5.52	0.0004
GenBank:repeat_region:NC_011586:283687:285283			-5.51	0.0002
AB57_RS18060	AB57_3648	pilus assembly protein PilM	-5.51	0.0006
AB57_RS09895	AB57_1987	polyphosphate kinase	-5.51	0.0003
AB57_RS08785	AB57_1762	transketolase	-5.51	0.0008
AB57_RS05180	AB57_1019	hypothetical protein	-5.51	0.0014
AB57_RS09550	AB57_1918	pseudogene	-5.50	0.0002
AB57_RS17680	AB57_3570	tetracycline resistance MFS efflux pump	-5.50	0.0008
AB57_RS02835	AB57_0546	phosphatase	-5.50	0.0024
AB57_RS00795	AB57_0157	non-ribosomal peptide synthetase	-5.50	0.0030
AB57_RS04295	AB57_0840	heme utilization protein	-5.49	0.0017
AB57_RS05630	AB57_1117	hypothetical protein	-5.49	0.0003

Table S2. continued.

Locus tag	Locus tag	Function	Log_2 Fitness defect in spleen	*P* value
AB57_RS18005	AB57_3637	hypothetical protein	-5.49	0.0001
AB57_RS17310	AB57_3494	peptidase M3	-5.49	0.0018
AB57_RS08325	AB57_1666	ubiquinol oxidase subunit II, cyanide insensitive	-5.49	0.0069
AB57_RS08895	AB57_1784	cupin	-5.48	0.0026
AB57_RS02870	AB57_0553	hypothetical protein	-5.48	0.0033
AB57_RS05785	AB57_1148	TonB-dependent receptor	-5.48	0.0010
AB57_RS03035	AB57_0587	phosphogluconate dehydratase	-5.47	0.0004
AB57_RS19285	AB57_3897	MFS transporter	-5.47	0.0000
AB57_RS19255	AB57_3890	DNA-3-methyladenine glycosylase	-5.47	0.0000
AB57_RS18160	AB57_3664	membrane protein	-5.47	0.0002
AB57_RS04410	AB57_0864	serine protease	-5.47	0.0036
GenBank:repeat_region:NC_011586:588254:589436			-5.47	0.0002
AB57_RS02880	AB57_0555	MULTISPECIES: transposase	-5.46	0.0002
AB57_RS16715	AB57_3386	hypothetical protein	-5.46	0.0009
AB57_RS00620	AB57_0123	methylcitrate synthase	-5.46	0.0002
AB57_RS06355	AB57_1258	MULTISPECIES: antirepressor	-5.45	0.0046
AB57_RS07805	AB57_1557	carnitine dehydratase	-5.45	0.0023
AB57_RS19115	AB57_3862	histidine utilization repressor	-5.45	0.0003
AB57_RS00130	AB57_0027	transcriptional regulator	-5.45	0.0058
AB57_RS01330	AB57_0262	MULTISPECIES: DNA topoisomerase I	-5.45	0.0017
AB57_RS11170	AB57_2243	membrane protein	-5.44	0.0000
AB57_RS19265	AB57_3892	GntR family transcriptional regulator	-5.44	0.0021
AB57_RS09415	AB57_1890	hypothetical protein	-5.44	0.0036
AB57_RS08885	AB57_1782	ArsR family transcriptional regulator	-5.43	0.0043
AB57_RS03590	AB57_0703	hypothetical protein	-5.43	0.0001
AB57_RS13300		hypothetical protein	-5.43	0.0053
AB57_RS01180	AB57_0231	MFS transporter	-5.42	0.0020
AB57_RS01795	AB57_0343	membrane protein	-5.42	0.0008

Table S2. continued.

Locus tag	Locus tag	Function	Log_2 Fitness defect in spleen	*P* value
AB57_RS18485	AB57_3729	decarboxylase	-5.42	0.0027
AB57_RS03805	AB57_0745	ADP-ribose pyrophosphatase	-5.42	0.0001
AB57_RS17970	AB57_3630	reactive intermediate/imine deaminase	-5.42	0.0030
AB57_RS01630	AB57_0310	ammonia channel protein	-5.42	0.0018
AB57_RS14195	AB57_2860	5\'-nucleotidase surE	-5.42	0.0026
AB57_RS01415	AB57_0275	MULTISPECIES: tetracycline resistance MFS efflux pump	-5.42	0.0004
AB57_RS01790	AB57_0342	transcriptional regulator	-5.41	0.0005
AB57_RS11205	AB57_2251	ribose-5-phosphate isomerase A	-5.41	0.0008
AB57_RS16795	AB57_3402	hypothetical protein	-5.41	0.0005
AB57_RS02580	AB57_0494	3-isopropylmalate dehydratase large subunit	-5.40	0.0000
AB57_RS19040	AB57_3844	bifunctional protein GlmU	-5.40	0.0016
AB57_RS09065	AB57_1819	membrane protein	-5.40	0.0008
AB57_RS19240	AB57_3887	A/G-specific adenine glycosylase	-5.39	0.0013
AB57_RS04700	AB57_0918	metal-dependent hydrolase	-5.39	0.0027
AB57_RS19130	AB57_3865	hypothetical protein	-5.38	0.0001
AB57_RS04565	AB57_0892	MULTISPECIES: SsrA-binding protein	-5.38	0.0017
AB57_RS03970	AB57_0774	MULTISPECIES: LysR family transcriptional regulator	-5.37	0.0003
AB57_RS02100	AB57_0399	secretion protein HlyD	-5.37	0.0018
AB57_RS15570	AB57_3153	solanesyl diphosphate synthase	-5.36	0.0015
AB57_RS03330	AB57_0649	MULTISPECIES: hypothetical protein	-5.36	0.0036
AB57_RS02700	AB57_0519	MarR family transcriptional regulator	-5.36	0.0022
AB57_RS13400	AB57_2701	hypothetical protein	-5.35	0.0051
AB57_RS17285	AB57_3492	hydrolase	-5.35	0.0050
AB57_RS01810	AB57_0346	Fe(2+)-trafficking protein	-5.35	0.0047
AB57_RS14130	AB57_2846	MULTISPECIES: hypothetical protein	-5.35	0.0009
AB57_RS05090	AB57_1001	malate:quinone oxidoreductase	-5.35	0.0003
AB57_RS18810	AB57_3796	thiol:disulfide interchange protein	-5.35	0.0039
AB57_RS00400	AB57_0077	alkali-inducible disulfide interchange protein	-5.35	0.0016
AB57_RS00835	AB57_0163	hypothetical protein	-5.35	0.0014
AB57_RS16125		AraC family transcriptional regulator	-5.34	0.0025

Table S2. continued.

Locus tag	Locus tag	Function	Log_2 Fitness defect in spleen	*P* value
AB57_RS17000	AB57_3439	membrane protein	-5.34	0.0011
AB57_RS00560	AB57_0111	UTP--glucose-1-phosphate uridylyltransferase	-5.34	0.0006
AB57_RS03480	AB57_0681	3\'-5\' exonuclease	-5.34	0.0054
AB57_RS02250	AB57_0426	oxidoreductase	-5.34	0.0007
AB57_RS03275	AB57_0636	aconitate hydratase	-5.34	0.0006
AB57_RS12170	AB57_2452	16S rRNA pseudouridine(516) synthase	-5.34	0.0015
AB57_RS17650	AB57_3563	acyl-CoA dehydrogenase	-5.33	0.0008
GenBank:repeat_region:NC_011586:2911053:2911200			-5.33	0.0023
AB57_RS16560	AB57_3353	porin	-5.33	0.0005
AB57_RS10625	AB57_2132	calcium-binding protein	-5.32	0.0030
AB57_RS19260	AB57_3891	alcohol dehydrogenase	-5.32	0.0039
AB57_RS15395	AB57_3116	neutral zinc metallopeptidase	-5.32	0.0001
AB57_RS14760	AB57_2983	membrane protein	-5.32	0.0027
AB57_RS18610	AB57_3754	transcriptional regulator	-5.32	0.0030
AB57_RS00180	AB57_0037	membrane protein	-5.31	0.0030
AB57_RS18285	AB57_3688	Cro/CI family transcriptional regulator	-5.31	0.0000
AB57_RS13115	AB57_2644	MULTISPECIES: DNA-binding response regulator	-5.31	0.0003
AB57_RS16260	AB57_3296	hypothetical protein	-5.31	0.0005
AB57_RS02965	AB57_0573	hypothetical protein	-5.30	0.0008
AB57_RS10570	AB57_2121	LysR family transcriptional regulator	-5.30	0.0016
AB57_RS00150	AB57_0031	MULTISPECIES: hypoxanthine phosphoribosyltransferase	-5.29	0.0041
AB57_RS06105	AB57_1208	transferase	-5.29	0.0014
AB57_RS00825	AB57_4836	tRNA-Gly	-5.29	0.0030
AB57_RS08125	AB57_1624	lysine transporter LysE	-5.29	0.0030
AB57_RS18355	AB57_4813	tRNA-Ser	-5.28	0.0010
AB57_RS14615	AB57_2954	hypothetical protein	-5.28	0.0030
AB57_RS17215	AB57_3479	endoribonuclease YbeY	-5.28	0.0022

Table S2. continued.

Locus tag	Locus tag	Function	Log_2 Fitness defect in spleen	*P* value
AB57_RS03465	AB57_0677	enoyl-CoA hydratase	-5.27	0.0001
AB57_RS02315	AB57_0440	magnesium transporter	-5.27	0.0017
AB57_RS12545	AB57_2531	MULTISPECIES: hypothetical protein	-5.27	0.0041
AB57_RS03615	AB57_0708	MULTISPECIES: transcription termination factor Rho	-5.27	0.0030
AB57_RS01060	AB57_0208	hypothetical protein	-5.26	0.0001
AB57_RS15010	AB57_3037	DNA gyrase subunit A	-5.26	0.0006
AB57_RS07135	AB57_1415	ATP-binding protein	-5.26	0.0000
AB57_RS02560	AB57_0490	RNA pyrophosphohydrolase	-5.26	0.0001
AB57_RS15690	AB57_3178	esterase	-5.25	0.0048
AB57_RS16065	AB57_3257	hypothetical protein	-5.24	0.0030
AB57_RS04085	AB57_0797	two-component system sensor histidine kinase	-5.24	0.0037
AB57_RS17945	AB57_3625	fimbrial protein	-5.24	0.0006
AB57_RS02500	AB57_0477	long-chain acyl-CoA synthetase	-5.23	0.0008
AB57_RS04425	AB57_0867	pseudogene	-5.22	0.0027
AB57_RS14980	AB57_3031	multidrug ABC transporter permease	-5.21	0.0006
AB57_RS01145	AB57_0223	DNA polymerase IV	-5.21	0.0022
AB57_RS10965	AB57_2200	MarR family transcriptional regulator	-5.21	0.0030
AB57_RS00920	AB57_0180	pseudogene	-5.21	0.0002
AB57_RS17005	AB57_3440	MULTISPECIES: membrane protein	-5.20	0.0002
AB57_RS09905	AB57_1990	MULTISPECIES: pyrroloquinoline-quinone synthase	-5.20	0.0003
AB57_RS14810	AB57_2993	deoxyguanosinetriphosphate triphosphohydrolase	-5.20	0.0025
AB57_RS16685	AB57_3380	N-(5\'-phosphoribosyl)anthranilate isomerase	-5.19	0.0007
AB57_RS06760	AB57_1340	hypothetical protein	-5.19	0.0008
AB57_RS06750	AB57_1338	MULTISPECIES: hypothetical protein	-5.19	0.0043
AB57_RS07160	AB57_1420	O-methyltransferase	-5.18	0.0058
AB57_RS13190	AB57_2660	transcriptional regulator	-5.18	0.0018
AB57_RS11735	AB57_2360	acetyl-CoA carboxylase biotin carboxylase subunit	-5.18	0.0020
AB57_RS18550	AB57_3742	ABC-ATPase UvrA	-5.17	0.0044
AB57_RS05350	AB57_1056	arylsulfatase	-5.16	0.0030
AB57_RS18450	AB57_3722	methyltransferase	-5.15	0.0026

Table S2. continued.

Locus tag	Locus tag	Function	Log_2 Fitness defect in spleen	*P* value
AB57_RS02480	AB57_0474	radical SAM protein	-5.15	0.0053
AB57_RS01980	AB57_0375	sodium:proton antiporter	-5.15	0.0000
AB57_RS16930	AB57_3429	nucleotide-binding protein	-5.15	0.0033
AB57_RS10545	AB57_2114	nicotinate phosphoribosyltransferase	-5.14	0.0015
AB57_RS19455	AB57_3928	MULTISPECIES: hypothetical protein	-5.14	0.0007
AB57_RS05695	AB57_1131	MULTISPECIES: hypothetical protein	-5.14	0.0051
AB57_RS15820	AB57_3207	hypothetical protein	-5.14	0.0006
AB57_RS13870	AB57_2791	MULTISPECIES: hypothetical protein	-5.13	0.0001
AB57_RS18470	AB57_3726	LysR family transcriptional regulator	-5.13	0.0015
AB57_RS16015	AB57_3243	hypothetical protein	-5.13	0.0015
AB57_RS07990	AB57_1595	MFS transporter	-5.13	0.0002
AB57_RS19430	AB57_3923	MULTISPECIES: hypothetical protein	-5.13	0.0010
AB57_RS17015	AB57_3442	phospholipase	-5.13	0.0013
AB57_RS18820	AB57_3799	hypothetical protein	-5.13	0.0046
AB57_RS00610	AB57_0121	MULTISPECIES: GntR family transcriptional regulator	-5.13	0.0003
AB57_RS07025	AB57_1392	hypothetical protein	-5.12	0.0049
AB57_RS00095	AB57_0019	DNA polymerase III subunit beta	-5.12	0.0050
AB57_RS11820	AB57_2379	TetR family transcriptional regulator	-5.11	0.0056
AB57_RS09095	AB57_1825	malate synthase G	-5.11	0.0021
AB57_RS17225	AB57_3481	hypothetical protein	-5.11	0.0001
AB57_RS04600	AB57_0898	betaine/carnitine/choline BCCT transporter	-5.10	0.0015
AB57_RS16450	AB57_3328	oxidoreductase	-5.10	0.0058
AB57_RS17190	AB57_3474	malate dehydrogenase	-5.09	0.0002
AB57_RS03205	AB57_0621	hypothetical protein	-5.09	0.0017
AB57_RS09555	AB57_1919	MULTISPECIES: ATPase AAA	-5.09	0.0016
AB57_RS16775	AB57_3398	MULTISPECIES: aspartate--tRNA(Asp/Asn) ligase	-5.09	0.0030
AB57_RS01580		MULTISPECIES: MerR family transcriptional regulator	-5.09	0.0016
AB57_RS19210	AB57_3881	ATPase AAA	-5.09	0.0002
AB57_RS05690	AB57_1130	MULTISPECIES: hypothetical protein	-5.08	0.0027
AB57_RS14675	AB57_2965	thioesterase	-5.08	0.0016

Table S2. continued.

Locus tag	Locus tag	Function	Log_2 Fitness defect in spleen	*P* value
AB57_RS08455	AB57_1693	hypothetical protein	-5.08	0.0006
AB57_RS15530	AB57_3145	RNA methyltransferase	-5.08	0.0003
AB57_RS12485	AB57_2518	transcriptional regulator	-5.07	0.0035
AB57_RS05435	AB57_1074	MULTISPECIES: rubredoxin	-5.07	0.0042
AB57_RS07910	AB57_1579	nitronate monooxygenase	-5.07	0.0048
AB57_RS00730	AB57_0144	methylmalonate-semialdehyde dehydrogenase	-5.06	0.0001
AB57_RS05850	AB57_1159	type IV secretion protein Rhs	-5.06	0.0000
AB57_RS12190	AB57_2457	hypothetical protein	-5.06	0.0030
AB57_RS03095	AB57_0599	MULTISPECIES: FeS assembly protein IscX	-5.06	0.0056
AB57_RS14135	AB57_2847	carbon starvation protein A	-5.05	0.0013
AB57_RS02525	AB57_0483	hypothetical protein	-5.05	0.0002
AB57_RS04900	AB57_0959	integrase	-5.05	0.0009
AB57_RS12150	AB57_2449	tRNA threonylcarbamoyladenosine biosynthesis protein TsaE	-5.05	0.0051
AB57_RS05075	AB57_0998	arginine:ornithine antiporter	-5.05	0.0005
AB57_RS18995	AB57_3834	hypothetical protein	-5.04	0.0003
AB57_RS06700	AB57_1327	aspartate carbamoyltransferase	-5.04	0.0052
AB57_RS18795	AB57_3794	hypothetical protein	-5.03	0.0017
AB57_RS03410	AB57_0666	MULTISPECIES: hypothetical protein	-5.03	0.0023
AB57_RS03395	AB57_0663	hypothetical protein	-5.03	0.0008
AB57_RS17055	AB57_3450	4-hydroxythreonine-4-phosphate dehydrogenase	-5.03	0.0005
AB57_RS06095	AB57_1206	4-hydroxyacetophenone monooxygenase	-5.03	0.0009
AB57_RS06770	AB57_1342	ATP-dependent helicase	-5.03	0.0000
AB57_RS05555	AB57_1100	penicillin-binding protein 2	-5.03	0.0017
AB57_RS18000	AB57_3636	hypothetical protein	-5.02	0.0036
AB57_RS18320	AB57_3695	AraC family transcriptional regulator	-5.01	0.0042
AB57_RS12775	AB57_2577	hypothetical protein	-5.01	0.0030
AB57_RS09515	AB57_1909	MULTISPECIES: hypothetical protein	-5.01	0.0054
AB57_RS00570	AB57_0113	glucose-6-phosphate isomerase	-5.01	0.0001
AB57_RS19335	AB57_3907	pyrimidine permease	-5.01	0.0003

Table S2. continued.

Locus tag	Locus tag	Function	Log_2 Fitness defect in spleen	*P* value
AB57_RS16505	AB57_3341	lysine transporter	-5.01	0.0058
AB57_RS17220	AB57_3480	cell envelope biogenesis protein TonB	-5.00	0.0001
AB57_RS04400	AB57_0862	ribosomal large subunit pseudouridine synthase B	-5.00	0.0012
AB57_RS18490	AB57_3730	GABA permease	-5.00	0.0039
AB57_RS14685	AB57_2967	L-aspartate oxidase	-5.00	0.0016
AB57_RS00540	AB57_0107	hypothetical protein	-5.00	0.0000
AB57_RS16030	AB57_3247	DNA replication protein DnaD	-5.00	0.0022
AB57_RS14510	AB57_2933	UDP-N-acetyl glucosamine 2-epimerase	-4.99	0.0019
AB57_RS05175	AB57_1018	nucleoside transporter	-4.99	0.0002
AB57_RS18400	AB57_3712	TetR family transcriptional regulator	-4.99	0.0008
AB57_RS19125	AB57_3864	hypothetical protein	-4.98	0.0004
AB57_RS15055	AB57_3046	alanine glycine permease	-4.98	0.0002
AB57_RS16175	AB57_3278	MULTISPECIES: hypothetical protein	-4.98	0.0005
AB57_RS01420	AB57_0276	MULTISPECIES: multidrug transporter	-4.98	0.0027
AB57_RS14850	AB57_3002	translocation protein TolB	-4.98	0.0011
AB57_RS02945	AB57_0569	preprotein translocase subunit TatB	-4.98	0.0041
AB57_RS18555	AB57_3743	diguanylate cyclase	-4.97	0.0018
AB57_RS00405	AB57_0078	TetR family transcriptional regulator	-4.97	0.0015
AB57_RS02595	AB57_0497	3-isopropylmalate dehydrogenase	-4.96	0.0033
AB57_RS18310	AB57_3693	MULTISPECIES: imidazole glycerol phosphate synthase subunit HisF	-4.96	0.0002
AB57_RS15585	AB57_3156	AdeA/AdeI family multidrug efflux RND transporter periplasmic adaptor subunit	-4.96	0.0003
AB57_RS05560	AB57_1101	hypothetical protein	-4.96	0.0013
AB57_RS05440	AB57_1075	ATPase	-4.96	0.0055
AB57_RS15100	AB57_3057	TetR family transcriptional regulator	-4.96	0.0020
AB57_RS07130	AB57_1414	RND transporter	-4.95	0.0077
AB57_RS17795	AB57_3594	hypothetical protein	-4.95	0.0044
AB57_RS04380	AB57_0857	adenosylmethionine--8-amino-7-oxononanoate aminotransferase BioA	-4.95	0.0008

Table S2. continued.

Locus tag	Locus tag	Function	Log_2 Fitness defect in spleen	*P* value
AB57_RS00350	AB57_0067	MULTISPECIES: sulfonate ABC transporter	-4.95	0.0003
AB57_RS18010	AB57_3638	metal-dependent hydrolase	-4.95	0.0001
AB57_RS18135	AB57_3661	pseudogene	-4.94	0.0032
AB57_RS14905	AB57_3013	permease	-4.94	0.0044
AB57_RS17545	AB57_3541	glycerol-3-phosphate acyltransferase	-4.94	0.0030
AB57_RS03955	AB57_0771	LysR family transcriptional regulator	-4.94	0.0036
AB57_RS05660	AB57_1124	MerR family transcriptional regulator	-4.94	0.0030
AB57_RS14500	AB57_2931	hypothetical protein	-4.93	0.0031
AB57_RS16695	AB57_3382	ATP--cobalamin adenosyltransferase	-4.93	0.0014
AB57_RS12780	AB57_2578	LysR family transcriptional regulator	-4.93	0.0039
AB57_RS17020	AB57_3443	acyltransferase	-4.93	0.0002
AB57_RS15705	AB57_3181	hypothetical protein	-4.93	0.0011
AB57_RS04210	AB57_0822	hypothetical protein	-4.92	0.0050
AB57_RS02940		preprotein translocase	-4.92	0.0050
AB57_RS01745	AB57_0333	hypothetical protein	-4.92	0.0003
AB57_RS08970	AB57_1799	ATP-binding protein	-4.92	0.0030
AB57_RS05655	AB57_1123	hypothetical protein	-4.90	0.0002
AB57_RS05585	AB57_1107	glutamate dehydrogenase	-4.89	0.0000
AB57_RS15915	AB57_3226	hypothetical protein	-4.89	0.0030
AB57_RS15060	AB57_3048	DNA repair protein RadA	-4.89	0.0014
AB57_RS04760	AB57_0931	acyltransferase	-4.89	0.0001
AB57_RS02390	AB57_0455	electron transfer flavoprotein-ubiquinone oxidoreductase	-4.89	0.0008
AB57_RS15535	AB57_3146	sirohydrochlorin ferrochelatase	-4.88	0.0014
AB57_RS13065	AB57_2633	acyl-CoA dehydrogenase	-4.88	0.0030
AB57_RS15195	AB57_3075	multidrug efflux RND transporter permease subunit	-4.87	0.0012
AB57_RS00625	AB57_0124	MULTISPECIES: Fe/S-dependent 2-methylisocitrate dehydratase AcnD	-4.87	0.0009
AB57_RS17205	AB57_3477	tRNA-2-methylthio-N(6)-dimethylallyladenosine synthase	-4.87	0.0002
AB57_RS12155	AB57_2450	pseudouridylate synthase	-4.87	0.0050
AB57_RS17085	AB57_3456	3-methylcrotonyl-CoA carboxylase subunit alpha	-4.87	0.0030

Table S2. continued.

Locus tag	Locus tag	Function	Log_2 Fitness defect in spleen	*P* value
AB57_RS06535	AB57_1294	11 methyltransferase	-4.86	0.0003
AB57_RS13535	AB57_2729	hypothetical protein	-4.86	0.0008
AB57_RS02430	AB57_0464	flavoprotein	-4.86	0.0038
AB57_RS14600	AB57_2951	aspartate aminotransferase	-4.85	0.0001
AB57_RS04890	AB57_0957	multidrug resistance protein B	-4.85	0.0008
GenBank:repeat_region:NC_011586:2802623:2802812			-4.84	0.0030
AB57_RS06435	AB57_1272	pseudogene	-4.84	0.0006
AB57_RS01335		MULTISPECIES: hypothetical protein	-4.84	0.0006
AB57_RS03090	AB57_0598	phosphatidylglycerophosphatase	-4.84	0.0004
AB57_RS14975	AB57_3030	putative RND family drug transporter	-4.84	0.0008
AB57_RS16725	AB57_3388	two-component system sensor histidine kinase	-4.83	0.0003
AB57_RS18645	AB57_3762	alkene reductase	-4.83	0.0001
AB57_RS10395	AB57_2081	AdeB family multidrug efflux RND transporter permease subunit	-4.83	0.0002
AB57_RS14630	AB57_2956	hypothetical protein	-4.83	0.0068
AB57_RS13635	AB57_2748	cell wall shape-determining protein	-4.82	0.0017
AB57_RS04395	AB57_0861	dithiobiotin synthetase	-4.82	0.0025
AB57_RS02275	AB57_0431	pseudogene	-4.81	0.0003
AB57_RS13710	AB57_2762	pyridine nucleotide-disulfide oxidoreductase	-4.81	0.0012
AB57_RS05675	AB57_1127	LysR family transcriptional regulator	-4.80	0.0036
AB57_RS19055	AB57_RS19055	pseudogene	-4.80	0.0001
AB57_RS18350	AB57_3703	pseudogene	-4.80	0.0051
AB57_RS06140	AB57_1215	short-chain dehydrogenase	-4.80	0.0030
AB57_RS03930	AB57_0766	acyl-CoA dehydrogenase	-4.80	0.0030
AB57_RS18825	AB57_3800	MULTISPECIES: hypothetical protein	-4.79	0.0017
AB57_RS04190	AB57_0818	hypothetical protein	-4.79	0.0003
AB57_RS11370	AB57_2285	PTS fructose transporter subunit IIA	-4.78	0.0012
AB57_RS06060	AB57_1199	Vanillate O-demethylase oxidoreductase	-4.78	0.0030

Table S2. continued.

Locus tag	Locus tag	Function	Log_2 Fitness defect in spleen	*P* value
AB57_RS00595	AB57_0118	MULTISPECIES: alpha-hydroxy-acid oxidizing enzyme	-4.78	0.0017
AB57_RS03060	AB57_0592	gamma-glutamyl phosphate reductase	-4.78	0.0000
AB57_RS02280	AB57_0433	hypothetical protein	-4.77	0.0011
AB57_RS12340	AB57_2488	peptide chain release factor 1	-4.77	0.0030
AB57_RS17500	AB57_3532	glutamine amidotransferase	-4.77	0.0030
AB57_RS15090	AB57_3054	hypothetical protein	-4.77	0.0017
AB57_RS07420	AB57_1478	hypothetical protein	-4.76	0.0008
AB57_RS15190	AB57_3074	hypothetical protein	-4.76	0.0002
AB57_RS08505	AB57_1703	amino acid transporter	-4.76	0.0030
AB57_RS15285	AB57_3092	alkaline phosphatase	-4.76	0.0025
AB57_RS09135	AB57_1833	heme-binding protein	-4.75	0.0008
AB57_RS01720	AB57_0328	homoserine dehydrogenase	-4.75	0.0001
AB57_RS13480	AB57_2715	hypothetical protein	-4.75	0.0008
AB57_RS13485	AB57_2716	hypothetical protein	-4.75	0.0008
AB57_RS18075	AB57_3651	hypothetical protein	-4.74	0.0030
AB57_RS18145	AB57_4799	tRNA-Glu	-4.74	0.0030
AB57_RS14825	AB57_2996	N-acyl-L-amino acid amidohydrolase	-4.74	0.0003
AB57_RS03320	AB57_0647	hypothetical protein	-4.74	0.0037
AB57_RS17800	AB57_3595	endonuclease	-4.74	0.0001
AB57_RS05155	AB57_1014	poly-beta-1,6-N-acetyl-D-glucosamine N-deacetylase PgaB	-4.74	0.0001
AB57_RS02265	AB57_0429	SAM-dependent methyltransferase	-4.74	0.0069
AB57_RS18170	AB57_3666	cation transporter	-4.74	0.0015
AB57_RS00600	AB57_0119	lactate dehydrogenase	-4.73	0.0013
AB57_RS11855	AB57_2386	transaldolase	-4.73	0.0005
AB57_RS05315	AB57_1047	transketolase, partial	-4.73	0.0030
AB57_RS04385	AB57_0859	pseudogene	-4.73	0.0034
AB57_RS02160	AB57_0411	triosephosphate isomerase	-4.72	0.0003
AB57_RS16690	AB57_3381	ligand-gated channel protein	-4.72	0.0031
AB57_RS02045	AB57_0388	hypothetical protein	-4.72	0.0038
AB57_RS11175	AB57_2244	ATP-dependent Clp protease ATP-binding subunit ClpA	-4.72	0.0022

Table S2. continued.

Locus tag	Locus tag	Function	Log_2 Fitness defect in spleen	*P* value
AB57_RS19135	AB57_3866	amino acid transporter	-4.71	0.0005
AB57_RS11515	AB57_2314	membrane protein	-4.70	0.0038
AB57_RS17555	AB57_3543	nucleoside-diphosphate sugar epimerase	-4.70	0.0093
AB57_RS05985	AB57_1183	glucarate dehydratase	-4.70	0.0030
AB57_RS04960	AB57_0971	hypothetical protein	-4.69	0.0025
AB57_RS03105	AB57_0601	23S rRNA (adenine(2503)-C(2))-methyltransferase RlmN	-4.69	0.0006
AB57_RS01340		MULTISPECIES: hypothetical protein	-4.68	0.0009
AB57_RS09980	AB57_2005	fimbrial protein	-4.68	0.0011
AB57_RS06055	AB57_1198	MULTISPECIES: GntR family transcriptional regulator	-4.68	0.0030
AB57_RS04230	AB57_0826	TetR family transcriptional regulator	-4.67	0.0091
AB57_RS19215	AB57_3882	glucose dehydrogenase	-4.67	0.0007
AB57_RS14470	AB57_2924	NADH dehydrogenase	-4.67	0.0017
AB57_RS14290	AB57_2879	aldehyde dehydrogenase	-4.67	0.0030
GenBank:repeat_region:NC_011586:285536:285658			-4.67	0.0008
AB57_RS10640	AB57_2135	hydrolase	-4.66	0.0030
AB57_RS01995	AB57_0378	heat-shock protein Hsp90	-4.66	0.0010
AB57_RS04070	AB57_0794	ribonucleotide-diphosphate reductase subunit alpha	-4.66	0.0003
AB57_RS02285	AB57_0434	exonuclease V subunit gamma	-4.66	0.0003
AB57_RS01830	AB57_0350	porphobilinogen deaminase	-4.65	0.0008
AB57_RS11570	AB57_2326	ornithine carbamoyltransferase	-4.65	0.0094
AB57_RS18685	AB57_3771	ferredoxin	-4.65	0.0034
AB57_RS19085	AB57_3856	formimidoylglutamase	-4.65	0.0023
AB57_RS15045	AB57_3044	diaminopimelate decarboxylase	-4.65	0.0013
AB57_RS18950	AB57_3825	short-chain dehydrogenase	-4.64	0.0010
AB57_RS14505	AB57_2932	hypothetical protein	-4.64	0.0030
AB57_RS16255	AB57_3294	transporter	-4.64	0.0020
AB57_RS01175	AB57_0230	membrane protein	-4.64	0.0003
AB57_RS18150	AB57_4800	tRNA-Glu	-4.64	0.0030

Table S2. continued.

Locus tag	Locus tag	Function	Log_2 Fitness defect in spleen	*P* value
AB57_RS13755	AB57_2771	MULTISPECIES: transposase	-4.64	0.0090
GenBank:repeat_region:NC_011586:2837509:2838692			-4.64	0.0090
AB57_RS09510	AB57_1908	hypothetical protein	-4.64	0.0030
AB57_RS01000	AB57_0196	MFS transporter	-4.63	0.0032
AB57_RS01020	AB57_0200	pseudogene	-4.63	0.0003
AB57_RS05505	AB57_1089	hypothetical protein	-4.63	0.0017
AB57_RS03290	AB57_0640	hypothetical protein	-4.62	0.0015
AB57_RS18040	AB57_3644	membrane protein	-4.62	0.0000
AB57_RS13415	AB57_2704	hypothetical protein	-4.62	0.0030
AB57_RS14955	AB57_3024	hypothetical protein	-4.62	0.0004
AB57_RS19105	AB57_3860	urocanate hydratase	-4.62	0.0015
AB57_RS14715	AB57_2974	esterase	-4.61	0.0006
AB57_RS16455	AB57_3329	TonB-dependent receptor	-4.61	0.0013
AB57_RS15070	AB57_3050	elongation factor P--(R)-beta-lysine ligase	-4.61	0.0016
AB57_RS18850	AB57_3805	ion channel protein Tsx	-4.60	0.0003
AB57_RS13760	AB57_2772	histidine kinase	-4.60	0.0046
AB57_RS18065	AB57_3649	penicillin-binding protein 1A	-4.60	0.0002
AB57_RS17325	AB57_3497	membrane protein	-4.59	0.0009
AB57_RS04105	AB57_0801	MULTISPECIES: NADH-quinone oxidoreductase subunit B	-4.59	0.0055
AB57_RS09545	AB57_1916	hypothetical protein	-4.59	0.0073
AB57_RS03400	AB57_0664	MULTISPECIES: copper resistance protein CopB	-4.59	0.0005
AB57_RS12785	AB57_2579	polyketide cyclase	-4.58	0.0002
AB57_RS19290	AB57_3898	hypothetical protein	-4.58	0.0003
AB57_RS02115	AB57_0402	DNA repair protein RecN	-4.58	0.0091
AB57_RS03655	AB57_0717	membrane protein	-4.57	0.0030
AB57_RS15400	AB57_3117	membrane protein	-4.57	0.0022
AB57_RS11610	AB57_2334	fumarate hydratase	-4.57	0.0008

Table S2. continued.

Locus tag	Locus tag	Function	Log_2 Fitness defect in spleen	*P* value
AB57_RS03950	AB57_0770	tricarballylate dehydrogenase	-4.57	0.0069
AB57_RS18865	AB57_3808	ureidoglycolate hydrolase	-4.57	0.0009
AB57_RS08835	AB57_1772	proline:sodium symporter	-4.56	0.0042
AB57_RS15715	AB57_3183	3-methylitaconate isomerase	-4.56	0.0002
AB57_RS14950	AB57_3023	hypothetical protein	-4.56	0.0001
AB57_RS19150	AB57_3869	glyoxalase	-4.55	0.0001
AB57_RS00635	AB57_0126	hypothetical protein	-4.55	0.0009
AB57_RS19140	AB57_3867	fumarylacetoacetase	-4.55	0.0028
AB57_RS15375	AB57_RS15375	pseudogene	-4.55	0.0008
AB57_RS03500	AB57_0685	nucleoside triphosphate pyrophosphohydrolase	-4.55	0.0037
AB57_RS01985	AB57_0376	membrane protein	-4.54	0.0026
AB57_RS06630	AB57_1314	ATP phosphoribosyltransferase regulatory subunit	-4.54	0.0007
AB57_RS15485	AB57_3136	MULTISPECIES: hypothetical protein	-4.54	0.0041
AB57_RS01515	AB57_0290	class I integron integrase	-4.52	0.0001
AB57_RS02540	AB57_0486	RpiR family transcriptional regulator	-4.52	0.0043
AB57_RS05275	AB57_1039	AraC family transcriptional regulator	-4.52	0.0037
AB57_RS14765	AB57_2984	ATPase	-4.52	0.0046
AB57_RS09505	AB57_1907	O-succinylhomoserine sulfhydrylase	-4.51	0.0003
AB57_RS14325	AB57_RS14325	pseudogene	-4.51	0.0030
AB57_RS06425	AB57_1271	hypothetical protein	-4.51	0.0030
AB57_RS03585		hypothetical protein	-4.51	0.0005
AB57_RS12465	AB57_2514	peptidase	-4.51	0.0012
AB57_RS15720	AB57_3185	MULTISPECIES: amino acid permease	-4.50	0.0007
AB57_RS01965	AB57_0372	hypothetical protein	-4.50	0.0027
AB57_RS19295	AB57_3899	4-hydroxy-tetrahydrodipicolinate reductase	-4.49	0.0073
AB57_RS18915	AB57_3818	dihydrolipoamide acetyltransferase	-4.49	0.0007
AB57_RS05960	AB57_1178	coniferyl aldehyde dehydrogenase	-4.49	0.0016
AB57_RS00710	AB57_0140	MULTISPECIES: hypothetical protein	-4.48	0.0030
AB57_RS14410	AB57_2910	hypothetical protein	-4.48	0.0005
AB57_RS04660	AB57_0910	hypothetical protein	-4.46	0.0015

Table S2. continued.

Locus tag	Locus tag	Function	Log_2 Fitness defect in spleen	*P* value
AB57_RS01740	AB57_0332	iron transporter FeoB	-4.46	0.0024
AB57_RS05805	AB57_1151	diguanylate cyclase	-4.46	0.0001
AB57_RS16790	AB57_3401	galactosyltransferase Lgt5	-4.44	0.0038
AB57_RS17245	AB57_3485	membrane protein	-4.44	0.0006
AB57_RS11840	AB57_2383	MULTISPECIES: acetyl-CoA acetyltransferase	-4.43	0.0006
AB57_RS07270	AB57_1447	type IV secretion protein Rhs	-4.43	0.0030
AB57_RS00695	AB57_0137	MULTISPECIES: AsnC family transcriptional regulator	-4.43	0.0050
AB57_RS10140	AB57_2033	hypothetical protein	-4.43	0.0004
AB57_RS13530	AB57_2728	alkaline phosphatase	-4.43	0.0030
AB57_RS02520	AB57_0482	phosphoglycolate phosphatase	-4.41	0.0048
AB57_RS11225	AB57_2255	phospholipase	-4.41	0.0002
AB57_RS05720	AB57_1136	1,4-beta-N-acetylmuramidase	-4.41	0.0014
AB57_RS04895	AB57_0958	gamma-glutamyltransferase	-4.40	0.0039
AB57_RS15290	AB57_4795	tRNA-Leu	-4.39	0.0029
GenBank:repeat_region:NC_011586:3201913:3202051			-4.39	0.0029
AB57_RS01355	AB57_0267	MULTISPECIES: acetyltransferase	-4.39	0.0030
AB57_RS18855	AB57_3806	monooxygenase	-4.39	0.0025
AB57_RS00430	AB57_0084	phospholipase C	-4.39	0.0042
GenBank:repeat_region:NC_011586:593000:599202			-4.39	0.0008
GenBank:STS:NC_011586:297629:297780	D13S22		-4.38	0.0030
AB57_RS06380	AB57_1262	hypothetical protein	-4.38	0.0030
AB57_RS14285	AB57_2878	MULTISPECIES: AsnC family transcriptional regulator	-4.37	0.0030
AB57_RS17195	AB57_3475	hypothetical protein	-4.37	0.0035
AB57_RS17260	AB57_3488	fumarylacetoacetate hydrolase	-4.36	0.0039

Table S2. continued.

Locus tag	Locus tag	Function	Log_2 Fitness defect in spleen	*P* value
AB57_RS03335	AB57_0651	MULTISPECIES: molecular chaperone DnaJ	-4.36	0.0024
AB57_RS10375	AB57_2077	transcriptional regulator	-4.35	0.0010
AB57_RS03870	AB57_0758	cation transporter	-4.35	0.0001
AB57_RS00450	AB57_0088	membrane protein	-4.34	0.0030
AB57_RS14310	AB57_2883	TetR family transcriptional regulator	-4.34	0.0015
AB57_RS06815	AB57_1351	MULTISPECIES: LysR family transcriptional regulator	-4.33	0.0029
AB57_RS02150	AB57_0409	type II secretion system protein F	-4.33	0.0024
AB57_RS15580	AB57_3155	phosphatidylglycerophosphatase	-4.32	0.0031
AB57_RS04110	AB57_0802	NADH-quinone oxidoreductase subunit C/D	-4.32	0.0055
AB57_RS07155	AB57_1419	universal stress protein	-4.32	0.0077
AB57_RS08435	AB57_1689	chromate transporter	-4.32	0.0003
AB57_RS04985	AB57_0977	TatD family hydrolase	-4.32	0.0011
AB57_RS07490	AB57_1492	type VI secretion protein	-4.32	0.0045
AB57_RS00445	AB57_0087	MULTISPECIES: N-acetyl-anhydromuranmyl-L-alanine amidase	-4.31	0.0033
GenBank:repeat_region:NC_011586:324399:326560			-4.30	0.0006
AB57_RS16535	AB57_3347	3-demethylubiquinone-9 3-methyltransferase	-4.30	0.0036
AB57_RS15700	AB57_3180	membrane protein	-4.29	0.0062
AB57_RS18785	AB57_3792	catabolite repression control protein	-4.29	0.0038
AB57_RS05915	AB57_1170	polyketide cyclase	-4.29	0.0030
AB57_RS03230	AB57_0626	MULTISPECIES: ketol-acid reductoisomerase	-4.29	0.0005
AB57_RS03130	AB57_0606	hypothetical protein	-4.28	0.0014
AB57_RS15825	AB57_3208	MULTISPECIES: DNA-binding protein	-4.28	0.0035
AB57_RS19320	AB57_3904	RND transporter	-4.28	0.0012
AB57_RS04965	AB57_RS04965	pseudogene	-4.27	0.0008
AB57_RS14960	AB57_3025	transporter	-4.27	0.0005
AB57_RS13280	AB57_2679	hypothetical protein	-4.26	0.0025
AB57_RS13305	AB57_2683	hypothetical protein	-4.25	0.0018

Table S2. continued.

Locus tag	Locus tag	Function	Log_2 Fitness defect in spleen	*P* value
AB57_RS00140	AB57_0029	thiol:disulfide interchange protein	-4.25	0.0000
AB57_RS03865	AB57_0757	copper resistance protein B	-4.25	0.0014
AB57_RS06310	AB57_1249	MULTISPECIES: hypothetical protein	-4.25	0.0030
AB57_RS15205	AB57_3077	hydrolase	-4.24	0.0002
AB57_RS12055	AB57_2427	hydroxyethylthiazole kinase	-4.23	0.0002
AB57_RS05235	AB57_1030	MULTISPECIES: short-chain dehydrogenase	-4.23	0.0077
AB57_RS15795	AB57_3202	DNA-binding protein	-4.22	0.0030
AB57_RS14985	AB57_3032	multidrug ABC transporter permease	-4.22	0.0025
AB57_RS10655	AB57_RS10655	pseudogene	-4.22	0.0003
AB57_RS02000	AB57_0379	selenocysteine synthase	-4.22	0.0002
AB57_RS15095	AB57_3056	pseudogene	-4.22	0.0017
AB57_RS09520	AB57_1910	MULTISPECIES: recombinase RecR	-4.21	0.0034
AB57_RS05420	AB57_1071	LysR family transcriptional regulator	-4.21	0.0046
AB57_RS16365	AB57_3311	two-component system sensor histidine kinase/response regulator	-4.20	0.0032
AB57_RS05590	AB57_1108	hypothetical protein	-4.19	0.0006
AB57_RS11240	AB57_2259	sugar kinase	-4.19	0.0040
AB57_RS03455	AB57_0675	hydroxypyruvate isomerase	-4.19	0.0004
AB57_RS02325	AB57_0442	DNA-binding protein	-4.18	0.0005
AB57_RS02245	AB57_0425	phosphoribosyl-ATP diphosphatase	-4.17	0.0038
AB57_RS03690	AB57_0724	carbon-nitrogen hydrolase	-4.17	0.0006
AB57_RS16515	AB57_3343	hypothetical protein	-4.17	0.0009
AB57_RS16375	AB57_3313	twitching motility protein PilT	-4.16	0.0025
AB57_RS19000	AB57_3835	hypothetical protein	-4.16	0.0049
AB57_RS16565	AB57_3354	carnitine dehydratase	-4.16	0.0004
AB57_RS05325	AB57_1050	nuclease PIN	-4.15	0.0037
AB57_RS05760		hypothetical protein	-4.14	0.0077
AB57_RS11195	AB57_2250	hypothetical protein	-4.14	0.0030
AB57_RS12040	AB57_2424	aminopeptidase N	-4.14	0.0001
AB57_RS09590	AB57_1926	MULTISPECIES: transcriptional regulator, partial	-4.14	0.0030

Table S2. continued.

Locus tag	Locus tag	Function	Log_2 Fitness defect in spleen	*P* value
AB57_RS01130	AB57_0220	radical SAM protein	-4.13	0.0023
AB57_RS16385	AB57_3315	MULTISPECIES: transcriptional regulator	-4.12	0.0049
AB57_RS08730	AB57_1750	biotin synthase	-4.11	0.0010
AB57_RS03565	AB57_0698	hypothetical protein	-4.10	0.0053
AB57_RS05070	AB57_RS05070	pseudogene	-4.09	0.0008
AB57_RS19305	AB57_3901	MULTISPECIES: molecular chaperone DnaJ	-4.09	0.0011
AB57_RS00525	AB57_0104	UDP-glucose 4-epimerase	-4.08	0.0001
AB57_RS12295	AB57_2478	molybdopterin biosynthesis protein	-4.08	0.0030
AB57_RS01275	AB57_0251	MULTISPECIES: FMN reductase	-4.07	0.0008
AB57_RS05525	AB57_1094	urease subunit alpha	-4.07	0.0030
AB57_RS08540	AB57_1710	hypothetical protein	-4.07	0.0017
AB57_RS01170	AB57_0229	preprotein translocase SecA	-4.06	0.0000
AB57_RS02650	AB57_0509	proton glutamate symport protein	-4.06	0.0001
AB57_RS16395	AB57_3317	hemolysin D	-4.06	0.0000
AB57_RS17210	AB57_3478	ATP-binding protein	-4.05	0.0001
AB57_RS14845	AB57_3000	pseudogene	-4.04	0.0022
AB57_RS05450	AB57_1077	lysine--tRNA ligase	-4.04	0.0069
AB57_RS14250	AB57_2871	peptidase S49	-4.04	0.0041
AB57_RS14585	AB57_2948	hypothetical protein	-4.04	0.0037
AB57_RS00850	AB57_0166	threonine transporter RhtB	-4.03	0.0030
AB57_RS16270	AB57_3298	Bcr/CflA family drug resistance efflux transporter	-4.02	0.0004
AB57_RS09735	AB57_1955	aspartate ammonia-lyase	-4.02	0.0010
AB57_RS13100	AB57_2641	hypothetical protein	-4.02	0.0008
AB57_RS00270	AB57_0055	peptidyl-prolyl cis-trans isomerase	-4.02	0.0002
AB57_RS01610	AB57_0306	hypothetical protein	-4.01	0.0014
GenBank:repeat_region:NC_011586:319168:322540			-4.01	0.0003
AB57_RS03820	AB57_0748	tRNA threonylcarbamoyladenosine biosynthesis protein TsaB	-4.01	0.0077

Table S2. continued.

Locus tag	Locus tag	Function	Log$_2$ Fitness defect in spleen	*P* value
AB57_RS17930	AB57_3622	type IV fimbrial biogenesis protein	-4.01	0.0049
AB57_RS08545	AB57_1711	hypothetical protein	-4.00	0.0009
AB57_RS16220	AB57_3287	glutamine amidotransferase	-3.99	0.0020
AB57_RS17640	AB57_3561	shikimate dehydrogenase (NADP+)	-3.99	0.0008
AB57_RS18700	AB57_3774	membrane protein	-3.99	0.0010
AB57_RS05240	AB57_1031	MULTISPECIES: L-aspartate dehydrogenase	-3.98	0.0091
GenBank:repeat_region:NC_011586:2264424:2265754			-3.98	0.0069
AB57_RS00135	AB57_0028	cation diffusion facilitator transporter	-3.98	0.0003
AB57_RS03890	AB57_0762	MULTISPECIES: quinolinate synthetase	-3.98	0.0002
AB57_RS03225	AB57_0625	MULTISPECIES: acetolactate synthase small subunit	-3.98	0.0029
AB57_RS00090	AB57_0018	DNA recombination protein RecF	-3.98	0.0000
AB57_RS10875	AB57_2182	3-oxoadipate CoA-transferase subunit A	-3.98	0.0069
AB57_RS16150	AB57_3273	NAD(P)H dehydrogenase	-3.97	0.0004
AB57_RS02230	AB57_0422	cation:proton antiporter	-3.96	0.0008
AB57_RS01880	AB57_0360	phosphoglycolate phosphatase	-3.95	0.0017
AB57_RS16830	AB57_3409	MULTISPECIES: branched-chain amino acid aminotransferase	-3.95	0.0033
AB57_RS04285	AB57_RS04285	pseudogene	-3.94	0.0020
AB57_RS10700	AB57_2147	N-acyl-L-amino acid amidohydrolase	-3.94	0.0008
AB57_RS12530	AB57_2528	methionine ABC transporter ATP-binding protein	-3.94	0.0084
AB57_RS07005	AB57_1388	helicase	-3.94	0.0005
AB57_RS09030	AB57_1812	uracil-DNA glycosylase	-3.93	0.0006
AB57_RS07255	AB57_1443	type IV secretion protein Rhs	-3.93	0.0037
AB57_RS00200	AB57_0041	membrane protein	-3.93	0.0004
AB57_RS16935	AB57_3430	hypothetical protein	-3.90	0.0022
AB57_RS02175	AB57_4842	tRNA-Met	-3.89	0.0017
AB57_RS18055	AB57_3647	membrane protein	-3.89	0.0010
AB57_RS01885	AB57_0361	anthranilate synthase	-3.89	0.0010

Table S2. continued.

Locus tag	Locus tag	Function	Log_2 Fitness defect in spleen	*P* value
AB57_RS15360	AB57_3106	methyltransferase	-3.88	0.0055
AB57_RS16410	AB57_3320	hypothetical protein	-3.88	0.0055
AB57_RS05810	AB57_1152	argininosuccinate synthase	-3.88	0.0038
AB57_RS18890	AB57_3813	hypothetical protein	-3.87	0.0013
AB57_RS05270	AB57_1038	glutaminase	-3.87	0.0018
AB57_RS18015	AB57_3639	glutamate synthase subunit beta	-3.87	0.0006
AB57_RS04800	AB57_0939	MULTISPECIES: hemerythrin	-3.86	0.0008
AB57_RS13970	AB57_2811	peptide synthetase	-3.86	0.0030
AB57_RS11245	AB57_2260	Rieske	-3.86	0.0015
AB57_RS14515	AB57_2934	hypothetical protein	-3.86	0.0077
AB57_RS15145	AB57_3066	peptide synthetase	-3.86	0.0030
AB57_RS19015	AB57_3838	3,4-dihydroxy-2-butanone-4-phosphate synthase	-3.85	0.0011
AB57_RS15785	AB57_3197	tail protein	-3.84	0.0014
AB57_RS02740	AB57_0527	hypothetical protein	-3.84	0.0043
AB57_RS19050	AB57_RS19050	pseudogene	-3.83	0.0034
AB57_RS04715	AB57_0921	leucyl/phenylalanyl-tRNA--protein transferase	-3.83	0.0024
AB57_RS00565	AB57_0112	UDP-glucose 6-dehydrogenase	-3.83	0.0055
AB57_RS15565		hypothetical protein	-3.82	0.0030
AB57_RS05670	AB57_1126	amino-acid transporter Mb2008	-3.82	0.0003
AB57_RS18675	AB57_3769	phosphoglucosamine mutase	-3.82	0.0047
AB57_RS17255	AB57_3487	AraC family transcriptional regulator	-3.82	0.0008
AB57_RS16160	AB57_3275	phosphoserine phosphatase	-3.80	0.0007
AB57_RS13110	AB57_2643	two-component system sensor histidine kinase	-3.80	0.0055
AB57_RS02710	AB57_0521	DNA topoisomerase I	-3.80	0.0022
AB57_RS14025	AB57_RS14025	pseudogene	-3.79	0.0055
AB57_RS14665	AB57_2963	S26 family signal peptidase	-3.79	0.0030
AB57_RS06320	AB57_1251	crossover junction endodeoxyribonuclease RusA	-3.79	0.0030
AB57_RS02685	AB57_0516	pseudogene	-3.79	0.0016

Table S2. continued.

Locus tag	Locus tag	Function	Log_2 Fitness defect in spleen	*P* value
AB57_RS08905	AB57_1786	hypothetical protein	-3.78	0.0002
AB57_RS18455	AB57_3723	hypothetical protein	-3.78	0.0001
AB57_RS02725	AB57_0524	ATP-dependent DNA helicase PcrA	-3.77	0.0026
AB57_RS18235	AB57_3679	acetyl-CoA hydrolase	-3.76	0.0006
AB57_RS00705	AB57_0139	alanine racemase	-3.75	0.0031
AB57_RS02320	AB57_0441	MULTISPECIES: MerR family transcriptional regulator	-3.75	0.0016
AB57_RS06660	AB57_1319	MULTISPECIES: osmotically inducible protein C	-3.75	0.0047
AB57_RS06335	AB57_1254	MULTISPECIES: hypothetical protein	-3.75	0.0030
AB57_RS05050	AB57_0991	heme oxygenase	-3.74	0.0030
AB57_RS02530	AB57_0484	glutathione S-transferase, partial	-3.74	0.0055
AB57_RS13975	AB57_2812	Rhizobactin siderophore biosynthesis protein rhbE	-3.73	0.0030
AB57_RS00760	AB57_0150	MFS transporter	-3.73	0.0017
AB57_RS01155	AB57_0226	MFS transporter	-3.72	0.0040
AB57_RS17515	AB57_3535	flavohemoprotein	-3.71	0.0026
AB57_RS01405		MULTISPECIES: transposase	-3.71	0.0003
AB57_RS11180	AB57_2245	MULTISPECIES: ATP-dependent Clp protease adaptor ClpS	-3.70	0.0081
AB57_RS08460	AB57_1694	MULTISPECIES: serine O-acetyltransferase	-3.68	0.0013
AB57_RS17875	AB57_3611	membrane protein	-3.68	0.0029
AB57_RS17250	AB57_3486	membrane protein	-3.68	0.0042
AB57_RS13695	AB57_2759	MFS transporter	-3.66	0.0030
AB57_RS14280	AB57_2877	pyruvate decarboxylase	-3.66	0.0070
AB57_RS01605	AB57_0305	MULTISPECIES: sulfate transporter	-3.65	0.0058
AB57_RS17940	AB57_3624	hypothetical protein	-3.65	0.0037
AB57_RS06675	AB57_1322	chaperone protein ClpB	-3.65	0.0010
AB57_RS06300	AB57_1247	hypothetical protein	-3.65	0.0029
AB57_RS13865	AB57_2790	indole-3-glycerol phosphate synthase	-3.63	0.0026
AB57_RS15710	AB57_3182	MULTISPECIES: geranyltranstransferase	-3.61	0.0001

Table S2. continued.

Locus tag	Locus tag	Function	Log$_2$ Fitness defect in spleen	*P* value
AB57_RS14580	AB57_2947	excinuclease ABC subunit B	-3.61	0.0004
AB57_RS14795		hypothetical protein	-3.61	0.0030
AB57_RS19315	AB57_3903	ACR family transporter	-3.61	0.0002
AB57_RS00700	AB57_0138	MULTISPECIES: D-amino acid dehydrogenase	-3.61	0.0004
AB57_RS15475	AB57_3134	hypothetical protein	-3.60	0.0065
AB57_RS18430	AB57_3717	LysR family transcriptional regulator	-3.60	0.0021
AB57_RS17760	AB57_3586	arginine N-succinyltransferase	-3.59	0.0013
AB57_RS16085	AB57_3260	hypothetical protein	-3.58	0.0030
AB57_RS16710	AB57_3385	fatty acid desaturase	-3.58	0.0007
AB57_RS00605	AB57_0120	MULTISPECIES: aromatic amino acid aminotransferase	-3.58	0.0013
AB57_RS08330	AB57_1667	cytochrome D ubiquinol oxidase subunit I	-3.57	0.0030
AB57_RS03110	AB57_0602	pilus assembly protein PilW	-3.55	0.0000
AB57_RS02925	AB57_0564	MULTISPECIES: transposase	-3.55	0.0013
AB57_RS02765	AB57_0532	coniferyl aldehyde dehydrogenase	-3.55	0.0022
AB57_RS12960	AB57_2610	Type II secretion system	-3.55	0.0002
AB57_RS13285	AB57_2680	hypothetical protein	-3.55	0.0049
AB57_RS13125	AB57_2646	type II secretion system protein E	-3.54	0.0069
AB57_RS08740	AB57_1752	hypothetical protein	-3.54	0.0069
AB57_RS05265	AB57_1037	peptide deformylase	-3.54	0.0036
AB57_RS11520	AB57_2315	peptidase	-3.53	0.0008
AB57_RS14790	AB57_2988	NIF3 1	-3.53	0.0030
AB57_RS14730	AB57_2977	sulfate ABC transporter ATP-binding protein	-3.53	0.0001
AB57_RS02420	AB57_0461	MULTISPECIES: DNA gyrase inhibitor	-3.53	0.0013
AB57_RS01035	AB57_0203	peptidoglycan-binding protein LysM	-3.51	0.0021
AB57_RS13215	AB57_2667	hypothetical protein	-3.50	0.0015
AB57_RS06305	AB57_1248	DNA replication protein	-3.49	0.0015
AB57_RS18045	AB57_3645	hypothetical protein	-3.47	0.0049
AB57_RS11525	AB57_2316	1-deoxy-D-xylulose 5-phosphate reductoisomerase	-3.47	0.0019
AB57_RS13920	AB57_2801	peptidase	-3.47	0.0027

Table S2. continued.

Locus tag	Locus tag	Function	Log_2 Fitness defect in spleen	*P* value
AB57_RS14045	AB57_2829	hypothetical protein	-3.46	0.0014
AB57_RS06330	AB57_1253	hypothetical protein	-3.46	0.0069
AB57_RS09350	AB57_1876	ligand-gated channel protein	-3.43	0.0014
AB57_RS02860	AB57_0551	MULTISPECIES: carbapenem-hydrolyzing class D beta-lactamase OXA-23	-3.42	0.0016
AB57_RS14040	AB57_2828	hypothetical protein	-3.41	0.0006
AB57_RS05400	AB57_1067	peptidase S54	-3.40	0.0016
AB57_RS05615	AB57_1113	hypothetical protein	-3.39	0.0057
AB57_RS17530	AB57_3538	metal-dependent hydrolase	-3.39	0.0022
AB57_RS07435	AB57_1481	MULTISPECIES: hypothetical protein	-3.38	0.0030
AB57_RS18245	AB57_4839	tRNA-Phe	-3.36	0.0030
AB57_RS01305		MULTISPECIES: MerR family transcriptional regulator	-3.33	0.0055
AB57_RS05855	AB57_1160	hypothetical protein	-3.33	0.0022
AB57_RS01725	AB57_0329	thiol:disulfide interchange protein	-3.33	0.0014
AB57_RS05125	AB57_1008	MULTISPECIES: acyl-CoA thioesterase	-3.33	0.0000
AB57_RS17235	AB57_3483	xanthine phosphoribosyltransferase	-3.32	0.0005
AB57_RS15430	AB57_3124	dihydrolipoamide dehydrogenase	-3.31	0.0077
AB57_RS19110	AB57_3861	hypothetical protein	-3.31	0.0006
AB57_RS08005	AB57_1598	3-methylcrotonyl-CoA carboxylase subunit alpha	-3.31	0.0006
AB57_RS04755	AB57_0930	preprotein translocase subunit SecA	-3.31	0.0038
AB57_RS08850	AB57_1775	AraC family transcriptional regulator	-3.29	0.0050
AB57_RS18275	AB57_3685	pseudogene	-3.28	0.0055
AB57_RS04095	AB57_0799	diguanylate cyclase	-3.28	0.0002
AB57_RS07770	AB57_1550	cytochrome B561	-3.28	0.0033
AB57_RS17980	AB57_3632	MULTISPECIES: bacterioferritin	-3.26	0.0033
AB57_RS18600	AB57_3752	FMN reductase	-3.26	0.0049
AB57_RS02155	AB57_0410	type IV-A pilus assembly ATPase PilB	-3.25	0.0001

Table S2. continued.

Locus tag	Locus tag	Function	Log_2 Fitness defect in spleen	*P* value
AB57_RS17920	AB57_3620	pilin	-3.23	0.0005
AB57_RS04270	AB57_0834	multidrug transporter MatE	-3.23	0.0015
AB57_RS02985	AB57_0577	MULTISPECIES: hydroxylase	-3.20	0.0000
AB57_RS17765	AB57_3587	acetylornithine aminotransferase	-3.20	0.0011
AB57_RS12980	AB57_2615	Arsenite_oxidase; nitroreductase	-3.20	0.0017
AB57_RS15050	AB57_3045	MULTISPECIES: hypothetical protein	-3.19	0.0003
AB57_RS03460	AB57_0676	2-hydroxy-3-oxopropionate reductase	-3.19	0.0001
AB57_RS02625	AB57_0503	L-asparaginase 1	-3.16	0.0049
AB57_RS02065	AB57_0392	acyl-CoA thioesterase	-3.15	0.0003
AB57_RS02735	AB57_0526	RNA-binding protein	-3.13	0.0015
AB57_RS07460	AB57_1486	type VI secretion protein	-3.11	0.0030
AB57_RS09970	AB57_2003	hypothetical protein	-3.10	0.0030
AB57_RS17520	AB57_3536	MULTISPECIES: hypothetical protein	-3.10	0.0015
AB57_RS18290	AB57_3689	transporter	-3.09	0.0033
AB57_RS14095	AB57_2840	aldehyde dehydrogenase	-3.08	0.0030
AB57_RS16995	AB57_3438	hypothetical protein	-3.07	0.0046
AB57_RS03145	AB57_0609	ACP synthase	-3.06	0.0008
AB57_RS02935	AB57_0567	alkaline phosphatase	-3.05	0.0033
AB57_RS17925	AB57_3621	pilus assembly protein PilY	-3.03	0.0034
AB57_RS11560	AB57_2324	nitrogen regulation protein NR(I)	-3.02	0.0052
AB57_RS15225	AB57_3081	pseudogene	-2.99	0.0073
AB57_RS11555	AB57_2323	PAS domain-containing sensor histidine kinase	-2.99	0.0003
AB57_RS09425	AB57_1891	peptidase	-2.99	0.0014
AB57_RS14740	AB57_2979	membrane protein	-2.92	0.0009
AB57_RS02040	AB57_0387	fatty acid oxidation complex subunit alpha	-2.91	0.0003
AB57_RS08890	AB57_1783	TetR family transcriptional regulator	-2.90	0.0005
AB57_RS02555	AB57_0489	phosphoenolpyruvate-protein phosphotransferase	-2.89	0.0011
AB57_RS15910	AB57_3225	head morphogenesis protein	-2.88	0.0019

Table S2. continued.

Locus tag	Locus tag	Function	Log_2 Fitness defect in spleen	*P* value
AB57_RS01135	AB57_0221	hypothetical protein	-2.86	0.0037
AB57_RS13225	AB57_2669	hypothetical protein	-2.85	0.0069
AB57_RS00580	AB57_0115	hypothetical protein	-2.82	0.0008
AB57_RS17200	AB57_3476	lytic transglycosylase	-2.81	0.0026
AB57_RS12470	AB57_2515	transglutaminase	-2.80	0.0014
AB57_RS15370	AB57_RS15370	pseudogene	-2.78	0.0037
AB57_RS01140	AB57_0222	hypothetical protein	-2.78	0.0042
AB57_RS14700	AB57_2970	hypothetical protein	-2.75	0.0003
AB57_RS06040	AB57_1195	MULTISPECIES: crotonase	-2.68	0.0017
AB57_RS12965	AB57_RS12965	pseudogene	-2.66	0.0036
AB57_RS09985	AB57_2006	pilus assembly protein	-2.65	0.0003
AB57_RS02075	AB57_0394	methylated-DNA--protein-cysteine methyltransferase	-2.65	0.0016
AB57_RS07780	AB57_1552	MULTISPECIES: GntR family transcriptional regulator	-2.65	0.0010
AB57_RS13685	AB57_2757	methionine aminopeptidase	-2.64	0.0041
AB57_RS04010	AB57_0782	5-methyltetrahydropteroyltriglutamate--homocysteine methyltransferase	-2.59	0.0035
AB57_RS18115	AB57_3659	peptide signal protein	-2.57	0.0045
AB57_RS18460	AB57_3724	quercetin 2,3-dioxygenase	-2.56	0.0009
AB57_RS02635	AB57_RS02635	pseudogene	-2.56	0.0000
AB57_RS01785	AB57_0341	hypothetical protein	-2.55	0.0006
AB57_RS01255	AB57_0247	protein TniQ	-2.54	0.0055
GenBank:repeat_region:NC_011586:3659118:3659357			-2.54	0.0029
AB57_RS01505	AB57_0289	MULTISPECIES: transposase	-2.52	0.0010
AB57_RS01780	AB57_0340	MULTISPECIES: phosphomethylpyrimidine synthase	-2.52	0.0018
AB57_RS16630	AB57_3368	hypothetical protein	-2.51	0.0061
AB57_RS02035	AB57_0386	3-ketoacyl-CoA thiolase	-2.51	0.0028
AB57_RS01030	AB57_0202	DNA-binding protein	-2.50	0.0020
AB57_RS15365		metalloprotease	-2.49	0.0039

Table S2. continued.

Locus tag	Locus tag	Function	Log $_2$ Fitness defect in spleen	*P* value
AB57_RS15980	AB57_3237	serine/threonine-protein phosphatase 1	-2.49	0.0029
AB57_RS01815	AB57_0347	argininosuccinate lyase	-2.48	0.0001
AB57_RS08515	AB57_1705	uridylyltransferase	-2.46	0.0030
GenBank:repeat_region:NC_011586:310258:311077			-2.45	0.0004
AB57_RS00915	AB57_0179	NAD-dependent malic enzyme	-2.43	0.0000
AB57_RS07775	AB57_1551	MULTISPECIES: hypothetical protein	-2.42	0.0014
AB57_RS03495	AB57_0684	short-chain dehydrogenase	-2.42	0.0030
AB57_RS04275	AB57_0835	MULTISPECIES: deoxycytidine triphosphate deaminase	-2.41	0.0001
AB57_RS08960	AB57_1797	biopolymer transporter ExbB	-2.37	0.0006
AB57_RS17030	AB57_3445	membrane protein	-2.37	0.0002
AB57_RS18140	AB57_3662	hypothetical protein	-2.34	0.0025
AB57_RS18545	AB57_3741	TetR family transcriptional regulator	-2.31	0.0001
AB57_RS00110	AB57_0022	ribonuclease P protein component	-2.31	0.0001
AB57_RS04440	AB57_0871	MULTISPECIES: hypothetical protein	-2.23	0.0077
AB57_RS13860	AB57_2789	MULTISPECIES: anthranilate phosphoribosyltransferase	-2.21	0.0003
AB57_RS01820	AB57_0348	alginate biosynthesis protein	-2.17	0.0028
AB57_RS18415	AB57_3715	hypothetical protein	-2.16	0.0036
AB57_RS12645	AB57_2551	DNA-binding response regulator	-2.14	0.0030
AB57_RS18830	AB57_3801	allantoinase	-2.13	0.0009
AB57_RS16495	AB57_3338	xanthine permease	-2.11	0.0049
AB57_RS08770	AB57_1759	hypothetical protein	-2.10	0.0052
AB57_RS15835	AB57_3210	hypothetical protein	-2.07	0.0008
AB57_RS11385	AB57_2288	hypothetical protein	-2.07	0.0008
AB57_RS07915	AB57_1580	MFS transporter	-1.99	0.0069
AB57_RS18255	AB57_3681	GNAT family acetyltransferase	-1.99	0.0009
AB57_RS10450	AB57_2093	GCN5 family N-acetyltransferase	-1.98	0.0030
AB57_RS16510	AB57_3342	RTCB protein	-1.97	0.0041
AB57_RS02240	AB57_0424	DNA helicase	-1.94	0.0003

Table S2. continued.

Locus tag	Locus tag	Function	Log_2 Fitness defect in spleen	*P* value
AB57_RS08765	AB57_1758	FxsA cytoplasmic membrane protein	-1.91	0.0003
AB57_RS01735	AB57_0331	iron transporter	-1.90	0.0053
AB57_RS17025	AB57_3444	GTPase HflX	-1.87	0.0001
AB57_RS18955	AB57_3826	TetR family transcriptional regulator	-1.87	0.0038
AB57_RS14270	AB57_2875	phosphonate ABC transporter substrate-binding protein	-1.85	0.0008
AB57_RS16240	AB57_3291	microcin B17 transporter	-1.85	0.0002
AB57_RS01285	AB57_0253	arsenate reductase	-1.81	0.0058
AB57_RS15975		MULTISPECIES: hypothetical protein	-1.79	0.0001
AB57_RS02010	AB57_0381	dithiol-disulfide isomerase	-1.78	0.0002
AB57_RS08025	AB57_1602	TetR family transcriptional regulator	-1.77	0.0028
AB57_RS16380	AB57_3314	MULTISPECIES: two-component system response regulator	-1.74	0.0012
AB57_RS00505	AB57_0099	hypothetical protein	-1.71	0.0070
AB57_RS09190	AB57_1844	MULTISPECIES: ribonuclease	-1.71	0.0077
AB57_RS00240	AB57_0049	protein GrpE	-1.66	0.0077
AB57_RS14605	AB57_2952	S-(hydroxymethyl)glutathione synthase	-1.65	0.0030
AB57_RS19480	AB57_RS19480	r01	-1.63	0.0003
AB57_RS19480		RNase_P_RNA	-1.63	0.0003
AB57_RS00145	AB57_0030	MULTISPECIES: transglycosylase	-1.63	0.0027
AB57_RS06420	AB57_1270	MULTISPECIES: terminase	-1.60	0.0069
AB57_RS07765	AB57_1549	catalase	-1.60	0.0030
AB57_RS08635	AB57_1731	transcriptional regulator	-1.55	0.0006
AB57_RS03310	AB57_0645	MULTISPECIES: hypothetical protein	-1.55	0.0069
AB57_RS18965	AB57_3828	histidine kinase	-1.48	0.0001
AB57_RS05780		hypothetical protein	-1.42	0.0014
AB57_RS15515	AB57_3142	putrescine/spermidine ABC transporter	-1.38	0.0042
AB57_RS15440	AB57_3126	2-oxoglutarate dehydrogenase subunit E1	-1.37	0.0008
AB57_RS18050	AB57_3646	hypothetical protein	-1.37	0.0008
AB57_RS01290	AB57_0254	MULTISPECIES: arsenic resistance protein	-1.36	0.0077
AB57_RS09455	AB57_1897	hypothetical protein	-1.33	0.0030
AB57_RS02090	AB57_0397	fusaric acid resistance protein	-1.30	0.0004

Table S2. continued.

Locus tag	Locus tag	Function	Log_2 Fitness defect in spleen	*P* value
AB57_RS12535	AB57_2529	methionine ABC transporter permease	-1.30	0.0024
AB57_RS12310	AB57_2481	MULTISPECIES: butyryl-CoA dehydrogenase	-1.28	0.0030
AB57_RS02770		TetR family transcriptional regulator	-1.27	0.0006
AB57_RS02300	AB57_0437	EstA family serine hydrolase	-1.27	0.0008
AB57_RS13355	AB57_2692	hypothetical protein	-1.26	0.0016
AB57_RS03605	AB57_0706	MULTISPECIES: integration host factor subunit alpha	-1.24	0.0062
AB57_RS08760	AB57_1757	carbapenem-hydrolyzing class D beta-lactamase OXA-69	-1.23	0.0069
AB57_RS07690	AB57_1532	endonuclease	-1.21	0.0012
AB57_RS18370	AB57_3706	NAD(P)H oxidoreductase	-1.16	0.0030
AB57_RS18495	AB57_3731	DNA-binding protein	-1.11	0.0002
AB57_RS02435	AB57_0465	MULTISPECIES: aldehyde-activating protein	-1.10	0.0055
AB57_RS14530	AB57_2937	hypothetical protein	-1.10	0.0001
AB57_RS01625	AB57_0309	MULTISPECIES: nitrogen regulatory protein P-II 1	-1.06	0.0003
AB57_RS17290	AB57_4819	tRNA-Arg	-1.05	0.0030
AB57_RS17295	AB57_4820	tRNA-Arg	-1.05	0.0030
AB57_RS18765	AB57_3788	glutathione synthetase	-1.03	0.0068
AB57_RS06790	AB57_1346	benzoate transporter	-1.02	0.0091
AB57_RS06825	AB57_1353	ATPase	-1.02	0.0030

Table S3. *A. baumannii* lytic transglycosylases and their closest homolog.

Gene	Predicted function	Pfam[a]	Fitness defect[b]	Homolog (species)	% identity / positive	Homolog with AB5075[c]
AB57_0044	Lytic transglycosylase	SLT_2; PG_binding_1; PG_binding_3; SLT	-355	*PA1171* (*Pseudomonas aeruginosa)*	60 / 75	*ABUW_3883*
AB57_1136	Lytic transglycosylase	SLT; SLT_2	-21	*mltD* (*Pseudomonas aeruginosa)*	48 / 67	*ABUW_2840*
AB57_2749 (*mltB*)	Lytic transglycosylase B	SLT_2	-655	*mltB* (*Escherichia coli*)	41 / 56	*ABUW_1243* (*mltB*)
AB57_3476	Lytic transglycosylase	SLT; SLT_L	-7	*mltE* (*Neisseria gonorrhoeae*)	42 / 58	*ABUW_0465*

[a] **Pfam**

SLT_2: Transglycosylase SLT domain.
PG_binding_1: Putative peptidoglycan binding domain.
PG_binding_3: Predicted Peptidoglycan domain.
SLT: Transglycosylase SLT domain.
SLT_L: Soluble lytic transglycosylase L domain.

[b] Tn-seq calculated fitness defect in the spleen at 24 hpi.

[c] All genes share 100% identity with strain AB5075.

Table S4. ***A. baumannii*** **peptidoglycan composition.**

Peak	Name	Relative % of each Muropeptide					
		LB			50% HI serum[b]		
		57	57 Δ*mltB*	57 Δ*mltB* compl.	57	57 Δ*mltB*	57 Δ*mltB* compl.
1	**Tri**	0.96 ± 0.05	2.11 ± 0.09	1.60 ± 0.27	2.22 ± 0.26	3.54 ± 0.03	2.36 ± 0.16
2	**TetraGly4**	1.41 ± 0.01	1.25 ± 0.36	1.52 ± 0.27	0.64 ± 0.41	0.52 ± 0.23	0.48 ± 0.16
3	**Tetra**	22.82 ± 3.18	20.12 ± 0.11	24.90 ± 3.26	21.06 ± 0.30	21.94 ± 1.19	22.91 ± 0.01
4	**TetraTri**	4.71 ± 0.04	4.78 ± 0.21	4.23 ± 0.24	4.33 ± 0.54	4.04 ± 0.58	3.03 ± 0.07
5	**TetraTetra**	41.51 ± 0.80	41.82 ± 1.58	44.38 ± 0.04	35.16 ± 0.57	33.57 ± 0.62	37.32 ± 0.38
6	**TetraTetraTri**	1.01 ± 0.31	1.16 ± 0.09	0.71 ± 0.40	0.81 ± 0.12	0.43 ± 0.20	0.19 ± 0.39
7	**TetraTetraTetra**	17.3 ± 1.60	17.60 ± 1.10	15.44 ± 2.47	18.80 ± 0.21	16.11 ± 0.55	18.53 ± 0.99
8	**TetraTetraTetraTri (~30%) / TetraTriAnh (~70%)**	3.38 ± 1.28	4.17 ± 0.11	2.00 ± 1.11	5.46 ± 0.35	4.28 ± 0.41	4.64 ± 0.20
9	**TetraTetraAnh I**	0.46 ± 0.92	0.56 ± 0.03	0.00 ± 0.00	1.45 ± 0.17	1.26 ± 0.03	0.98 ± 0.39
10	**TetraTetraAnh II**	1.57 ± 0.29	1.10 ± 0.02	1.19 ± 0.02	1.57 ± 0.33	1.50 ± 0.07	1.55 ± 0.46
11	**TetraTetraTetraAnh**	0.99 ± 0.04	0.83 ± 0.00	0.73 ± 0.17	1.46 ± 0.22	1.17 ± 0.06	1.35 ± 0.53
**Sum of known peaks**		96.12 ± 1.19	94.72 ± 2.94	96.71 ± 0.52	92.95 ± 0.93	88.35 ± 1.15	93.35 ± 2.02
**Monomers**		26.23 ± 3.56	24.80 ± 0.35	28.97 ± 4.10	25.73 ± 0.78	29.42 ± 0.67	27.59 ± 0.26
**Dimers**		52.65 ± 1.5	53.45 ± 0.08	52.95 ± 0.72	49.85 ± 0.10	49.08 ± 1.02	49.42 ± 0.52
**Trimers**		20.07 ± 1.67	20.68 ± 0.43	17.46 ± 3.05	22.66 ± 0.56	20.05 ± 0.52	21.51 ± 0.75
**Tetramers**		1.05 ± 0.39	1.07 ± 0.00	0.62 ± 0.34	1.76 ± 0.13	1.45 ± 0.16	1.49 ± 0.03
**% peptides in cross linkage**		73.77 ± 3.56	75.20 ± 0.35	71.03 ± 4.10	74.27 ± 0.79	70.58 ± 0.67	72.41 ± 0.26
**Average chain length**		38.6 ± 8.57	41.29 ± 0.21	63.93 ± 17.69	23.88 ± 2.95	27.06 ± 2.10	27.98 ± 1.70

[a]Percentages calculated as per (Glauner *et al.*) and are mean ± variation of two independent experiments.

[b] HI : Heat-inactivated.

Table S5. Comparative analysis of candidate fitness factors with other Tn-seq analyses.

With ATCC17978 (pneumonia infection model)[a]					
AB0057	ATCC17978	AB0057	ATCC17978	AB0057	ATCC17978
Locus_tag	Locus_tag	Locus_tag	Locus_tag	Locus_tag	Locus_tag
AB57_RS12945	AUO97_00155	AB57_RS18310	AUO97_05240	AB57_RS03160	AUO97_10075
AB57_RS12950	AUO97_00160	AB57_RS18780	AUO97_05760	AB57_RS03205	AUO97_10120
AB57_RS12970	AUO97_00180	AB57_RS18815	AUO97_05795	AB57_RS03225	AUO97_10145
AB57_RS13635	AUO97_00500	AB57_RS00945	AUO97_06465	AB57_RS03230	AUO97_10150
AB57_RS13645	AUO97_00510	AB57_RS00930	AUO97_06480	AB57_RS03510	AUO97_10340
AB57_RS13685	AUO97_00550	AB57_RS00580	AUO97_06870	AB57_RS03605	AUO97_10455
AB57_RS13845	AUO97_00695	AB57_RS00545	AUO97_06875	AB57_RS03640	AUO97_10490
AB57_RS13860	AUO97_00710	AB57_RS00575	AUO97_06875	AB57_RS03705	AUO97_10555
AB57_RS13865	AUO97_00715	AB57_RS00570	AUO97_06880	AB57_RS03765	AUO97_10615
AB57_RS14000	AUO97_00845	AB57_RS00380	AUO97_07045	AB57_RS03850	AUO97_10700
AB57_RS14545	AUO97_01375	AB57_RS00210	AUO97_07215	AB57_RS04005	AUO97_10860
AB57_RS14550	AUO97_01380	AB57_RS00205	AUO97_07220	AB57_RS04010	AUO97_10865
AB57_RS14735	AUO97_01580	AB57_RS19355	AUO97_07450	AB57_RS04080	AUO97_10935
AB57_RS14805	AUO97_01835	AB57_RS19330	AUO97_07475	AB57_RS04085	AUO97_10940
AB57_RS14910	AUO97_01940	AB57_RS19195	AUO97_07610	AB57_RS04245	AUO97_11095
AB57_RS15340	AUO97_02405	AB57_RS19190	AUO97_07615	AB57_RS04255	AUO97_11105
AB57_RS16130	AUO97_02820	AB57_RS19005	AUO97_07800	AB57_RS04275	AUO97_11130
AB57_RS16180	AUO97_02865	AB57_RS01710	AUO97_08630	AB57_RS05460	AUO97_12215
AB57_RS16325	AUO97_03015	AB57_RS01715	AUO97_08635	AB57_RS05465	AUO97_12220
AB57_RS16680	AUO97_03355	AB57_RS01720	AUO97_08640	AB57_RS05605	AUO97_12370
AB57_RS16685	AUO97_03360	AB57_RS01805	AUO97_08725	AB57_RS05720	AUO97_12500
AB57_RS16810	AUO97_03485	AB57_RS01815	AUO97_08735	AB57_RS06700	AUO97_13355
AB57_RS16830	AUO97_03505	AB57_RS01885	AUO97_08805	AB57_RS08485	AUO97_14850
AB57_RS16900	AUO97_03620	AB57_RS02050	AUO97_08975	AB57_RS08785	AUO97_15155
AB57_RS17190	AUO97_04105	AB57_RS02245	AUO97_09165	AB57_RS08930	AUO97_15295
AB57_RS17560	AUO97_04475	AB57_RS02565	AUO97_09495	AB57_RS09030	AUO97_15395
AB57_RS17590	AUO97_04505	AB57_RS02580	AUO97_09510	AB57_RS09505	AUO97_16165
AB57_RS17595	AUO97_04510	AB57_RS02585	AUO97_09515	AB57_RS11570	AUO97_17785
AB57_RS17605	AUO97_04520	AB57_RS02590	AUO97_09520	AB57_RS12185	AUO97_18735
AB57_RS17750	AUO97_04660	AB57_RS02595	AUO97_09525	AB57_RS12515	AUO97_19060
AB57_RS17850	AUO97_04760	AB57_RS02620	AUO97_09550	AB57_RS12520	AUO97_19065
AB57_RS17855	AUO97_04765	AB57_RS02705	AUO97_09620		
AB57_RS17960	AUO97_04870	AB57_RS02965	AUO97_09785		
AB57_RS18015	AUO97_04925	AB57_RS02970	AUO97_09790		
AB57_RS18020	AUO97_04930	AB57_RS02975	AUO97_09795		
AB57_RS18200	AUO97_05110	AB57_RS03060	AUO97_09880		
AB57_RS18260	AUO97_05185	AB57_RS03100	AUO97_09920		
AB57_RS18265	AUO97_05190	AB57_RS03110	AUO97_09930		
AB57_RS18280	AUO97_05205	AB57_RS03155	AUO97_10070		

Table S5. continued.

With ATCC17978 (bloodstream infection model)[b]			
AB0057	ATCC17978	AB0057	ATCC17978
Locus_tag	Locus_tag	Locus_tag	Locus_tag
AB57_RS13635	AUO97_00500	AB57_RS05825	AUO97_12645
AB57_RS14210	AUO97_01065	AB57_RS06100	AUO97_12885
AB57_RS14805	AUO97_01835	AB57_RS07165	AUO97_13700
AB57_RS15395	AUO97_02465	AB57_RS11515	AUO97_17730
AB57_RS15660	AUO97_02735	AB57_RS11885	AUO97_18455
AB57_RS16130	AUO97_02820	AB57_RS12140	AUO97_18690
AB57_RS16365	AUO97_03045	AB57_RS12530	AUO97_19075
AB57_RS16710	AUO97_03385		
AB57_RS16775	AUO97_03450		
AB57_RS17315	AUO97_04230		
AB57_RS17325	AUO97_04240		
AB57_RS18030	AUO97_04940		
AB57_RS18435	AUO97_05405		
AB57_RS18810	AUO97_05790		
AB57_RS00865	AUO97_06545		
AB57_RS00835	AUO97_06575		
AB57_RS00310	AUO97_07115		
AB57_RS00250	AUO97_07175		
AB57_RS19355	AUO97_07450		
AB57_RS18935	AUO97_07885		
AB57_RS00640	AUO97_08400		
AB57_RS01670	AUO97_08590		
AB57_RS01690	AUO97_08610		
AB57_RS01865	AUO97_08785		
AB57_RS01870	AUO97_08790		
AB57_RS02000	AUO97_08920		
AB57_RS02590	AUO97_09520		
AB57_RS02765	AUO97_09680		
AB57_RS02945	AUO97_09765		
AB57_RS03025	AUO97_09845		
AB57_RS04295	AUO97_11150		
AB57_RS04305	AUO97_11160		
AB57_RS04465	AUO97_11320		
AB57_RS04475	AUO97_11330		
AB57_RS04845	AUO97_11700		
AB57_RS05125	AUO97_11875		
AB57_RS05185	AUO97_11935		
AB57_RS05490	AUO97_12250		
AB57_RS05495	AUO97_12255		

Table S5. continued.

With AB5075 (*Galleria mellonella* infection model)[c]					
**AB0057**	**AB5075**	**AB0057**	**AB5075**	**AB0057**	**AB5075**
**Locus_tag**	**Locus_tag**	**Locus_tag**	**Locus_tag**	**Locus_tag**	**Locus_tag**
AB57_RS00095	ABUW_0002	AB57_RS17225	ABUW_0460	AB57_RS15870	ABUW_0786
AB57_RS00005	ABUW_0014	AB57_RS17195	ABUW_0466	AB57_RS15835	ABUW_0792
AB57_RS19385	ABUW_0020	AB57_RS17165	ABUW_0472	AB57_RS15820	ABUW_0796
AB57_RS19200	ABUW_0058	AB57_RS17100	ABUW_0486	AB57_RS15815	ABUW_0797
AB57_RS19190	ABUW_0060	AB57_RS17070	ABUW_0492	AB57_RS15810	ABUW_0798
AB57_RS19185	ABUW_0061	AB57_RS17050	ABUW_0496	AB57_RS15735	ABUW_0814
AB57_RS19035	ABUW_0091	AB57_RS17045	ABUW_0497	AB57_RS15730	ABUW_0815
AB57_RS18915	ABUW_0115	AB57_RS17030	ABUW_0500	AB57_RS15670	ABUW_0827
AB57_RS18890	ABUW_0120	AB57_RS16960	ABUW_0514	AB57_RS15660	ABUW_0829
AB57_RS18885	ABUW_0121	AB57_RS16915	ABUW_0523	AB57_RS15600	ABUW_0841
AB57_RS18870	ABUW_0124	AB57_RS16890	ABUW_0528	AB57_RS15595	ABUW_0842
AB57_RS18815	ABUW_0135	AB57_RS16885	ABUW_0529	AB57_RS15590	ABUW_0843
AB57_RS18715	ABUW_0155	AB57_RS16840	ABUW_0585	AB57_RS15585	ABUW_0844
AB57_RS18710	ABUW_0156	AB57_RS16810	ABUW_0591	AB57_RS15540	ABUW_0852
AB57_RS18675	ABUW_0163	AB57_RS16805	ABUW_0592	AB57_RS15535	ABUW_0853
AB57_RS18625	ABUW_0174	AB57_RS16800	ABUW_0593	AB57_RS15485	ABUW_0863
AB57_RS18370	ABUW_0228	AB57_RS16795	ABUW_0594	AB57_RS15470	ABUW_0866
AB57_RS18365	ABUW_0229	AB57_RS16775	ABUW_0598	AB57_RS15415	ABUW_0877
AB57_RS18330	ABUW_0236	AB57_RS16670	ABUW_0619	AB57_RS15400	ABUW_0882
AB57_RS18200	ABUW_0262	AB57_RS16635	ABUW_0626	AB57_RS15365	ABUW_0888
AB57_RS18190	ABUW_0264	AB57_RS16615	ABUW_0630	AB57_RS15360	ABUW_0889
AB57_RS18140	ABUW_0274	AB57_RS16595	ABUW_0634	AB57_RS15340	ABUW_0893
AB57_RS18120	ABUW_0278	AB57_RS16550	ABUW_0643	AB57_RS15305	ABUW_0900
AB57_RS18065	ABUW_0289	AB57_RS16545	ABUW_0644	AB57_RS15295	ABUW_0902
AB57_RS17985	ABUW_0305	AB57_RS16540	ABUW_0645	AB57_RS15285	ABUW_0904
AB57_RS17955	ABUW_0311	AB57_RS16480	ABUW_0657	AB57_RS15265	ABUW_0908
AB57_RS17870	ABUW_0328	AB57_RS16460	ABUW_0660	AB57_RS15250	ABUW_0911
AB57_RS17855	ABUW_0331	AB57_RS16390	ABUW_0677	AB57_RS15245	ABUW_0912
AB57_RS17700	ABUW_0364	AB57_RS16355	ABUW_0684	AB57_RS15235	ABUW_0914
AB57_RS17640	ABUW_0376	AB57_RS16350	ABUW_0685	AB57_RS15230	ABUW_0915
AB57_RS17625	ABUW_0379	AB57_RS16325	ABUW_0690	AB57_RS15195	ABUW_0922
AB57_RS17620	ABUW_0380	AB57_RS16285	ABUW_0698	AB57_RS15190	ABUW_0923
AB57_RS17595	ABUW_0385	AB57_RS16240	ABUW_0707	AB57_RS15145	ABUW_0932
AB57_RS17560	ABUW_0392	AB57_RS16190	ABUW_0717	AB57_RS15140	ABUW_0933
AB57_RS17545	ABUW_0395	AB57_RS16180	ABUW_0719	AB57_RS15135	ABUW_0934
AB57_RS17525	ABUW_0399	AB57_RS16170	ABUW_0721	AB57_RS15070	ABUW_0948
AB57_RS17310	ABUW_0443	AB57_RS16135	ABUW_0728	AB57_RS14980	ABUW_0966
AB57_RS17305	ABUW_0444	AB57_RS16115	ABUW_0732	AB57_RS14975	ABUW_0967
AB57_RS17285	ABUW_0448	AB57_RS15975	ABUW_0763	AB57_RS14970	ABUW_0968
AB57_RS17250	ABUW_0455	AB57_RS15940	ABUW_0771	AB57_RS14950	ABUW_0973

Table S5. continued.

With AB5075 (*Galleria mellonella* infection model)[c]					
**AB0057**	**AB5075**	**AB0057**	**AB5075**	**AB0057**	**AB5075**
**Locus_tag**	**Locus_tag**	**Locus_tag**	**Locus_tag**	**Locus_tag**	**Locus_tag**
AB57_RS14905	ABUW_0982	AB57_RS14260	ABUW_1117	AB57_RS13320	ABUW_1311
AB57_RS14875	ABUW_0988	AB57_RS14245	ABUW_1120	AB57_RS13295	ABUW_1316
AB57_RS14860	ABUW_0991	AB57_RS14230	ABUW_1123	AB57_RS15765	ABUW_1323
AB57_RS14825	ABUW_0998	AB57_RS14215	ABUW_1126	AB57_RS13225	ABUW_1330
AB57_RS14820	ABUW_0999	AB57_RS14210	ABUW_1127	AB57_RS13215	ABUW_1332
AB57_RS14815	ABUW_1000	AB57_RS14205	ABUW_1128	AB57_RS13125	ABUW_1351
AB57_RS14800	ABUW_1003	AB57_RS14180	ABUW_1133	AB57_RS13115	ABUW_1353
AB57_RS14790	ABUW_1005	AB57_RS14175	ABUW_1134	AB57_RS13110	ABUW_1354
AB57_RS14785	ABUW_1006	AB57_RS14165	ABUW_1136	AB57_RS13100	ABUW_1356
AB57_RS14760	ABUW_1011	AB57_RS14150	ABUW_1139	AB57_RS13070	ABUW_1362
AB57_RS14735	ABUW_1016	AB57_RS14135	ABUW_1142	AB57_RS13065	ABUW_1363
AB57_RS14710	ABUW_1021	AB57_RS14130	ABUW_1143	AB57_RS13010	ABUW_1374
AB57_RS14705	ABUW_1022	AB57_RS14095	ABUW_1150	AB57_RS16015	ABUW_1409
AB57_RS14680	ABUW_1027	AB57_RS14045	ABUW_1161	AB57_RS12960	ABUW_1443
AB57_RS14670	ABUW_1029	AB57_RS14040	ABUW_1162	AB57_RS12950	ABUW_1445
AB57_RS14655	ABUW_1032	AB57_RS14015	ABUW_1168	AB57_RS12865	ABUW_1462
AB57_RS14640	ABUW_1035	AB57_RS14005	ABUW_1170	AB57_RS12785	ABUW_1478
AB57_RS14615	ABUW_1040	AB57_RS14000	ABUW_1173	AB57_RS12775	ABUW_1480
AB57_RS14605	ABUW_1042	AB57_RS13970	ABUW_1179	AB57_RS12645	ABUW_1506
AB57_RS14585	ABUW_1046	AB57_RS13875	ABUW_1198	AB57_RS12560	ABUW_1523
AB57_RS14580	ABUW_1047	AB57_RS13860	ABUW_1201	AB57_RS12545	ABUW_1526
AB57_RS14550	ABUW_1053	AB57_RS13850	ABUW_1203	AB57_RS12540	ABUW_1527
AB57_RS14540	ABUW_1055	AB57_RS13845	ABUW_1204	AB57_RS12515	ABUW_1532
AB57_RS14530	ABUW_1057	AB57_RS13805	ABUW_1212	AB57_RS12505	ABUW_1534
AB57_RS14515	ABUW_1060	AB57_RS13780	ABUW_1217	AB57_RS12500	ABUW_1535
AB57_RS14510	ABUW_1061	AB57_RS13765	ABUW_1220	AB57_RS12490	ABUW_1537
AB57_RS14505	ABUW_1062	AB57_RS13715	ABUW_1228	AB57_RS12485	ABUW_1539
AB57_RS14500	ABUW_1063	AB57_RS13695	ABUW_1232	AB57_RS12470	ABUW_1542
AB57_RS14455	ABUW_1072	AB57_RS13645	ABUW_1242	AB57_RS12465	ABUW_1543
AB57_RS14450	ABUW_1073	AB57_RS13640	ABUW_1243	AB57_RS12460	ABUW_1544
AB57_RS14400	ABUW_1085	AB57_RS13635	ABUW_1244	AB57_RS12355	ABUW_1564
AB57_RS14385	ABUW_1088	AB57_RS13620	ABUW_1247	AB57_RS12350	ABUW_1565
AB57_RS14380	ABUW_1089	AB57_RS13510	ABUW_1270	AB57_RS12340	ABUW_1567
AB57_RS14355	ABUW_1098	AB57_RS16025	ABUW_1271	AB57_RS12310	ABUW_1573
AB57_RS14335	ABUW_1102	AB57_RS13480	ABUW_1278	AB57_RS12295	ABUW_1576
AB57_RS14305	ABUW_1108	AB57_RS13435	ABUW_1288	AB57_RS12195	ABUW_1597
AB57_RS14290	ABUW_1111	AB57_RS13415	ABUW_1292	AB57_RS12190	ABUW_1598
AB57_RS14285	ABUW_1112	AB57_RS13405	ABUW_1294	AB57_RS12180	ABUW_1600
AB57_RS14280	ABUW_1113	AB57_RS15910	ABUW_1294	AB57_RS12170	ABUW_1602
AB57_RS14265	ABUW_1116	AB57_RS13355	ABUW_1304	AB57_RS12165	ABUW_1603

Table S5. continued.

With AB5075 (*Galleria mellonella* infection model)[c]					
**AB0057**	**AB5075**	**AB0057**	**AB5075**	**AB0057**	**AB5075**
**Locus_tag**	**Locus_tag**	**Locus_tag**	**Locus_tag**	**Locus_tag**	**Locus_tag**
AB57_RS12155	ABUW_1605	AB57_RS11230	ABUW_1802	AB57_RS09190	ABUW_2214
AB57_RS12145	ABUW_1607	AB57_RS11225	ABUW_1803	AB57_RS09135	ABUW_2225
AB57_RS12140	ABUW_1608	AB57_RS11220	ABUW_1804	AB57_RS09095	ABUW_2234
AB57_RS12065	ABUW_1625	AB57_RS11210	ABUW_1806	AB57_RS09090	ABUW_2235
AB57_RS12055	ABUW_1627	AB57_RS11195	ABUW_1809	AB57_RS09085	ABUW_2236
AB57_RS12040	ABUW_1630	AB57_RS11190	ABUW_1811	AB57_RS09000	ABUW_2253
AB57_RS11900	ABUW_1661	AB57_RS11185	ABUW_1812	AB57_RS08995	ABUW_2254
AB57_RS11885	ABUW_1664	AB57_RS11170	ABUW_1815	AB57_RS08965	ABUW_2260
AB57_RS11855	ABUW_1671	AB57_RS11025	ABUW_1844	AB57_RS08960	ABUW_2261
AB57_RS11850	ABUW_1672	AB57_RS11000	ABUW_1849	AB57_RS08955	ABUW_2262
AB57_RS11845	ABUW_1673	AB57_RS10875	ABUW_1874	AB57_RS08930	ABUW_2267
AB57_RS11790	ABUW_1685	AB57_RS10700	ABUW_1910	AB57_RS08925	ABUW_2268
AB57_RS11785	ABUW_1686	AB57_RS10640	ABUW_1922	AB57_RS08895	ABUW_2274
AB57_RS11775	ABUW_1688	AB57_RS10625	ABUW_1925	AB57_RS08875	ABUW_2278
AB57_RS11740	ABUW_1695	AB57_RS10570	ABUW_1936	AB57_RS08850	ABUW_2282
AB57_RS11735	ABUW_1696	AB57_RS10545	ABUW_1943	AB57_RS08835	ABUW_2285
AB57_RS11690	ABUW_1706	AB57_RS10450	ABUW_1964	AB57_RS08785	ABUW_2295
AB57_RS11650	ABUW_1714	AB57_RS10395	ABUW_1975	AB57_RS08770	ABUW_2298
AB57_RS11610	ABUW_1722	AB57_RS09985	ABUW_2053	AB57_RS08765	ABUW_2299
AB57_RS11575	ABUW_1729	AB57_RS09980	ABUW_2054	AB57_RS08760	ABUW_2300
AB57_RS11560	ABUW_1732	AB57_RS09970	ABUW_2056	AB57_RS08755	ABUW_2301
AB57_RS11555	ABUW_1733	AB57_RS09965	ABUW_2057	AB57_RS08750	ABUW_2302
AB57_RS11550	ABUW_1734	AB57_RS09905	ABUW_2069	AB57_RS08740	ABUW_2305
AB57_RS11525	ABUW_1739	AB57_RS09895	ABUW_2072	AB57_RS08725	ABUW_2308
AB57_RS11520	ABUW_1740	AB57_RS09665	ABUW_2118	AB57_RS08710	ABUW_2311
AB57_RS11515	ABUW_1741	AB57_RS09630	ABUW_2125	AB57_RS08620	ABUW_2330
AB57_RS11510	ABUW_1742	AB57_RS09625	ABUW_2126	AB57_RS08545	ABUW_2345
AB57_RS11490	ABUW_1746	AB57_RS09590	ABUW_2133	AB57_RS08535	ABUW_2347
AB57_RS11480	ABUW_1748	AB57_RS09585	ABUW_2134	AB57_RS08520	ABUW_2350
AB57_RS11475	ABUW_1749	AB57_RS09555	ABUW_2140	AB57_RS08510	ABUW_2352
AB57_RS11470	ABUW_1750	AB57_RS09510	ABUW_2149	AB57_RS08505	ABUW_2353
AB57_RS11395	ABUW_1767	AB57_RS09505	ABUW_2150	AB57_RS08495	ABUW_2355
AB57_RS11380	ABUW_1770	AB57_RS09455	ABUW_2161	AB57_RS08480	ABUW_2358
AB57_RS11375	ABUW_1771	AB57_RS09445	ABUW_2163	AB57_RS08475	ABUW_2359
AB57_RS11370	ABUW_1772	AB57_RS09425	ABUW_2167	AB57_RS08465	ABUW_2361
AB57_RS11350	ABUW_1776	AB57_RS09410	ABUW_2170	AB57_RS08460	ABUW_2362
AB57_RS11345	ABUW_1777	AB57_RS09370	ABUW_2178	AB57_RS08455	ABUW_2363
AB57_RS11335	ABUW_1779	AB57_RS09350	ABUW_2182	AB57_RS08435	ABUW_2367
AB57_RS11330	ABUW_1780	AB57_RS09220	ABUW_2208	AB57_RS08335	ABUW_2387
AB57_RS11240	ABUW_1799	AB57_RS09215	ABUW_2209	AB57_RS08330	ABUW_2389

Table S5. continued.

With AB5075 (*Galleria mellonella* infection model)[c]					
**AB0057**	**AB5075**	**AB0057**	**AB5075**	**AB0057**	**AB5075**
**Locus_tag**	**Locus_tag**	**Locus_tag**	**Locus_tag**	**Locus_tag**	**Locus_tag**
AB57_RS08325	ABUW_2390	AB57_RS07090	ABUW_2652	AB57_RS05780	ABUW_2829
AB57_RS08230	ABUW_2409	AB57_RS07030	ABUW_2664	AB57_RS05760	ABUW_2832
AB57_RS08135	ABUW_2428	AB57_RS07020	ABUW_2666	AB57_RS05730	ABUW_2838
AB57_RS08130	ABUW_2429	AB57_RS07015	ABUW_2667	AB57_RS05725	ABUW_2839
AB57_RS08125	ABUW_2430	AB57_RS07005	ABUW_2669	AB57_RS05720	ABUW_2840
AB57_RS08005	ABUW_2455	AB57_RS06935	ABUW_2683	AB57_RS05710	ABUW_2842
AB57_RS07990	ABUW_2458	AB57_RS06905	ABUW_2690	AB57_RS05705	ABUW_2843
AB57_RS07915	ABUW_2473	AB57_RS06815	ABUW_2709	AB57_RS05690	ABUW_2846
AB57_RS07910	ABUW_2474	AB57_RS06790	ABUW_2714	AB57_RS05675	ABUW_2849
AB57_RS07905	ABUW_2475	AB57_RS06775	ABUW_2717	AB57_RS05670	ABUW_2850
AB57_RS07805	ABUW_2496	AB57_RS06770	ABUW_2718	AB57_RS05665	ABUW_2851
AB57_RS07775	ABUW_2502	AB57_RS06760	ABUW_2720	AB57_RS05660	ABUW_2852
AB57_RS07770	ABUW_2503	AB57_RS06755	ABUW_2721	AB57_RS05630	ABUW_2858
AB57_RS07765	ABUW_2504	AB57_RS06750	ABUW_2722	AB57_RS05615	ABUW_2862
AB57_RS07695	ABUW_2521	AB57_RS06690	ABUW_2734	AB57_RS05585	ABUW_2869
AB57_RS07690	ABUW_2522	AB57_RS06650	ABUW_2742	AB57_RS05580	ABUW_2870
AB57_RS07620	ABUW_2536	AB57_RS06170	ABUW_2752	AB57_RS05575	ABUW_2871
AB57_RS07550	ABUW_2555	AB57_RS06140	ABUW_2758	AB57_RS05565	ABUW_2874
AB57_RS07505	ABUW_2564	AB57_RS06105	ABUW_2765	AB57_RS05560	ABUW_2875
AB57_RS07490	ABUW_2567	AB57_RS06100	ABUW_2766	AB57_RS05555	ABUW_2876
AB57_RS07485	ABUW_2568	AB57_RS06095	ABUW_2767	AB57_RS05550	ABUW_2877
AB57_RS07480	ABUW_2569	AB57_RS06055	ABUW_2775	AB57_RS05525	ABUW_2882
AB57_RS07460	ABUW_2573	AB57_RS06035	ABUW_2779	AB57_RS05485	ABUW_2890
AB57_RS07435	ABUW_2578	AB57_RS06030	ABUW_2780	AB57_RS05480	ABUW_2892
AB57_RS07430	ABUW_2579	AB57_RS06015	ABUW_2783	AB57_RS05465	ABUW_2895
AB57_RS07340	ABUW_2598	AB57_RS06010	ABUW_2784	AB57_RS05460	ABUW_2896
AB57_RS07335	ABUW_2599	AB57_RS06005	ABUW_2785	AB57_RS05455	ABUW_2898
AB57_RS07320	ABUW_2602	AB57_RS05985	ABUW_2789	AB57_RS05445	ABUW_2900
AB57_RS07315	ABUW_2603	AB57_RS05960	ABUW_2794	AB57_RS05425	ABUW_2904
AB57_RS07280	ABUW_2611	AB57_RS05940	ABUW_2798	AB57_RS05410	ABUW_2907
AB57_RS07255	ABUW_2617	AB57_RS17765	ABUW_2800	AB57_RS05400	ABUW_2909
AB57_RS07250	ABUW_2618	AB57_RS05915	ABUW_2803	AB57_RS05370	ABUW_2915
AB57_RS07220	ABUW_2626	AB57_RS05855	ABUW_2815	AB57_RS05350	ABUW_2920
AB57_RS07175	ABUW_2635	AB57_RS05850	ABUW_2816	AB57_RS05335	ABUW_2923
AB57_RS07165	ABUW_2637	AB57_RS05845	ABUW_2817	AB57_RS05330	ABUW_2924
AB57_RS07160	ABUW_2638	AB57_RS05825	ABUW_2820	AB57_RS05275	ABUW_2935
AB57_RS07155	ABUW_2639	AB57_RS05805	ABUW_2824	AB57_RS05265	ABUW_2937
AB57_RS07145	ABUW_2641	AB57_RS05800	ABUW_2825	AB57_RS05260	ABUW_2939
AB57_RS07135	ABUW_2643	AB57_RS05795	ABUW_2826	AB57_RS05240	ABUW_2943
AB57_RS07130	ABUW_2644	AB57_RS05785	ABUW_2828	AB57_RS05175	ABUW_2956

Table S5. continued.

With AB5075 (*Galleria mellonella* infection model)[c]					
**AB0057**	**AB5075**	**AB0057**	**AB5075**	**AB0057**	**AB5075**
**Locus_tag**	**Locus_tag**	**Locus_tag**	**Locus_tag**	**Locus_tag**	**Locus_tag**
AB57_RS05155	ABUW_2960	AB57_RS04005	ABUW_3198	AB57_RS02370	ABUW_3506
AB57_RS05145	ABUW_2962	AB57_RS03980	ABUW_3204	AB57_RS02345	ABUW_3511
AB57_RS05135	ABUW_2964	AB57_RS03975	ABUW_3205	AB57_RS02335	ABUW_3513
AB57_RS05085	ABUW_2974	AB57_RS03955	ABUW_3209	AB57_RS02325	ABUW_3515
AB57_RS05080	ABUW_2975	AB57_RS03830	ABUW_3234	AB57_RS02290	ABUW_3522
AB57_RS05075	ABUW_2976	AB57_RS03820	ABUW_3236	AB57_RS02285	ABUW_3523
AB57_RS04905	ABUW_3016	AB57_RS03800	ABUW_3240	AB57_RS02210	ABUW_3538
AB57_RS04900	ABUW_3017	AB57_RS03790	ABUW_3242	AB57_RS02085	ABUW_3563
AB57_RS04865	ABUW_3024	AB57_RS03775	ABUW_3245	AB57_RS01955	ABUW_3589
AB57_RS04840	ABUW_3029	AB57_RS03750	ABUW_3250	AB57_RS01850	ABUW_3610
AB57_RS04830	ABUW_3031	AB57_RS03705	ABUW_3259	AB57_RS01830	ABUW_3614
AB57_RS04780	ABUW_3041	AB57_RS03695	ABUW_3261	AB57_RS01720	ABUW_3636
AB57_RS04765	ABUW_3045	AB57_RS03655	ABUW_3269	AB57_RS01710	ABUW_3638
AB57_RS04755	ABUW_3047	AB57_RS03615	ABUW_3277	AB57_RS01660	ABUW_3648
AB57_RS04730	ABUW_3052	AB57_RS03590	ABUW_3282	AB57_RS01650	ABUW_3650
AB57_RS04715	ABUW_3055	AB57_RS03585	ABUW_3283	AB57_RS01645	ABUW_3651
AB57_RS04705	ABUW_3057	AB57_RS03565	ABUW_3287	AB57_RS00945	ABUW_3740
AB57_RS04700	ABUW_3058	AB57_RS03515	ABUW_3297	AB57_RS00940	ABUW_3741
AB57_RS04590	ABUW_3078	AB57_RS03490	ABUW_3302	AB57_RS00935	ABUW_3742
AB57_RS04565	ABUW_3083	AB57_RS03400	ABUW_3320	AB57_RS00930	ABUW_3743
AB57_RS04550	ABUW_3086	AB57_RS03380	ABUW_3324	AB57_RS00580	ABUW_3815
AB57_RS04545	ABUW_3087	AB57_RS03285	ABUW_3344	AB57_RS00570	ABUW_3817
AB57_RS04475	ABUW_3101	AB57_RS03270	ABUW_3347	AB57_RS00565	ABUW_3818
AB57_RS04340	ABUW_3129	AB57_RS03255	ABUW_3350	AB57_RS00560	ABUW_3819
AB57_RS04335	ABUW_3130	AB57_RS03245	ABUW_3352	AB57_RS00540	ABUW_3820
AB57_RS04315	ABUW_3134	AB57_RS03205	ABUW_3360	AB57_RS00465	ABUW_3833
AB57_RS04295	ABUW_3138	AB57_RS03170	ABUW_3367	AB57_RS00450	ABUW_3836
AB57_RS04280	ABUW_3141	AB57_RS03135	ABUW_3374	AB57_RS00425	ABUW_3841
AB57_RS04275	ABUW_3142	AB57_RS03100	ABUW_3381	AB57_RS00400	ABUW_3846
AB57_RS04270	ABUW_3143	AB57_RS03080	ABUW_3385	AB57_RS00315	ABUW_3863
AB57_RS04265	ABUW_3144	AB57_RS02975	ABUW_3406	AB57_RS00240	ABUW_3878
AB57_RS04185	ABUW_3160	AB57_RS02965	ABUW_3408	AB57_RS00090	ABUW_4003
AB57_RS04180	ABUW_3161	AB57_RS02755	ABUW_3431	AB57_RS00085	ABUW_4004
AB57_RS04110	ABUW_3175	AB57_RS02660	ABUW_3447	AB57_RS00080	ABUW_4005
AB57_RS04095	ABUW_3178	AB57_RS02620	ABUW_3455	AB57_RS00075	ABUW_4006
AB57_RS04085	ABUW_3180	AB57_RS02605	ABUW_3458	AB57_RS00030	ABUW_4009
AB57_RS04080	ABUW_3181	AB57_RS02590	ABUW_3461	AB57_RS00010	ABUW_4013
AB57_RS04070	ABUW_3183	AB57_RS02560	ABUW_3467	AB57_RS19380	ABUW_4021
AB57_RS04055	ABUW_3186	AB57_RS02525	ABUW_3474	AB57_RS19370	ABUW_4023
AB57_RS04010	ABUW_3197	AB57_RS02505	ABUW_3478	AB57_RS19360	ABUW_4025

Table S5. continued.

With AB5075 (*Galleria mellonella* infection model)[c]	
**AB0057**	**AB5075**
**Locus_tag**	**Locus_tag**
AB57_RS19355	ABUW_4026
AB57_RS19350	ABUW_4027
AB57_RS19340	ABUW_4029
AB57_RS19335	ABUW_4030
AB57_RS19330	ABUW_4031
AB57_RS19325	ABUW_4033
AB57_RS19305	ABUW_4037
AB57_RS19280	ABUW_4042
AB57_RS19135	ABUW_4074
AB57_RS19130	ABUW_4075
AB57_RS19110	ABUW_4079
AB57_RS19105	ABUW_4080
AB57_RS19100	ABUW_4081
AB57_RS19095	ABUW_4082
AB57_RS19090	ABUW_4083
AB57_RS19065	ABUW_4090
AB57_RS19060	ABUW_4091
AB57_RS19040	ABUW_4095
AB57_RS19015	ABUW_4100
AB57_RS19005	ABUW_4102
AB57_RS18995	ABUW_4104
AB57_RS18985	ABUW_4106
AB57_RS18980	ABUW_4107
AB57_RS18960	ABUW_4111
AB57_RS18955	ABUW_4112
AB57_RS18950	ABUW_4113
AB57_RS18940	ABUW_4115
AB57_RS18925	ABUW_4118

Reference

[a] Wang, N., Ozer, E.A., Mandel, M.J., and Hauser, A.R. (2014) Genome-wide identification of *Acinetobacter baumannii* genes necessary for persistence in the lung. *MBio* **5**: e01163-01114.

[b] Subashchandrabose, S., Smith, S., DeOrnellas, V., Crepin, S., Kole, M., Zahdeh, C., and Mobley, H.L. (2016) *Acinetobacter baumannii* genes required for bacterial survival during bloodstream infection. *mSphere* **1**: e00013-15.

[c] Gebhardt, M.J., Gallagher, L.A., Jacobson, R.K., Usacheva, E.A., Peterson, L.R., Zurawski, D.V., and Shuman, H.A. (2015) Joint transcriptional control of virulence and resistance to antibiotic and environmental stress in *Acinetobacter baumannii*. MBio 6: e01660-01615.

Table S6. Strains and plasmids used in this study.

Strains	Characteristic(s)[a]	Source or reference
E. coli		
MGN-617	*hi thr leu tonA lacY glnV supE* Δ*asdA4 recA*::RP4 2-Tc::Mu [pir]; Km^r	(Dozois *et al.*, 2000)
DY330	W3110 Δ*lacU169 gal490* λ*cI857* Δ(*cro-bioA*)	(Yu *et al.*, 2000)
DH5 α λ pir	sup E44, Δ*lacU169* (ΦlacZΔM15), *recA1*, *endA1*, *hsdR17*, *thi-1*, *gyrA96*, *relA1*, λpir phage lysogen	Laboratory collection
A. baumannii		
AB0057	MDR bloodstream isolate	(Hujer *et al.*, 2006)
AB0057^{Km}; 57; WT	AB0057 Δ*km*::FRT; Km susceptible	This study
Δ*hcaR*	AB0057^{Km} Δ*hcaR*	This study
Δ*0044*	AB0057^{Km} Δ*AB57_0044*	This study
Δ*vipAB*	AB0057^{Km} Δ*vipAB*	This study
Δ*filA*	AB0057^{Km} Δ*filA*	This study
Δ*bfmR*	AB0057^{Km} Δ*bfmR*	This study
Δ*gltP*	AB0057^{Km} Δ*gltP*	This study
57 Δ*mltB* (*AB57_2749*)	AB0057^{Km} Δ*mltB*	This study
57 eV	AB0057^{Km} + pABBR_Km; Km^R	This study
57 Δ*mltB* eV	AB0057^{Km} Δ*mltB* + pABBR_Km; Km^R	This study
57 Δ*mltB* compl.	AB0057^{Km} Δ*mltB* + pABBR_Km-*mrdB-mltB*; Km^R	This study
AB5075	MDR Tibia/osteomyelitis isolate	(Jacobs *et al.*, 2014)
75 Δ*mltB*	ABUW Δ*1243*::T26 tnab1_kr130917p02q159	(Gallagher *et al.*, 2015)
**Plasmids**		
pKD3	Template plasmid for the amplification of the Cm cassette bordered by FRT sites; Ap^r Cm^r	(Datsenko & Wanner, 2000)
pKD4	Template plasmid for the amplification of the Km cassette bordered by FRT sites; Ap^r Km^r	(Datsenko & Wanner, 2000)
pSU2719	P15A replicon, *lacZ*α, Cm^r	(Martinez *et al.*, 1988)

pKD_Zeo	Template plasmid for the amplification of the Zeo cassette bordered by FRT sites; Ap^r Zeo^r	This study
pSUABKm	pSU2719-*AB57_0288*; Cm^r, Km^r	This study
pSU ΔABKm::*zeo*	pSU2719-Δ*AB57_0288*::Zeo; Cm^r, Km^r	This study
pAT03	pMMB67EH with FLP recombinase; Ap^r	(Tucker *et al.*, 2014)
pAT03_Km	pAT03_Km; Km^r	This study
pCVD442	Suicide vector containing *sacB*; Ap^r	(Donnenberg & Kaper, 1991)
pCVD442_MCS	pCVD442 + MCS; The IS*1* element was swapped by a MCS; Ap^r, R6K, *sacB*	This study
pCVD_Amk	pCVD442_MCS_Amk; Amk^r, R6K, *sacB*	This study
pCVD_AmK_Δ*hcaR*	Δ*hcaR* in pCVD_AmK, Amk^r, R6K, *sacB*	This study
pCVD_AmK_Δ*AB57_0044*	Δ*AB57_0044* in pCVD_AmK; Amk^r, R6K, *sacB*	This study
pCVD_AmK_Δ*vipAB*	Δ*vipAB* in pCVD_AmK; Amk^r, R6K, *sacB*	This study
pCVD_AmK_Δ*filA*	Δ*filA* in pCVD_AmK; Amk^r, R6K, *sacB*	This study
pCVD_AmK_Δ*bfmR*	Δ*bfmR* in pCVD_AmK; Amk^r, R6K, *sacB*	This study
pCVD_AmK_Δ*gltP*	Δ*gltP* in pCVD_AmK; Amk^r, R6K, *sacB*	This study
pABBR_MCS	*ori* of pWH1266 cloned into pBR322; Tet^r, Ap^r	(Tucker *et al.*, 2014)
pABBR_Km	pABBR_MCS_Km; Km^r	This study
pABBR_Km-mrdB-mltB	Genes mrdB-mltB cloned into pABBR_Km; Km^r	This study

REFERENCES

Datsenko, K.A., and Wanner, B.L. (2000) One-step inactivation of chromosomal genes in *Escherichia coli* K-12 using PCR products. *Proc Natl Acad Sci USA* **97**: 6640-6645.

Donnenberg, M.S., and Kaper, J.B. (1991) Construction of an *eae* deletion mutant of Enteropathogenic *Escherichia coli* by using a positive-selection suicide vector. *Infect Immun* **59**: 4310-4317.

Dozois, C.M., Dho-Moulin, M., Bree, A., Fairbrother, J.M., Desautels, C., and Curtiss, R. (2000) Relationship between the Tsh autotransporter and pathogenicity of avian *Escherichia coli* and localization and analysis of the *tsh* genetic region. *Infect Immun* **68**: 4145-4154.

Gallagher, L.A., Ramage, E., Weiss, E.J., Radey, M., Hayden, H.S., Held, K.G., Huse, H.K., Zurawski, D.V., Brittnacher, M.J., and Manoil, C. (2015) Resources for genetic and genomic analysis of emerging pathogen *Acinetobacter baumannii*. *J Bacteriol* **197**: 2027-2035.

Hujer, K.M., Hujer, A.M., Hulten, E.A., Bajaksouzian, S., Adams, J.M., Donskey, C.J., Ecker, D.J., Massire, C., Eshoo, M.W., Sampath, R., Thomson, J.M., Rather, P.N., Craft, D.W., Fishbain, J.T., Ewell, A.J., Jacobs, M.R., Paterson, D.L., and Bonomo, R.A. (2006) Analysis of antibiotic resistance genes in multidrug-resistant *Acinetobacter sp* isolates from military and civilian patients treated at the Walter Reed Army Medical Center. *Antimicrob Agents Chem* **50**: 4114-4123.

Jacobs, A.C., Thompson, M.G., Black, C.C., Kessler, J.L., Clark, L.P., McQueary, C.N., Gancz, H.Y., Corey, B.W., Moon, J.K., Si, Y., Owen, M.T., Hallock, J.D., Kwak, Y.I., Summers, A., Li, C.Z., Rasko, D.A., Penwell, W.F., Honnold, C.L., Wise, M.C., Waterman, P.E., Lesho, E.P., Stewart, R.L., Actis, L.A., Palys, T.J., Craft, D.W., and Zurawski, D.V. (2014) AB5075, a highly virulent isolate of *Acinetobacter baumannii*, as a model strain for the evaluation of pathogenesis and antimicrobial treatments. *MBio* **5**: e01076-01014.

Martinez, E., Bartolome, B., and de la Cruz, F. (1988) pACYC184-derived cloning vectors containing the multiple cloning site and *lacZ* alpha reporter gene of pUC8/9 and pUC18/19 plasmids. *Gene* **68**: 159-162.

Tucker, A.T., Nowicki, E.M., Boll, J.M., Knauf, G.A., Burdis, N.C., Trent, M.S., and Davies, B.W. (2014) Defining gene-phenotype relationships in *Acinetobacter baumannii* through one-step chromosomal gene inactivation. *MBio* **5**: e01313-01314.

Yu, D.G., Ellis, H.M., Lee, E.C., Jenkins, N.A., Copeland, N.G., and Court, D.L. (2000) An efficient recombination system for chromosome engineering in *Escherichia coli. Proc Natl Acad Sci USA* **97**: 5978-5983.

Table S7. Primers and oligonucleotides.

Name	Sequence 5' → 3'	Purpose
**TraDIS**		
Tn-specific primer	AATGATACGGCGACCACCGAGATCTACAAGGCATGCAAGCTTCAGGGTTGAGATGTGTATAAG	Tn-gDNA junction amplification
Pri_Adap_#1_R	CAAGCAGAAGACGGCATACGAGACGTGATGTGACTGGA	Tn-gDNA junction amplification of input #1 from pool #1. Used with Tn-specific primer.
Pri_Adap_#2_R	CAAGCAGAAGACGGCATACGAGATACATCGGTGACTGGA	Tn-gDNA junction amplification of input #2 from pool #1. Used with Tn-specific primer.
Pri_Adap_#3_R	CAAGCAGAAGACGGCATACGAGATGCCTAAGTGACTGGA	Tn-gDNA junction amplification of input #1 from pool #2. Used with Tn-specific primer.
Pri_Adap_#4_R	CAAGCAGAAGACGGCATACGAGATTGGTCAGTGACTGGA	Tn-gDNA junction amplification of input #2 from pool #2. Used with Tn-specific primer.
Pri_Adap_#5_R	CAAGCAGAAGACGGCATACGAGATCACTGTGTGACTGGA	Tn-gDNA junction amplification of input #1 from pool #3. Used with Tn-specific primer.
Pri_Adap_#6_R	CAAGCAGAAGACGGCATACGAGATATTGGCGTGACTGGA	Tn-gDNA junction amplification of input #2 from pool #3. Used with Tn-specific primer.
Pri_Adap_#7_R	CAAGCAGAAGACGGCATACGAGATGATCTGGTGACTGGA	Tn-gDNA junction amplification of input #1 from pool #4. Used with Tn-specific primer.
Pri_Adap_#8_R	CAAGCAGAAGACGGCATACGAGATTCAAGTGTGACTGGA	Tn-gDNA junction amplification of input #2 from pool #4. Used with Tn-specific primer.
Pri_Adap_#9_R	CAAGCAGAAGACGGCATACGAGATCTGATCGTGACTGGA	Tn-gDNA junction amplification of input #1 from pool #5. Used with Tn-specific primer.
Pri_Adap_#10_R	CAAGCAGAAGACGGCATACGAGATAAGCTAGTGACTGGA	Tn-gDNA junction amplification of input #2 from pool #5. Used with Tn-specific primer.
Pri_Adap_#11_R	CAAGCAGAAGACGGCATACGAGATGTAGCCGTGACTGGA	Tn-gDNA junction amplification of output #1 from pool #1. Used with Tn-specific primer.
Pri_Adap_#12_R	CAAGCAGAAGACGGCATACGAGATTACAAGGTGACTGGA	Tn-gDNA junction amplification of output #2 from pool #1. Used with Tn-specific primer.
Pri_Adap_#13_R	CAAGCAGAAGACGGCATACGAGATTGTTGACTGTGACTGGA	Tn-gDNA junction amplification of output #3 from pool #1. Used with Tn-specific primer.
Pri_Adap_#14_R	CAAGCAGAAGACGGCATACGAGATACGGAACTGTGACTGGA	Tn-gDNA junction amplification of output #4 from pool #1. Used with Tn-specific primer.

Pri_Adap_#15_R	CAAGCAGAAGACGGCATACGAGATTCTGACATGTGACTGGA	Tn-gDNA junction amplification of output #1 from pool #2. Used with Tn-specific primer.
Pri_Adap_#16_R	CAAGCAGAAGACGGCATACGAGATCGGGACGGGTGACTGGA	Tn-gDNA junction amplification of output #2 from pool #2. Used with Tn-specific primer.
Pri_Adap_#17_R	CAAGCAGAAGACGGCATACGAGATCTCTACGTGACTGGA	Tn-gDNA junction amplification of output #3 from pool #2. Used with Tn-specific primer.
Pri_Adap_#18_R	CAAGCAGAAGACGGCATACGAGATGTGCGGACGTGACTGGA	Tn-gDNA junction amplification of output #4 from pool #2. Used with Tn-specific primer.
Pri_Adap_#19_R	CAAGCAGAAGACGGCATACGAGATCGTTTCACGTGACTGGA	Tn-gDNA junction amplification of output #1 from pool #3. Used with Tn-specific primer.
Pri_Adap_#20_R	CAAGCAGAAGACGGCATACGAGATAAGGCCACGTGACTGGA	Tn-gDNA junction amplification of output #2 from pool #3. Used with Tn-specific primer.
Pri_Adap_#21_R	CAAGCAGAAGACGGCATACGAGATTCCGAAACGTGACTGGA	Tn-gDNA junction amplification of output #3 from pool #3. Used with Tn-specific primer.
Pri_Adap_#22_R	CAAGCAGAAGACGGCATACGAGATTACGTACGGTGACTGGA	Tn-gDNA junction amplification of output #4 from pool #3. Used with Tn-specific primer.
Pri_Adap_#23_R	CAAGCAGAAGACGGCATACGAGATATCCACTCGTGACTGGA	Tn-gDNA junction amplification of output #1 from pool #4. Used with Tn-specific primer.
Pri_Adap_#24_R	CAAGCAGAAGACGGCATACGAGATGCTACCGTGACTGGA	Tn-gDNA junction amplification of output #2 from pool #4. Used with Tn-specific primer.
Pri_Adap_#25_R	CAAGCAGAAGACGGCATACGAGATATATCAGTGTGACTGGA	Tn-gDNA junction amplification of output #3 from pool #4. Used with Tn-specific primer.
Pri_Adap_#26_R	CAAGCAGAAGACGGCATACGAGATGCTCATGTGACTGGA	Tn-gDNA junction amplification of output #4 from pool #4. Used with Tn-specific primer.
Pri_Adap_#27_R	CAAGCAGAAGACGGCATACGAGATAAAGGAATGTGACTGGA	Tn-gDNA junction amplification of output #1 from pool #5. Used with Tn-specific primer.
Pri_Adap_#28_R	CAAGCAGAAGACGGCATACGAGATCTTTTGGTGACTGGA	Tn-gDNA junction amplification of output #2 from pool #5. Used with Tn-specific primer.
Pri_Adap_#29_R	CAAGCAGAAGACGGCATACGAGATTAGTTGGTGACTGGA	Tn-gDNA junction amplification of output #3 from pool #5. Used with Tn-specific primer.
Pri_Adap_#30_R	CAAGCAGAAGACGGCATACGAGATCCGGTGGTGACTGGA	Tn-gDNA junction amplification of output #4 from pool #5. Used with Tn-specific primer.

Sequencing primer	GCATGCAAGCTTCAGGGTTGAGATGTGTATAAG	Sequencing the Tn-gDNA junction
**Plasmids**		
pKD_R_BamH1	GGGG**GGATCC**GCGCGCCATTTAAATGAAGTTCCT	Inverted PCR of pKD3 to remove the *cat* cassette
pKD_F_for_Bpu10I	GAGCAACTGACTGAAATGCCTCAAA*	
sh_ble_F(2)_BamH1	CATA**GGATCC**GCTACCCGTGATATTGCTGAAGAG	Amplification of sh_ble (Zeo) from pCR-BluntII-TOPO and cloned into the pKD-Δ*cat*, creating of pKD_Zeo
sh_ble_R_Bpu10I	TCTTTTC**GCTAAGG**TCTGACGCTCAGTGGAACGAAA	
AB57_0288_5'_F_Kpn1	ACC**GGTACC**TGCGTGTGCTGCAAGGAAATC	Amplification of *AB57_0288* and cloning into pSU2719, creating pSU_ABKm
AB57_0288_3'_R_Sph1	TGC**GCATGC**TCGCATGGTGTTCGGAATCT	
AB57_0288_F_KO	GAACAATAAAACTGTCTGCTTACATAAACAGTAATACAAGGGGTGTTATGTGTGTAGGCTGGAGCTGCTTC	KO of *AB57_0288* in pSU_ABKm by recombineering, creating pSU_ΔABKmsh_ble
AB57_0288_R_KO	CAGGTTTGTGTCTCTACAAAGACTAACTATCAGAAAAACTCATCGAGCATTGGTCCATATGAATATCCTCCTTAGTTCC	
Km_F_pvuI	CAAG**CGATCG**TAAAGGAAGCGGAACACGTAGAAAG	Amplification of the *kan* cassette from pKD4 and cloning into pABBR_MCS, creating pABBR_Km
Km_R_SalI	GTCC**GTCGAC**ACGATTCCGAAGCCCAACCTTTCA	
Amk_F_AatII	ATCG**GACGTC**AGGACAGAAATGCCTCGACTTC	Amplification of the amikacin cassette (gene *ABAYE3630*), and cloned into pCVD_MCS
Amk_R_Bsa1	CAATGATACCGC**GAGACC**CACCGGGCTTTAAACTCGCCTCTAT	
2749_F_compl_PspX1	AACTAA**ACTCGAGG**AGTCTATCGGAAACCTGGTGAGAG	Cloning the *mdrB-mltB* locus into pABBR_Km vector.
2749_R_compl_NruI	GGGTGG**TCGCGA**TTCATCGCTTACGGAGAGTGTCT	

**Mutants**		
KO_AB57_0044_F _for_XbaI_screen_ (frag_5’)	ACACCTGTTGCAGATGTAGATGTGTT*	Amplification of ~ 1 Kb upstream of *AB57_0044*
KO_AB57_0044_R_ PvuI-HF_(frag_5’)	ACAAT**CGATCG**AAAACGTCGCATATTATCC TAGTTCAATTT	
KO_AB57_0044_F_ PvuI-HF_(frag_3’)	GCCAA**CGATCG**GCACGAAAAATGATGCAA TAAAATATTAAATAAAAAAGG	Amplification of ~ 1 Kb downstream of *AB57_0044*
KO_AB57_0044_R_ Sac1 _(frag_5’)	CGTT**GAGCTC**CTTTGTTATTAGGCATGGCC TTTGC	
KO_AB57_0044_R_ screen	ACTTGCTCATGAGTTTGGACATGC	Screening to confirm the *AB57_0044* mutation; used with KO_AB57_0044_F_for_XbaI_s creen_(frag_5’)
KO_AB57_2749_F_ Sac1 _(frag_5’)	CGTA**GAGCTC**AGTCTATCGGAAACCTGGT GAGAG	Amplification of ~ 1 Kb upstream of *AB57_2749*
KO_AB57_2749_R_ MluI-Hf _(frag_5’)	TTAAC**ACGCGT**AGACAACATATCTTTTTTAA ATTCCAGATTATCGAT	
KO_AB57_2749_F_ MluI-HF_(frag_3’)	GGTTG**ACGCGT**ACAGAAGTAGCTAGCCGA TAGGCT	Amplification of ~ 1 Kb downstream of *AB57_2749*
KO_AB57_2749_R_ for_XbaI_screen_(fr ag_3’)	AGTGGTACTAGGGTAGCGGAATTAT*	
KO_AB57_2749_F_ screen	CTAAAGCAGTTCATATTGCGATCGACA	Screening to confirm the *AB57_0044* mutation; used with KO_AB57_2749_R_for_XbaI_s creen_(frag_3’)
KO_vipAB_F_MluI_ (frag_5')	CGGC**ACGCGT**CATAAAAGTTAGCCTTGTTC CTTACAAATTGT	Amplification of ~ 1 Kb upstream of *vipAB*
KO_vipAB_R_Sal1_ (frag_5')	TTTTT**GTCGAC**TCGCGTGTACTGACATAAA TAGTGTC	
KO_vipAB_R_MluI_ (frag_3')	GTATC**ACGCGT**GCAATCAACTGGTATAGTA CTAAAATTTAGTCTTTGAAA	Amplification of ~ 1 Kb downstream of *vipAB*
KO_vipAB_F_Sac1_ (frag_3')	CGCT**GAGCTC**TCCCTTCGGCCTGTATCAAC AT	
KO_vipAB_screen_F	TATCCTTTGTTTCATCTTCGCAATTTCAC	Screening to confirm the *vipAB* mutation

KO_vipAB_screen_R	TAAATACGACTGGCGCTTGCAG	
KO_RRcsu_fim_R_MluI_(Frag_5)	TTTC**ACGCGT**GCTCATATCATTGCCCCTATAAATCTCATT	Amplification of ~ 1 Kb downstream of *bfmR*
KO_RRcsu_fim_F_PvuI_(Frag_5)	TGCCA**CGATCG**TGGTGGTAGCAATATCGCTTTCTTT	
KO_RRcsu_fim_F_Xba1_(Frag_3')	TATCTTTATC**TCTAGA**GTCGACCTGATGCATACGATGATT	Amplification of ~ 1 Kb upstream of *bfmR*
KO_RRcsu_fim_R_MluI_(Frag_3')	**ACGCGT**GTTAAAGAAACCAATGGATTGTAAAATCTGATTAAACTT	
KO_RRcsu_fim_F_screen	ATCTAGTGAAGGTGTACCCTGCTC	Screening to confirm the *bfmR* mutation
KO_RRcsu_fim_R_screen	CTCATCACGAAGCAAACGTGCA	
KO_FilA_F_MluI_(Frag_5)	GTGAA**ACGCGT**CATTGAGCACTCTCCCAAGAGC	Amplification of ~ 1 Kb upstream of *filA*
KO_FilA_R_Sac1_(Frag_5)	GTAT**GAGCTC**TTAGACCAGCGAGGATGAATATTTTGTTCG	
KO_FilA_R_MluI_(Frag_3)	ATGTG**ACGCGT**ACAACTATCACAATATCTGGTCATTAATTTAAATATAAAAAAAGC	Amplification of ~ 1 Kb downstream of *filA*
KO_FilA_F_Xba1_(Frag_3)	CAACAAAAGC**TCTAGA**ATTGACTGTTGCATCCTCTACATGAC	
KO_FilA_F_screen	CGCATCGTAGAAGCCTTATTTGCT	Screening to confirm the *filA* mutation
KO_FilA_R_screen	ATAACCAGAATACGGTGCATTGGT	
KO_hcaR_R_MluI_(frag_5)	AGATG**ACGCGT**TTCCATAAGACTAATCCAAAAAATGTCTTACTATGAT	Amplification of ~ 1 Kb upstream of *hcaR*
KO_hcaR_F_Sal1_(frag_5)	ATCC**GTCGAC**AGGCGATCTAGGCTGCTGATTA	
KO_hcaR_F_MluI_(frag_3)	ACAAA**ACGCGT**CATCTTGATAAAAATCTAGATTAAGCACCTTAGGT	Amplification of ~ 1 Kb downstream of *hcaR*
KO_hcaR_R_Sac1_(frag_3)	TGTTG**GAGCTC**TCAAACAGCGCGAAGGCTTAAAAC	
KO_HcaR_screen_F	ATAAGACTCACGAGTGACTAACCAACG	

KO_HcaR_screen_R	GGACTGAGTGAAGCAAATCCGTATG	Screening to confirm the *hcaR* mutation
KO_1698_F_Mlu1_Reg5'	aatttctt**ACGCGT**cataggaacctctatcctttgggttc	Amplification of ~ 1 Kb upstream of *gltP*
KO_1698_R_Sac1_Reg5'	ttgtca**GAGCTC**atccgaacaacccaactggttc	
KO_1698_F_Xba1_Reg3'	gagcagttt**TCTAGA**cgcattgagccaacaagtacga	Amplification of ~ 1 Kb downstream of *gltP*
KO_1698_R_Mlu1_Reg3'	taaa**ACGCGT**caagaacaaacaatcgcttaagtttcct	
KO_1698_F_screen	atcagaagcggccaatgcaa	Screening to confirm the *gltP* mutation
KO_1698_R_screen	tcattccgatgccggactatcc	
**qRT-PCR**		
**ESR**		
degP_F_qPCR	GTAATCAGAGCACCTTCCGGTTTAG	Amplification of a portion of *degP*
degP_R_qPCR	TGACTCGTTCATACCTAGGCGTTAT	
baeR_F_qPCR	TGTTCACGTACCTTACGGCAAATC	Amplification of a portion of *baeR*
baeR_R_qPCR	GGTCAGGATGCTTACACGAACTTT	
rstA_F_qPCR	CGCCCACACTATCATCAACCAA	Amplification of a portion of *rstA*
rstA_R_qPCR	GCACCCATTTCCAGACCAAGTA	
dsbA_F_qPCR	GGGTAGTGTAATGGCAGCAGATTT	Amplification of a portion of *dsbA*
dsbA_R_qPCR	GGTACTTCCACTTTGCCTGGATTG	
**LT**		
LT_0044_F_qPCR	GAAGGACGCCGGCAAAGTTATT	Amplification of a portion of *AB57_0044*
LT_0044_R_qPCR	CCAAATGCTCCTGCCCATGAA	
LT_1068_F_qPCR	GTGAAGTTGTTGCTGGTGGTTCTACC	Amplification of a portion of *AB57_1068*
LT_1068_R_qPCR	ATGCGCTCCATCATCCATGT	
LT_1136_F_qPCR	CCGACCACAACAACTTCAACCT	Amplification of a portion of *AB57_1136*
LT_1136_R_qPCR	TACCGTTACCGAGCTGCTACTT	
LT_2801_F_qPCR	GCCAGCGGTTACATGGACATTAG	Amplification of a portion of *AB57_2801*
LT_2801_R_qPCR	ATAACGTGAGCGCGTGCATAAA	
LT_3476_F_qPCR	CGTTGTACGATCTGCCGCATAAA	Amplification of a portion of *AB57_3476*
LT_3476_R_qPCR	ACCCAACCTATACCAACCGAGAG	
**PBP**		
PBP_0326_F_qPCR	TCCAATCCGTGATACTGTGGGTTAT	

PBP_0326_R_qPCR	CGGCACTGATGCGTTGGTATTT	Amplification of a portion of *AB57_0326*
pbp2_F_qPCR	CCGGTTTAATTCCAGCGGTCAT	Amplification of a portion of *pbp2*
pbp2_F_qPCR	TCAGCAGCCCAACTTGCTAAAC	
PBP_2186_F_qPCR	AGCTCAAGCTCGCATTGCTTTA	Amplification of a portion of *AB57_2186*
PBP_2186_R_qPCR	CATCCTTGCTGTGGGCCATTAT	
pbp1B_F_qPCR	CGGCTTTAACCGTGCCTTAGAT	Amplification of a portion of *pbp1B*
pbp1B_R_qPCR	GGAACGCTAATTGGAGCGTCTT	
PBP_2863_F_qPCR	CTGACCCAAGTGTTGATGGCTT	Amplification of a portion of *AB57_2863*
PBP_2863_R_qPCR	CACGTTCAGACATGCTTGGTGTA	
pbp1A_F_qPCR	CGTGGTGCAGAAGCACATGATA	Amplification of a portion of *pbp1A*
pbp1A_R_qPCR	CACGGTTACTGTTGAGCCATCTT	

Underlined nucleotides denote barcode sequence
Bold nucleotides denote restriction site
*: Restriction site is natively encoded within the amplified product
